# Identification of Substituted Amino Acid Hydrazides as Novel Anti-Tubercular Agents, Using a Scaffold Hopping Approach

**DOI:** 10.3390/molecules25102387

**Published:** 2020-05-21

**Authors:** Alistair K. Brown, Ahmed K. B. Aljohani, Fatimah M. A. Alsalem, Joseph L. Broadhead, Jason H. Gill, Yucheng Lu, Jonathan D. Sellars

**Affiliations:** 1 Biosciences Institute, Faculty of Medical Sciences, Newcastle University, Newcastle upon Tyne NE2 4HH, UK; alistair.brown2@newcastle.ac.uk (A.K.B.); a.aljohani2@newcastle.ac.uk (A.K.B.A.); f.m.a.alsalem2@newcastle.ac.uk (F.M.A.A.); y.lu48@newcastle.ac.uk (Y.L.); 2 Arcinova, Taylor Drive, Alnwick NE66 2DH, UK; joseph190597@googlemail.com; 3 Translational and Clinical Research Institute, Faculty of Medical Sciences, Newcastle University, Newcastle upon Tyne NE2 4HH, UK; jason.gill@newcastle.ac.uk; 4 School of Pharmacy, Faculty of Medical Sciences, King George VI Building, Newcastle upon Tyne NE1 7RU, UK

**Keywords:** antibacterial, *Mycobacterium tuberculosis*, amino acid, hydrazide, Q203, Naphthalene, Quinoline, scaffold hopping

## Abstract

Discovery and development of new therapeutic options for the treatment of *Mycobacterium tuberculosis* (*Mtb*) infection, particularly drug-resistant strains, are urgently required to tackle the global burden of this disease. Herein, we reported the synthesis of a novel series of N-substituted amino acid hydrazides, utilising a scaffold hopping approach within a library of anti-tubercular agents. Efficacy and selectivity were evaluated against three strains of *Mtb* (wild-type, isoniazid-resistant and rifampicin-resistant), and cytotoxicity against macrophages in vitro. The antibacterial activity and therapeutic index of these molecules were significantly affected by modifications with the N-substituents. Introduction of a 3,5-dinitroaryl moiety demonstrated enhanced antibacterial activity against all three strains of *Mtb*. In contrast, the inclusion of an imidazo [1,2-a]pyridine-3-carboxy moiety resulted in enhanced activity towards isoniazid mono-resistant *Mtb* relative to wild-type *Mtb*. Consequently, this scaffold hopping approach showed significant promise for exemplification of novel molecules with specific activity profiles against drug-resistant tuberculosis.

## 1. Introduction

The World Health Organisatio*N′*s (WHO) global tuberculosis report in 2019 recognises tuberculosis (TB) as a communicable disease responsible for a major cause of long term illness and continues to be one of the top ten causes of death worldwide from a single infectious agent [1]. In 2018, an estimated ten million (range 9.0–11.1 million) new cases of TB infection were reported, equivalent to 132 cases per 100,000 population globally [2]. Unfortunately, treatment responses to TB are stagnating, and most WHO regions are now likely to fail to reach the 2020 milestone of the WHO′s End TB strategy [2]. Concerningly, there is an ever-increasing number of infections from strains resistant to the front line anti-TB drugs—rifampicin (RIF) and isoniazid (INH), exacerbating further the global TB epidemic [1]. Consequently, new drug molecules with novel modes of action are urgently required to address this increased disease burden and diminishing response to current therapies.

Over the past ten years, notable advances towards the development of new therapies against TB have been made. For instance, the dihydro-nitroimidazooxazole derivative—Delamanid (Deltyba^®®^)—acts as a prodrug activated by the mycobacterial deazaflavin-dependent nitroreductase to an inhibitor of methoxy mycolic acid and ketomycolic acid synthesis of the mycobacterial cell wall [3]. Another such drug, dinitrobenzamide (DNB-1) has shown a novel mechanism involving inhibition of decaprenylphosphoryl-β-d-ribose 2′-epimerase (DprE1), a vital enzyme in cell wall lipoarabinomannan and arabinogalactan precursor biosynthesis [4]. Conversely, pyrazinamide, a clinically utilised drug since 1952 demonstrates activity against dormant or semi-dormant TB, with a mode of action involving multiple cellular targets focusing on the disruption of energy production and fatty acid synthesis [5]. However, despite the significant promise, resistance to several of these molecules has now been reported [5,6,7,8,9].

**Figure 1:**
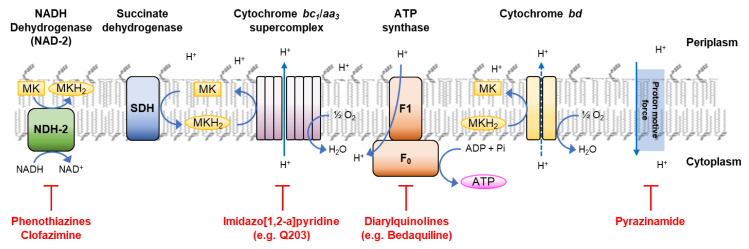
Respiratory electron transport chain (ETC) in *Mycobacterium tuberculosis* (*Mtb*). Electrons derived from NADH are fed into the electron transport chain by the type II NADH dehydrogenase (NDH-2), leading to the reduction of the menaquinone pool (MK/MKH_2_). The menaquinone pool can also be reduced by alternative electron donors, e.g., the succinate dehydrogenase (SDH). The menaquinone pool transfers electrons to the cytochrome bc1 complex. The cytochrome bc1 complex forms a supercomplex with the cytochrome aa3-type terminal oxidase, which transfers the electrons onto oxygen. Additionally, oxygen can be reduced by the cytochrome bd-type terminal oxidase, which directly accepts electrons from the menaquinone pool. Electron transport along the ETC promotes proton motive force; this energy is utilised by ATP synthase, resulting in the synthesis of ATP.

A notable new direction for the development of anti-TB drugs has been the perturbation of the mycobacterial respiratory electron transport chain (ETC), with several such drugs entering the clinic recently (Figure 1) [10]. The diarylquinoline Bedaquiline (SIRTURO^®®^), used predominantly against drug-resistant TB, targets the mycobacterial respiratory chain enzyme F1/F0-ATPase [11,12]. Whereas the 1,2-ethylene diamine SQ109, shown to be active against both drug-sensitive and drug-resistant TB, has a mechanism involving inhibition of the Mycobacterial membrane protein Large 3 (MmpL3)-dependent export of mycolic acids but has also been linked to the dissipation of the proton motive force within the ETC [13]. More recently, the first in class drug—Q203 (Telacebec^®®^), has shown a novel mechanism of action targeting the cytochrome *bc1* complex of the ETC and has demonstrated significant promise in the clinic [14]. Furthermore, the therapeutic mechanism of the ‘old′ drug clofazimine, which has been repurposed for treatment of drug-resistant TB, also inhibits the NADH dehydrogenase (NAD-2) within the ETC [15]. Consequently, in order to tackle the problematic exacerbation of drug-resistant TB, the identification of drug targets outside of the conventional pathways for current drugs and subsequent discovery of drug molecules exploiting these new therapeutic avenues are required.

**Figure 2:**
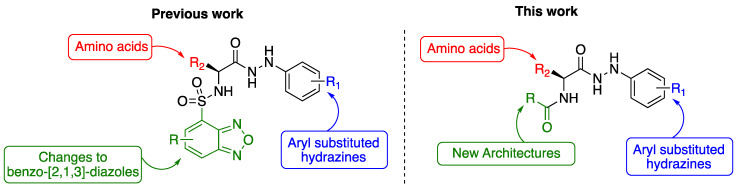
Inhibitors of *M. tuberculosis* based on a benzoxa-[2,1,3]-diazole framework, highlighting the key modifications.

Our previous studies of benzoxa-[2,1,3]-diazole-substituted amino acid hydrazides demonstrated therapeutic selectivity of these molecules against *mycolata* over Gram-positive and Gram-negative bacteria [16]. This highlighted the involvement of the benzoxa-[2,1,3]-diazole, the amino acid (R_2_) moiety and the substituted aryl hydrazine (R_1_) towards bacterial selectivity, efficacy and establishment of a therapeutic index for development as a drug [16]. In this study, we evaluated variation of the benzoxa-[2,1,3]-diazole moiety within these compounds via the introduction of new architectures and their structure-activity-relationships toward the development of efficacious drugs against drug-resistant *Mtb* (Figure 2).

### Strategy

**Figure 3:**
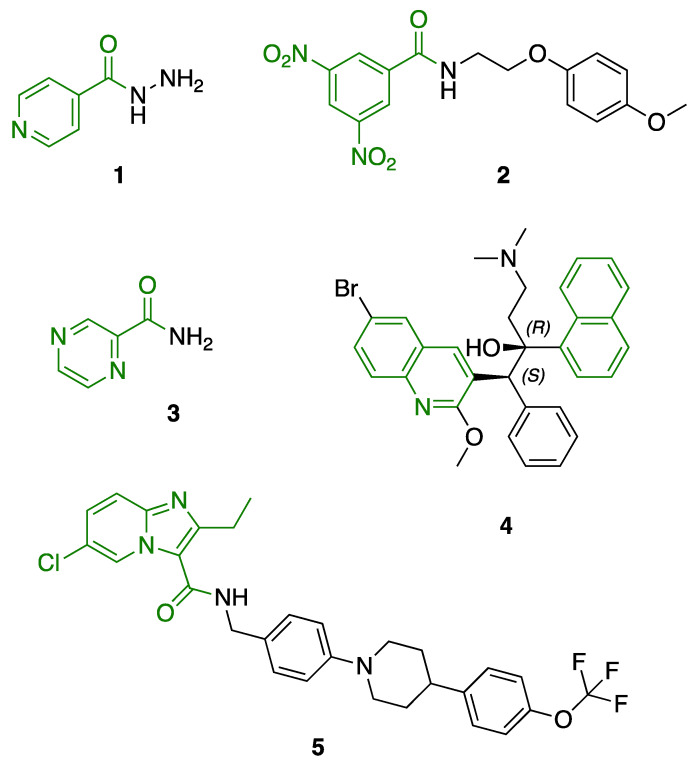
Scaffold hopping architectures (green) of molecules with known anti-tuberculosis (TB) activity evaluated within this study.

Previously, we established the importance of the amino acid and hydrazide moieties within benzoxa-[2,1,3]-diazole-substituted amino acid hydrazides for selective therapeutic activity against *Mtb* [16]. The current study expanded upon this previous work and evaluated the impact of specific alteration of the benzoxa-[2,1,3]-diazole moiety towards the development of therapeutics with improved anti-*Mtb* activity, particularly towards drug-resistant *Mtb*. To achieve this objective, we adopted the scaffold hopping approach of known pharmacophores to identify putative molecules [17,18,19]. This predominantly focused on the utilisation of pharmacophores with known anti-*Mtb* activity amenable to incorporation at the site of the current benzoxa-[2,1,3]-diazole moiety. We specifically evaluated pharmacophores from drugs targeting the mycobacterial cell wall, isoniazid (INH) **1** and the 3,5-dinitrobenzene moiety of DNB-1, **2**; the multi-target drug Pyrazinamide **3**; the new generation of molecules perturbing the mycobacterial ETC, the quinoline and naphthalene of Bedaquiline **4** and the imidazo [1,2-a]pyridine-3-carboxy moiety of Q203 **5** (Figure 3).

Both the amino acid and hydrazide moieties were locked within the structure, utilising 4-trifluoromethylphenyl **6**–**7** and 3-chlorophenyl hydrazine **8**–**9**. In terms of the amino acids, we previously demonstrated alanine (Ala) to exhibit the highest activity when conjugated within these hydrazides [16]. To recapitulate the sulphur moiety present within several known anti-TB drugs [20], amino acids with this functionality were previously evaluated within the hydrazides. As cysteine was nucleophilic and not amenable to the introduction, methionine (Met) was utilised. Linkage of Met to either the 4-trifluoromethylphenyl or the 3-chlorophenyl hydrazine produced comparable therapeutic activity against *Mtb* and a therapeutic index similar to that of **6**, containing Ala. The choice of hydrazides was based on the fact that 4-trifluoromethylphenyl hydrazide **6** had exhibited moderate activity against *Mtb* and a significant therapeutic index relative to cytotoxicity against mammalian cells, and 3-chlorophenyl hydrazine **8** had demonstrated notable impact upon *Mtb* therapeutic activity [16].

The objective of this study was to evaluate the importance of modifying the benzoxa-[2,1,3]-diazole moiety scaffold with those from other molecules with known anti-TB activity in order to appraise the therapeutic impact of substituents at this position and the potential for exploitation towards the development of molecules with demonstrated activity against drug-resistant *Mtb*.

## 2. Results and Discussion

### 2.1. Chemical Synthesis of Substituted Amino Acid Hydrazides

**Scheme 1:**
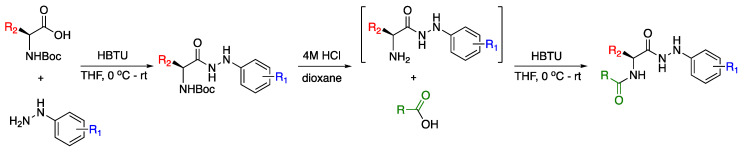
Synthesis of amino acid hydrazides and the desired substituted compounds.

Previously established methods were employed to synthesise the substituted amino acid hydrazides starting from *N*-Boc amino acids and the corresponding hydrazide (Scheme 1).

Coupling of the mono-substituted hydrazine with the *N*-Boc amino acids was achieved, utilising standard peptide coupling reagents, in high yields to provide sufficient material to then allow coupling with the corresponding aryl carboxylic acids via the same procedure.

### 2.2. Structure-Activity Relationship of Therapeutic Activity against Mtb

Following the synthetic approach, the substituted amino acid hydrazides **6**–**37** were screened against drug-susceptible, INH or RIF mono-resistant strains of *Mtb* in a standard resazurin microtitre assay (REMA) [16,21]. Considering the entire results, all of the compounds broadly inhibited the growth of drug-susceptible and the mono-drug-resistant *Mtb* strains. In comparison with our previous studies using benzoxa-[2,1,3]-diazoles, we observed a level of antibacterial activity in the wild-type strain similar to or better than our lead compounds (Figure S1, Supporting Information). Notably, there was a greater antibacterial activity observed within the INH mono-resistant strain, implicating scope with this approach for the development of drugs with activity against drug-resistant *Mtb*.

No discernible difference in antibacterial activity with Boc-protected compounds was observed between the 4-trifluoromethylphenyl alanine (Ala) hydrazide **6** or 4-trifluoromethylphenyl methionine (Met) hydrazide **7**. However, encouragingly, the Boc-protected 3-chlorophenyl Ala and 3-chlorophenyl Met hydrazide **8** and **9** demonstrated an observable increase in activity relative to the 4-trifluorophenyl amino acid hydrazides **6**–**7** (Table 1).

Substitution of the Boc with aryl heterocycles that mimic INH and pyrazinamide, **10**–**17**, in most cases, did not provide enhanced antibacterial activity (Table 1). Albeit this substitution in one case actually afforded no therapeutic activity **14**. However, differential selectivity was noted, with excellent activity against INH-resistant *Mtb* exhibited with the 3-chlorophenyl Ala and Met hydrazide of pyridine **12**–**13** and pyrazine **16**–**17** (Table 1). This notable observation is promising towards the development of therapies for drug-resistant *Mtb*, with significant potential that these compounds may employ a novel mechanism of action involving the circumventing of INH-activating enzyme, KatG, as the INH-resistant *Mtb* strain used in this study harboured an inactivating (Ser315 → Asp) in this protein, which was generated and identified in this study, analogous to previously published laboratory-generated and clinical strains [22].

An important criterion for any putative anti-TB drug is a demonstration of a clear therapeutic index between the concentration required to induce antibacterial activity and that which induces toxicity against the host system (i.e., macrophages and human tissue*S*). In this context, we set an arbitrary index of at least a 10-fold differential between the two outcomes. In the context of wild-type *Mtb* and RIF-resistant *Mtb*, none of the molecules demonstrated any discernible differential between antibacterial activity and mammalian cell cytotoxicity. Interestingly and in contrast, several molecules demonstrated a >10-fold therapeutic index when evaluated against INH-resistant *Mtb*. The Boc-protected 3-chlorophenyl Ala and Met hydrazides **8** and **9** gave excellent profiles, with a minimum inhibitory concentration (MIC) against INH-resistant *Mtb* of approximately 3 µM and less than 10% toxicity against macrophages at 100 µM, indicating a 30–40-fold therapeutic index. Although the pyrazine 3-chlorophenyl Ala and Met hydrazides **16** and **17** also exhibited selectivity toward INH-resistant *Mtb*, they demonstrated significant cytotoxicity against macrophages, implicating detrimental effects of the pyrimidine scaffold upon mammalian cells. The reasons for this are currently unclear.

The exploitation of the bedaquiline scaffolds, utilising the naphthalene and quinoline moieties (substituted at the 1-, 2- or 3- position with a carboxy group) **18**–**29**, was evaluated as a strategy. Relative to the mono-substituted aryl heterocycles, this scaffold did not produce improved activity suggestive of further evaluation. Incorporation of the 2-substituted naphthalene moiety **18**–**21** produced a somewhat moderate, yet still disappointing, enhancement in anti-TB activity over 1- substituted naphthalene **22**–**25.** Correspondingly, 3-substituted quinoline derivatives **26**–**29** displayed similar activity to the 2-naphthalene derivatives **18**–**21** with only the 4-trifluoromethylphenyl Met hydrazide **19** and 3-chlorophenyl Ala hydrazide **29** exhibiting anti-TB activity less than 50 µM. In support of these findings, a similar series of N-(alkoxyphenyl)-2-hydroxynaphthalene-1-carboxamides and their positional isomers N-(alkoxyphenyl)-1-hydroxynaphthalene-2-carboxamides have presented varied in vitro anti-TB activity (representative 12 μM and 97 μM, respectively) [23]. Further to demonstrating the poor antibacterial activity, the inclusion of the bedaquiline scaffold also proved to be detrimental to mammalian cells, supporting the cessation of this scaffold as an approach. The only notable outcome from this series was the putative selectivity of 3-chlorophenyl Ala hydrazide **29** against INH-resistant *Mtb*, with a MIC of 9 µM versus 37 µM against wild-type and RIF-resistant *Mtb* strains, albeit toxic against mammalian cells. This putative selectivity might be a consequence of bedaquiline inhibiting mycobacterial ATP synthase and counteracting the perturbation of the mycobacterial electron transport chain afforded by resistance to INH, as implicated in a recent study [24]. Further evaluation of this concept is, therefore, warranted.

Incorporation of 3,5-dinitrophenyl **30**–**33** resulted in viable antibacterial activity, with MIC values often <50 µM across all strains of *Mtb*. Interestingly, differential selectivity towards drug-resistant strains was observed within this series. The 3-chlorophenyl Ala hydrazide of 3,5-dinitrophenyl **32** showed similar activity against wild-type and RIF-resistant *Mtb*, with a 4-fold greater activity against INH-resistant *Mtb*. However, the 3-chlorophenyl Met hydrazide of 3,5-dinitrophenyl **33** showed increased activity relative to the Ala hydrazide against wild-type *Mtb* and a two-fold selectivity toward INH-resistant *Mtb* but surprisingly showed much greater activity against RIF-resistant *Mtb* with 4-fold selectivity over that of wild-type *Mtb*. This was the only compound in this series, demonstrating differential activity against RIF-resistant compared to wild-type *Mtb*, the mechanism of which is as yet unexplored but worthy of further investigations. Mechanistically, it is highly likely that these compounds **30**–**33** induce a general nitric oxide stress response similar to that observed with the known anti-TB compounds—metronidazole, delamanid and pretomanid—involving metabolic activation by enzymes, such as deazaflavin-dependent nitroreductase or the pyruvate-ferredoxin oxidereductase system, based on the fact they include nitro containing groups and functionality [25,26]. Such a mode of action involving oxidative stress would explain the fact that this series was highly toxic against mammalian cells. However, unlike metronidazole, delamanid and pretomanid, the activation mechanism must involve an enzymatic pathway common to both bacteria and mammalian cells.

The Q203 analogues **34**–**37** were of particular interest with respect to the development of molecules, exploiting new therapeutic pathways and bypassing conventional drug resistance mechanisms, for the treatment of drug-resistant *Mtb*. The imidazo[1,2-a]pyridine-3-carboxy moiety has been identified as the active component of several novel anti-tubercular drugs, including the pantothenate synthetase inhibitor GSK358607A and Q203 **1,** which targets the cytochrome *bcc-aa3* supercomplex [27,28,29]. Although the 4-trifluoromethylphenyl imidazo[1,2-a]pyridine-3-carboxy-substituted hydrazides **34**–**35** demonstrated marginally better activity than their 3-chlorophenyl counterparts **36**–**37**, no selectivity against drug-resistant strains of *Mtb* was observed with these compounds. With the 3-chlorophenyl imidazo[1,2-a]pyridine-3-carboxy-substituted hydrazides **36**–**37**, the inclusion of Met over Ala resulted in a two-fold increase in antibacterial activity. In contrast to the 4-trifluoromethylphenyl-substituted hydrazides, the 3-chlorophenyl-substituted hydrazides showed exquisite selectivity toward INH-resistant *Mtb* with MIC values <10 µM for both the Ala **36** and Met **37** containing compounds. However, although showing potent antibacterial activity, all of the imidazo[1,2-a]pyridine-3-carboxy-substituted hydrazides exhibited significant toxicity against macrophages, implying their limited utility as viable drug molecules. Despite this, a greater understanding of the structure-activity relationship (SAR) and identification of the targets of these compounds is an important concept in improving our understanding of principles associated with their selectivity towards drug-resistant *Mtb*.

With respect to the imidazo[1,2-a]pyridine-3-carboxy-derived hydrazides **34**–**37**, it was conceivable that the target of these compounds could include either the cytochrome supercomplex of proteins, as is the case with Q203 **1**, or pantothenate synthetase, the reported target of GSK358607A. Nevertheless, our results implied that the target was unlikely to be pantothenate synthetase since the *Mtb* mc^2^7000 strains used in this study possessed Δ*panCD* gene deletions and relied on media supplementation with pantothenate for survival and growth, thereby abrogating dependency for growth upon this enzyme. Inclusion of the imidazo[1,2-a]pyridine-3-carboxy moiety resulted in equivalent or greater activity than the initial Boc-protected compounds **6**–**9**. To further confirm this hypothesis, the activity of compounds **34**–**37** was evaluated against *Mycobacterium bovis* BCG as this constitutively expressed the pantothenate pathway. No difference in activity was detected with these compounds when in the presence or absence of pantothenate in the growth media with this mycobacterium, reinforcing pantothenate synthetase as unlikely to be the therapeutic target for these compounds. Furthermore, this target would also not explain the selectivity of these compounds for INH-resistant *Mtb*.

A more likely mechanism and one which provides a rationale for the increased selectivity of the 3-chlorophenyl imidazo[1,2-a]pyridine-3-carboxy-substituted hydrazides **36**–**37** towards INH-resistant *Mtb* is one analogous to that of Q203. This drug targets the cytochrome *bcc-aa3* supercomplex and disrupts the ETC in mycobacteria [30]. Recently, in addition to inhibiting mycolic acid synthesis, the bactericidal activity of INH has also been linked to ATP production, increased oxygen consumption and subsequently perturbation of the mycobacterial ETC [24]. In this context, defects in NADH dehydrogenase and concomitantly ETC have been shown to confer resistance to INH in mycobacteria [31]. Further studies are thereby warranted to evaluate the involvement of this pathway in terms of conferring therapeutic selectivity of 3-chlorophenyl imidazo[1,2-a]pyridine-3-carboxy-substituted hydrazides towards INH-resistant *Mtb*. In addition, SAR studies to address the selectivity of the 3-chlorophenyl- but not the 4-trifluoromethylphenyl-substituted hydrazides are also important.

## 3. Materials and Methods

All reactions were carried out under a nitrogen atmosphere in glassware dried in the oven overnight (>75 °C) or under high vacuum by a heat-gun unless otherwise stated. Ether refers to diethyl ether. Solvents were obtained dry from Sigma Aldrich, Gillingham, UK or another appropriate chemical supplier and used as supplied. In cases where mixtures of solvents were utilised, the ratios refer to the volumes used. Reagents were used as supplied unless otherwise stated. Flash chromatography was carried out using silica gel 40–63 μ 60Å. Analytical thin layer chromatography (TLC) was performed using precoated aluminium or glass-backed plates (silica gel 60Å F_254_) and visualised by UV radiation at 254 nm, or by staining with phosphomolybdic acid in ethanol or potassium permanganate in water. All melting points were determined using a Gallenkamp melting point apparatus and are uncorrected. Infrared spectra were recorded using a Diamond ATR (attenuated total reflection) accessory (Golden Gate) or as a solution in chloroform via transmission IR cells on a Bruker Tension 5 spectrometer. ^1^H NMR spectra were recorded in deutero solvent on Bruker Avance III 300, Bruker Avance II 400, Bruker Avance III HD 500 or Bruker Avance III HD 700 instruments and are reported as follows; chemical shift δ (ppm) (number of protons, multiplicity, coupling constant *J* (Hz), assignment). Residual protic solvent CHCl_3_ (δ_H_ = 7.26) or DMSO (δ_H_ = 2.50) were used as the internal reference. ^13^C NMR spectra were recorded using the central resonance of CDCl_3_ (δ_C_ = 77.0 ppm) or DMSO (δ_C_ = 39.5 ppm) as the internal reference. All chemical shifts are quoted in parts per million relative to tetramethylsilane (δ_H_ = 0.00 ppm) and coupling constants are given in Hertz to the nearest 1 Hz. Assignment of spectra were carried out using COSY, HSQC, HMBC and NOESY experiments and all ^1^H and ^13^C NMR for new compounds are provided for reference (Figure S2, Supporting Information). Electrospray mass spectra (E*S*) were obtained on a Micromass LCT mass spectrometer. High resolution mass spectra (HRM*S*) were obtained using a Thermo LTQ mass spectrometer (E*S*).

### 3.1. Chemistry

#### 3.1.1. Standard Procedure A: Synthesis of Boc Amino Acid Hydrazides*

A solution of *N*-Boc amino acid (1.0 g, 5.5 mmol) in dichloromethane (DCM) (25 mL) was treated with *N,N*-Diisopropylethylamine (DIPEA) (1.2 equiv.) and *N*-aryl hydrazine (1.2 equiv.), followed by 4-(4,6-dimethoxy-1,3,5-triazin-2-yl)-4-methyl-morpholinium chloride (DMTMM) (1.2 equiv.), and the solution was stirred at room temperature for 3 h. The reaction was mixed with DCM (20 mL), and the organic layer was washed with sat. aq. NH_4_Cl (20 mL), sat. aq. NaHCO_3_ (20 mL) and brine (20 mL). The organic layer was dried over MgSO_4_, filtered, evaporated and dried in vacuo to afford the desired *N*-Boc amino acid hydrazides.

*Compound **6** synthesis is previously reported [16].

#### 3.1.2. Standard Procedure B: Creation of New Amino Acid Hydrazides

##### Stage 1

The corresponding *N*-Boc amino acid hydrazides (0.10 g, 0.29 mmol) were mixed with 4 M HCl in dioxane (3 mL) and stirred at room temperature for 1 h under nitrogen. The solvent was then removed in vacuo, and the resultant oil was dissolved in DCM (5 mL) and evaporated three times to afford the desired deprotected amino acid hydrazide, which was used directly in stage 2 without further purification.

##### Stage 2

Crude deprotected amino acid hydrazide (0.29 mmol) was dissolved in DCM (5 mL) and treated successively with the aryl carboxylic acid (1.3 equiv.) and DIPEA (2.0 equiv.), followed by DMTMM (1.2 equiv.) at room temperature under nitrogen. The reaction was stirred for 3 h, then diluted with DCM (15 mL). The organic layer was washed with sat. aq. NH_4_Cl (20 mL), sat. aq. NaHCO_3_ (20 mL) and brine (20 mL). The organic layer was dried over MgSO_4_, filtered, evaporated and dried in vacuo. The resultant solid was triturated with CHCl_3_ to afford the desired *N*-substituted amino acid hydrazide.

#### 3.1.3. Boc-Protected Compounds

*tert-*butyl *N-*[(1*S*)-3-(methylsulfanyl)-1-{*N′*-[4-(trifluoromethyl)phenyl]hydrazine carbonyl}propyl]carbamate, **7**, following the standard procedure A, Boc-Met-OH (0.2 g, 0.8 mmol) and 4-trifluoromethylphenylhydrazine were transformed following flash chromatography (DCM/EtOH/ sat. aq. NH_3_ [400:8:1], [200:8:1] and [100:8:1]) to afford the title compound as a pale yellow solid (0.14 g, 44%); R_f_ 0.32 (DCM/EtOH/ sat. aq. NH_3_ [200:8:1]); m.p. 102–108 °C; *ν*_max_ (ATR) 3321, 2984, 1680, 1662, 1515, 1327, 1306, 1156, 1102, 830, 589 cm^−1^; δ_H_ (700 MHz, CDCl_3_) 8.82 (1H, s, CONHNH), 7.41 (2H, d, *J* 8, Ar–H), 6.81 (2H, d*, J* 8, Ar–H), 6.53 (1H, s, CONHNH), 5.43 (1H, d, *J* 8, NHCHCO), 4.44 (1H, q*, J* 7, NHCHCO), 2.56 (2H, t, *J* 7, NHCH(CH_2_CH_2_SCH_3_)), 2.09–2.14 (5H, m, NHCH(CH_2_CH_2_SCH_3_), NHCH(CH_2_CH_2_SCH_3_)), 1.49 (9H, s, (C(CH_3_)_3_); δ_C_ (700 MHz, CDCl_3_) 172.4 (CHCONH), 156.1 (NHCOOC(CH_3_)_3_), 150.5 (Ar–C), 126.3 (Ar–C), 124.2 (1C, q, ^1^*J*_C–F_ 270, *C*F_3_), 122.7 (1C, q, ^2^*J*_C–F_ 32, *ipso*-Ar-CCF_3_), 112.7 (Ar–C), 80.9 (NHCOO-*C*), 51.9 (NHCHCO), 30.9 (NHCH(CH_2_CH_2_SCH_3_)), 30.24 (NHCH(CH_2_CH_2_SCH_3_)), 28.3 (NHCOOC(CH_3_)_3_), 15.4 (NHCH(CH_2_CH_2_S*C*H_3_)); *m*/*z* (ES^+^) 408 (MH^+^); HRMS (ES^+^) found MH^+^, 408.1573 (C_17_H_25_F_3_N_3_O_3_S requires 408.1569).

*tert-*butyl (*S*)-(1-(2-(3-chlorophenyl)hydrazineyl)-1-oxopropan-2-yl)carbamate, **8**, following the standard procedure A, Boc-Ala-OH (1.0 g, 5.3 mmol) and 3-chlorophenylhydrazine were transformed to afford the title compound as a deep-yellow solid (1.6 g, 97%); R_f_ 0.46 (DCM/EtOH/ sat. aq. NH_3_ [200:8:1]); m.p. 110–114 °C; *ν*_max_ (ATR) 3428, 3278, 3078, 2979, 2933, 1678, 1504, 1367, 1158 cm^−1^; δ_H_ (400 MHz, CDCl_3_) 8.44 (1H, bs, CONHNH), 7.03 (1H, t, *J* 8, Ar–H), 6.76 (1H, d, *J* 8, Ar-CH), 6.71 (1H, t, *J* 2, Ar-CH), 6.60 (1H, d, *J* 8, Ar-CH), 5.07 (1H, bs, CONHCH), 4.22–4.19 (1H, m, CH), 1.39 (9*H*, s, (C(CH_3_)_3_)), 1.31 (3H, d, *J* 7, NHCHCH_3_C=O); δ_C_ (400 MHz, CDCl_3_) 192.7 (O*C*=ONH), 173.0 (CH*C*=ONHNH), 155.9 ((CH_3_)_3_COC=O), 149.1 (Ar–C), 135.0 (Ar–C), 130.2 (Ar-CH), 121.0 (Ar–C), 113.4 (Ar-CH), 111.69 (Ar-CH), 48.7 (NHCHC=O), 28.3 (C(CH_3_)_3_), 17.5 (NHCH(CH_3_)CO); *m*/*z* (ES^+^) 314 ([^35^Cl]MH^+^), 316 ([^37^Cl]MH^+^); HRMS (ES^+^) found ([^35^Cl]MH^+^), 314.1271 (C_14_H_21_N_3_O_3_^35^Cl requires 314.1278).

*tert-*Butyl *N-*[(1*S*)-1-[*N′*-(3-chlorophenyl)hydrazinecarbonyl]-3-(methylsulfanyl)propyl] carbamate, **9**, following the standard procedure A, Boc-Met-OH (0.2 g, 0.802 mmol) and 3-chlorophenylhydrazine were transformed following flash chromatography (DCM/EtOH/ sat. aq. NH_3_ [400:8:1] and [200:8:1]) to afford the title compound as a pale yellow solid (0.16 g, 53%); R_f_ 0.30 (DCM/EtOH/ sat. aq. NH_3_ [200:8:1]); m.p. 94–97 °C; *ν*_max_ (ATR) 3331, 3272, 2985, 1683, 1658, 1521, 1163, 768 cm^−1^; δ_H_ (700 MHz, CDCl_3_) 8.73 (1H, s, CONHNH), 7.10 (1H, t, *J* 8, Ar–H), 6.85 (1H, d, *J* 8, Ar–H), 6.80 (1H, s, Ar–H), 6.67 (1H, d, *J* 8, Ar–H), 6.34 (1H, bs, CONHNH), 5.42 (1H, d, *J* 8, NHCHCO), 4.43 (1H, q, *J* 7, NHCHCO), 2.57 (2H, t, *J* 7, NHCHCH_2_CH_2_SCH_3_), 2.09–2.13 (5H, m, NHCHCH_2_CH_2_SCH_3_, NHCHCH_2_CH_2_SCH_3_), 1.47 (9H, s, C(CH_3_)_3_); δ_C_ (700 MHz, CDCl_3_) 172.2 (CHCONH), 156.9 (NHCOO(CH_3_)_3_), 149.0 (Ar–C), 134.9 (Ar–C), 130.2 (Ar–C), 121.0 (Ar–C), 113.4 (Ar–C), 111.6 (Ar–C), 80.8 (NHCOO*C*(CH_3_)_3_), 51.9 (NHCHCO), 31.0 (NHCHCH_2_CH_2_SCH_3_), 30.2 (NHCHCH_2_CH_2_SCH_3_), 28.32 (NHCOOC(CH_3_)_3_), 15.5 (NHCHCH_2_CH_2_SCH_3_); *m*/*z* (ES^+^) 374 ([^35^Cl]MH^+^), 376 ([^37^Cl]MH^+^), 396 ([^35^Cl]MNa^+^), 398 ([^37^Cl]MNa^+^); HRMS (ES^+^) found ([^35^Cl]MNa^+^), 396.1141 (C_16_H_24_N_3_O_3_S^35^ClNa requires 396.1125).

#### 3.1.4. Final Compounds

(*S*)-*N-*(1-Oxo-1-(2-(4-(trifluoromethyl)phenyl)hydrazineyl)propan-2-yl)isonicotinamide, **10**, following the standard procedure B, *tert-*butyl (*S*)-(1-oxo-1-(2-(4-(trifluoromethyl) phenyl)hydrazineyl)propan-2-yl)carbamate (0.10 g, 0.30 mmol) and isonicotinic acid were transformed to afford the title compound as a pale-yellow solid (76 mg, 72%); R_f_ 0.15 (DCM/EtOH/ sat. aq. NH_3_ [200:8:1]); *ν*_max_ (ATR) 3261 (N–H), 3095, 2973 (C–H), 1638 (C=O), 1617, 1536, 1327 (C–F), 1158 (C–N), 1102, 1065 (C–F) cm^−1^; δ_H_ (400 MHz, DMSO-d_6_) 10.05 (1H, s, CONHNH), 8.98 (1H, d, *J* 7, PyCONH), 8.73 (2H, d, *J* 6, Py-*H*), 8.39 (1H, bs, CONHNH), 7.82 (2H, d, *J* 6, Py-*H*), 7.44 (2H, d, *J* 8, Ar–H), 6.83 (2H, d, *J* 8, Ar–H), 4.50 (1H, m, NHCH(CH_3_)CO), 1.44 (3H, d, *J* 7, NHCH(CH_3_)CO); δ_C_ (100 MHz, DMSO-d_6_) 172.6 (CONHNH), 165.4 (PyCONH), 152.9 (*ipso*-Ar–C), 150.6 (Py-*C*), 141.4 (*ipso*-Py-*C*), 126.6 (Ar–C), 124.4 (1C, q, ^1^*J*_C–F_ 270, *C*F_3_), 122.0 (Py-*C*), 118.5 (1C, q, ^2^*J*_C–F_ 31, *ipso*-Ar-CCF_3_), 111.9 (Ar–C), 48.7 (NHCH(CH_3_)CO), 18.0 (NHCH(CH_3_)CO); δ_F_ (282 MHz, DMSO-d_6_) -59.28 (3F, s, CF_3_); *m*/*z* (ES^+^) 353 (MH^+^); HRMS (ES^+^) found MH^+^, 353.1237 (C_16_H_16_F_3_N_4_O_2_ requires 353.1225).

(*S*)-*N-*(4-(methylthio)-1-oxo-1-(2-(4-(trifluoromethyl)phenyl)hydrazineyl)butan-2-yl)isonicotinamide, **11**, following the standard procedure B, *tert-*Butyl *N-*[(1*S*)-3-(methylsulfanyl)-1-{*N′*-[4-(trifluoromethyl)phenyl]hydrazine carbonyl}propyl]carbamate (0.14 g, 0.41 mmol) and isonicotinic acid were transformed to afford the title compound as a white solid (108 mg, 64%); m.p. 179–181 °C; *ν*_max_ (ATR) 3264, 1665, 1639, 1615, 1531, 1480, 1410, 1323, 1236, 1158, 1105, 1065, 1010, 878, 836, 757 cm^−1^; δ_H_ (300 MHz, DMSO-d_6_) 10.12 (1H, s, CONHNH), 8.98 (1H, d, *J* 7, PyCONH), 8.74 (2H, d, *J* 6, Py-*H*), 8.43 (1H, bs, CONHNH), 7.84 (2H, d, *J* 6, Py-*H*), 7.45 (2H, d, *J* 8, Ar–H), 6.82 (2H, d, *J* 8, Ar–H), 4.61 (1H, m, NHCH(CH_2_CH_2_SCH_3_)), 2.67–2.53 (2H, m, NHCH(CH_2_CH_2_SCH_3_)), 2.15–2.09 (5H, m, NHCH(CH_2_CH_2_SCH_3_); δ_C_ (75 MHz, DMSO-d_6_) 171.7 (CONHNH), 165.3 (PyCONH), 150.6 (Py-*C*), 126.6 (Ar–C), 122.1 (Py-*C*), 111.9 (Ar–C), 52.3 (NHCH(CH_2_CH_2_SCH_3_)), 31.3 (NHCH(CH_2_CH_2_SCH_3_)), 30.4 (NHCH(CH_2_CH_2_SCH_3_)), 15.1 (NHCH(CH_2_CH_2_SCH_3_)); δ_F_ (282 MHz, DMSO-d_6_) -59.28 (3F, s, CF_3_); *m*/*z* (ES^+^) 413 (MH^+^); HRMS (ES^+^) found MH^+^, 413.1261 (C_18_H_20_N_4_O_2_S requires 413.1259).

(*S*)-*N-*(1-(2-(3-chlorophenyl)hydrazineyl)-1-oxopropan-2-yl)isonicotinamide, **12**, following the standard procedure B, *tert-*butyl (*S*)-(1-(2-(3-chlorophenyl)hydrazineyl)-1-oxopropan-2-yl)carbamate (0.10 g, 0.32 mmol) and isonicotinic acid were transformed to afford the title compound as a white solid (60 mg, 59%); R_f_ 0.13 (DCM/EtOH/ sat. aq. NH_3_ [200:8:1]); *ν*_max_ (ATR) 3302, 3254 (N–H), 3035 (Ar–CH), 2983, 2930 (C–H), 1668, 1638 (C=O) 1599, 1537, 1511 (C=C) 1106 cm^−1^; δ_H_ (400 MHz, DMSO-d_6_) 9.97 (1H, s, CONHNH), 8.99 (1H, d, *J* 7, Py-CONH), 8.75 (2H, d, *J* 6, Py-*H*), 8.08 (1H, s, CONHNH), 7.84 (2H, d, *J* 6, Py-*H*), 7.14 (1H, t, *J* 8, Ar–H), 6.75–6.66 (3H, m, Ar–H), 4.53 (1H, m, NHCH(CH_3_)CO), 1.44 (3H, d, *J* 7, NHCH(CH_3_)CO); δ_C_ (100MHz, DMSO-d_6_) 172.6 (CONHNH), 165.4 (Py-CO), 151.3 (*ipso*-Ar–C), 150.6 (Py-*C*), 141.3 (*ipso*-Py-*C*), 133.9 (*ipso*-Ar–C), 130.5 (Ar–C), 122.0 (Py-*C*), 118.3 (Ar–C), 111.9 (Ar–C), 111.3 (Ar–C), 48.7 (NHCH(CH_3_)CO), 17.9 (NHCH(CH_3_)CO); *m*/*z* (ES^+^) 319 ([^37^Cl]MH^+^), 321 ([^35^Cl]MH^+^); HRMS (ES^+^) found [^35^Cl]MH^+^, 319.0962 (C_15_H_16_N_4_O_2_^35^Cl requires 319.0962).

(*S*)-*N-*(1-(2-(3-chlorophenyl)hydrazineyl)-4-(methylthio)-1-oxobutan-2-yl)isonicotinamide, **13**, following the standard procedure B, *tert-*Butyl *N-*[(1*S*)-1-[*N′*-(3-chlorophenyl)hydrazinecarbonyl]-3-(methylsulfanyl)propyl] carbamate (0.35 g, 1.13 mmol) and isonicotinic acid were transformed to afford the title compound as a brown solid (113 mg, 26%); m.p. 78–81 °C; *ν*_max_ (ATR) 3259, 2919, 1659, 1622, 1597, 0521, 1475, 1407, 1385, 1310, 1229, 1151, 1096, 1065, 992, 834 cm^−1^; δ_H_ (300 MHz, DMSO-d_6_) 10.03 (1H, s, CONHNH), 8.98 (1H, d, *J* 7, PyCONH), 8.75 (2H, d, *J* 6, Py-*H*), 8.09 (1H, bs, CONHNH), 7.84 (2H, d, *J* 6, Py-*H*), 7.15 (1H, t, *J* 8, Ar–H), 6.78–6.62 (3H, m, Ar–H), 4.60 (1H, m, NHCH(CH_2_CH_2_SCH_3_)), 2.67–2.53 (2H, m, NHCH(CH_2_CH_2_SCH_3_)), 2.09–1.98 (5H, m, NHCH(CH_2_CH_2_SCH_3_); δ_C_ (75 MHz, DMSO-d_6_) 171.6 (CONHNH), 165.8 (PyCONH), 151.3 (*ipso*-Ar–C), 150.6 (Py-*C*), 141.4 (*ipso*-Ar–C), 134.0 (*ipso*-Ar–C), 131.3 (*ipso*-Ar–C), 130.8 (Ar–C), 122.1 (Py-*C*), 118.3 (Ar–C), 111.9 (Ar–C), 52.3 (NHCH(CH_2_CH_2_SCH_3_)), 31.2 (NHCH(CH_2_CH_2_SCH_3_)), 30.4 (NHCH(CH_2_CH_2_SCH_3_)), 15.1 (NHCH(CH_2_CH_2_SCH_3_)); *m*/*z* (ES^+^) 379 ([^35^Cl]MH^+^), 381 ([^37^Cl]MH^+^); HRMS (ES^+^) found [^35^Cl]MH^+^, 379.0994 (C_17_H_20_N_4_O_2_S^35^Cl requires 379.0995).

(*S*)-*N-*(1-Oxo-1-(2-(4-(trifluoromethyl)phenyl)hydrazineyl)propan-2-yl)pyrazine-2-carboxamide, **14**, following the standard procedure B, *tert-*butyl (*S*)-(1-oxo-1-(2-(4-(trifluoromethyl) phenyl)hydrazineyl)propan-2-yl)carbamate (0.10 g, 0.30 mmol) and pyrazinoic acid were transformed following flash chromatography (*n*-hexane/EtOAc [4:1] to afford the title compound as a white solid (54 mg, 52%); R_f_ 0.19 (DCM/EtOH/ sat. aq. NH_3_ [200:8:1]); *ν*_max_ 3255 (N–H), 3098, 2981 (C–H), 1638 (C=O), 1617, 1534, 1326 (C–F), 1156 (C–N), 1101, 1065 (C–F) cm^−1^; δ_H_ (400 MHz, DMSO-d_6_) 10.10 (1H, bs, CONHNH), 9.22 (1H, s, Pyz-*H*), 8.91 (1H, m, Pyz-*H*), 8.87 (1H, d, *J* 8, PyzCONH), 8.77 (1H, d, *J* 2, Pyz-*H*), 8.43 (1H, bs, CONHNH), 7.48 (2H, d, *J* 8, Ar–H), 6.84 (2H, d, *J* 8, Ar–H), 4.60 (1H, m, NHCH(CH_3_)CO), 1.50 (3H, d, *J* 7, NHCH(CH_3_)CO); δ_C_ (100 MHz, DMSO-d_6_) 172.2 (CONHNH), 163.1 (PyzCO), 152.8 (*ipso*-Ar–C), 148.2 (Pyz-*C*), 144.9 (*ipso*-Pyz-*C*), 143.99 (Pyz-*C*), 143.85 (Pyz-*C*), 126.6 (Ar–C), 123.3 (1C, q, ^1^*J*_C–F_ 269, *C*F_3_), 118.5 (1C, q, ^2^*J*_C–F_ 32, *ipso*-Ar-CCF_3_), 111.9 (Ar–C), 47.9 (NHCH(CH_3_)CO), 18.7 (NHCH(CH_3_)CO); δ_F_ (282 MHz, DMSO-d_6_) -59.31 (3F, s, CF_3_); *m*/*z* (ES^+^) 354 (MH^+^); HRMS (ES^+^) found MH^+^, 354.1188 (C_15_H_15_F_3_N_5_O_2_ requires 354.1178).

(*S*)-*N-*(4-(methylthio)-1-oxo-1-(2-(4-(trifluoromethyl)phenyl)hydrazineyl)butan-2-yl)pyrazine-2-carboxamide, **15**, following the standard procedure B, *tert-*Butyl *N-*[(1*S*)-3-(methylsulfanyl)-1-{*N′*-[4-(trifluoromethyl)phenyl]hydrazine carbonyl}propyl]carbamate (78 mg, 0.23 mmol) and pyrazinoic acid were transformed to afford the title compound as a yellow semisolid (80 mg, 86%); *ν*_max_ (ATR) 3264, 1661, 1616, 1519, 1404, 1322, 1157, 1105, 1064, 1019, 838, 832, 771 cm^−1^; δ_H_ (300 MHz, CDCl_3_) 9.36 (1H, s, Pyz-*H*), 9.06 (1H, d, *J* 8, CONHNH), 8.78 (1H, d, *J* 2, Pyz-*H*), 8.53 (1H, bs, Pyz-*H*), 8.43 (1H, d, *J* 8, CONH), 7.40 (1H, d, *J* 8, Ar–H), 6.82 (1H, d, *J* 8, Ar–H), 4.99 (1H, m, NHCH(CH_2_CH_2_SCH_3_)), 2.63 (2H, m, NHCH(CH_2_CH_2_SCH_3_)), 2.35–2.10 (5H, m, NHCH(CH_2_CH_2_SCH_3_); δ_C_ (100 MHz, CDCl_3_) 171.2 (CONHNH), 163.7 (Pyz-CONH), 150.4 (*ipso*-Ar–C), 147.9 (Pyz-*C*), 145.0 (*ipso*-Pyz-*C*), 144.3 (Pyz-*C*), 143.4 (*ipso*-Ar–C), 142.8 (Pyz-*C*), 126.6 (Ar–C), 122.6 (1C, q, ^2^*J*_C–F_ 31, *ipso*-Ar-CCF_3_), 112.5 (Ar–C), 50.7 (NHCH(CH_2_CH_2_SCH_3_)), 30.94 (NHCH(CH_2_CH_2_SCH_3_)), 30.15 (NHCH(CH_2_CH_2_SCH_3_)), 15.4 (NHCH(CH_2_CH_2_SCH_3_)); *m*/*z* (ES^+^) 414 (MH^+^); HRMS (ES^+^) found MH^+^, 414.1207 (C^17^H^19^F^3^N^5^O^2^S requires 414.1212).

(*S*)-*N-*(1-(2-(3-chlorophenyl)hydrazineyl)-1-oxopropan-2-yl)pyrazine-2-carboxamide, **16**, following the standard procedure B, *tert-*butyl (*S*)-(1-(2-(3-chlorophenyl)hydrazineyl)-1-oxopropan-2-yl)carbamate (0.10 g, 0.32 mmol) and pyrazinoic acid were transformed to afford the title compound as a white solid (63 mg, 62%) as a mixture of rotamers [6:1]; R_f_ 0.21 (DCM/EtOH/ sat. aq. NH_3_ [200:8:1]); *ν*_max_ (ATR) 3383, 3293 (N–H), 3029 (Ar-CH), 2922, 2865 (C–H), 1681, 1659 (C=O) 1596, 1536, 1092, 1018, 886 cm^−1^; NMR data given for major rotamer δ_H_ (400 MHz, CDCl_3_) 9.35 (1H, s, Pyz-*H*), 8.89 (1H, s, CONHNH), 8.76 (1H, d, *J* 2, Pyz-*H*), 8.53 (1H, m, Pyz-*H*), 8.34 (1H, d, *J* 8, Pyz-CONH), 7.06 (1H, dd, *J* 8, Ar–H), 6.79 (1H, d, *J* 8, Ar–H), 6.76 (1H, s, Ar–H), 6.65 (1H, d, *J* 8, Ar–H), 4.85 (1H, m, NHCH(CH_3_)CO), 1.57 (3H, d, *J* 7, NHCH(CH_3_)CO); δ_C_ (100 MHz, CDCl_3_) 172.1 (CONHNH), 163.5 (Pyz-CONH), 149.0 (*ipso*-Ar–C), 147.8 (Pyz-C), 144.3 (Pyz-*C*), 143.6 (*ipso*-Pyz-*C*), 142.8 (Pyz-*C*), 135.0 (*ipso*-Ar–C), 130.2 (Ar–C), 121.1 (Ar–C), 113.4 (Ar–C), 111.7 (Ar–C), 47.58 (NHCH(CH_3_)CO), 17.7 (NHCH(CH_3_)CO); *m*/*z* (ES^+^) 320 ([^35^Cl]MH^+^), 322 ([^37^Cl]MH^+^); HRMS (ES^+^) found [^35^Cl]MH^+^, 320.0914 (C_14_H_15_N_5_O_2_^35^Cl requires 320.0914).

(*S*)-*N-*(1-(2-(3-chlorophenyl)hydrazineyl)-4-(methylthio)-1-oxobutan-2-yl)pyrazine-2-carboxamide, **17**, following the standard procedure B, *tert-*Butyl *N-*[(1*S*)-1-[*N′*-(3-chlorophenyl)hydrazinecarbonyl]-3-(methylsulfanyl)propyl] carbamate (70 mg, 0.23 mmol) and pyrazinoic acid were transformed to afford the title compound as a white solid (61 mg, 71%); m.p. 99–102 °C; *ν*_max_ (ATR) 3333, 3290, 2921, 1708, 1678, 1652, 1596, 1585, 1523, 1482, 1438, 1425, 1404, 1375, 1318, 1299, 1272, 1234, 1221, 1188, 1163, 1114, 1091, 1071, 1052, 1023, 991, 958, 946, 914, 864, 809 cm^−1^; δ_H_ (400 MHz, DMSO-d_6_) 10.03 (1H, bs, CONHNH), 9.22 (1H, s, Pyz-*H*), 9.00 (1H, d, *J* 8, CONH), (1H, s, CONHNH), 8.91 (1H, d, *J* 2, Pyz-*H*), 8.78 (1H, bs, Pyz-*H*), 8.10 (1H, bs, CONHNH), 7.16 (1H, dd, *J* 8, Ar–H), 6.74–6.62 (3H, m, Ar–H), 4.70 (1H, m, NHCH(CH_2_CH_2_SCH_3_)), 2.59–2.50 (2H, m, NHCH(CH_2_CH_2_SCH_3_)), 2.18–2.03 (5H, m, NHCH(CH_2_CH_2_SCH_3_); δ_C_ (75 MHz, DMSO-d_6_) 171.2 (CONHNH), 163.7 (Pyz-CONH), 151.3 (*ipso*-Ar–C), 148.1 (Pyz-*C*), 145.0 (*ipso*-Pyz-*C*), 144.1 (Pyz-*C*), 143.8 (Pyz-*C*), 134.0 (*ipso*-Ar–C), 130.8 (Ar–C), 118.4 (Ar–C), 111.9 (Ar–C), 111.3 (Ar–C), 51.7 (NHCH(CH_2_CH_2_SCH_3_)), 31.6 (NHCH(CH_2_CH_2_SCH_3_)), 30.2 (NHCH(CH_2_CH_2_SCH_3_)), 15.1 (NHCH(CH_2_CH_2_SCH_3_)); *m*/*z* (ES^+^) 380 ([^35^Cl]MH^+^), 382 ([^37^Cl]MH^+^); HRMS (ES^+^) found [^35^Cl]MH^+^, 380.0948 (C_16_H_19_^35^ClN_5_O_2_S requires 380.0948).

(*S*)-*N-*(1-Oxo-1-(2-(4-(trifluoromethyl)phenyl)hydrazineyl)propan-2-yl)-2-naphthamide, **18**, following the standard procedure B, *tert-*butyl (*S*)-(1-oxo-1-(2-(4-(trifluoromethyl) phenyl)hydrazineyl)propan-2-yl)carbamate (0.10 g, 0.29 mmol) and 2-napthoic acid were transformed to afford the title compound as a white solid (65 mg, 56%); R_f_ 0.24 (DCM/EtOH/ sat. aq. NH_3_ [200:8:1]); *ν*_max_ (ATR) 3329, 3264 (N–H), 3051, 2995, 2854, 1674, 1618 (C=O), 1525, 1325 (C–F), 1160 (C–N), 1095, 1065 (C–F), 834 (C–C) cm^−1^; δ_H_ (400 MHz, DMSO) 10.04 (1H, bs, CONHNH), 8.82 (1H, d, *J* 2, Np-CONH), 8.55 (1H, s, Np–*H*), 8.42 (1H, d, *J* 2, CONHNH), 8.08–7.97 (4H, m, Np–*H*), 7.66–7.58 (2H, m, Np–*H*), 7.47 (2H, d, *J* 8, Ar–H), 6.87 (2H, d, *J* 8, Ar–H), 4.60 (1H, m, NHCH(CH_3_)), 1.49 (3H, d, *J* 7, NHCH(CH_3_)); δ_C_ (176 MHz, DMSO-d_6_) 173.1 (CONHNH), 166.9 (NpCONH), 153.0 (*ipso*-Ar–C)*,* 134.7 (*ipso*-Ar–C), 132.5 (*ipso*-Ar–C), 131.7 (*ipso*-Ar–C), 129.3 (Np–*C*), 128.4 (Np–*C*), 128.4 (Np–*C*), 128.2 (Ar–C), 128.1 (Np–*C*), 127.2 (Np–*C*), 126.6 (Np–*C*), 125.0 (Np–*C*), 123.7 (1C, q, ^1^*J*_C–F_ 269, CF_3_), 118.6 (1C, q, ^2^*J*_C–F_ 31, *ipso*-Ar-CCF_3_), 112.0 (Ar–C), 48.7 (NHCH(CH_3_)), 18.2 (NHCH(CH_3_)); δ_F_ (282 MHz, DMSO-d_6_) -59.3 (3F, s, CF_3_); *m*/*z* (ES^+^) 402 (MH^+^); HRMS (ES^+^) found MH^+^, 402.1427 (C_21_H_19_F_3_N_3_O_2_ requires 402.1429).

(*S*)-*N-*(4-(methylthio)-1-oxo-1-(2-(4-(trifluoromethyl)phenyl)hydrazineyl)butan-2-yl)-2-naphthamide, **19**, following the standard procedure B, *tert-*Butyl *N-*[(1*S*)-3-(methylsulfanyl)-1-{*N′*-[4-(trifluoromethyl)phenyl]hydrazine carbonyl}propyl]carbamate (65 mg, 0.19 mmol) and 2-naphthoic acid were transformed to afford the title compound as a white solid (53 mg, 61%); m.p. 163–166°C; *ν*_max_ (ATR) 3264, 1666, 1635, 1615, 1520, 1503, 1432, 1325, 1232, 1159, 1103, 1065, 1011, 955, 897, 865, 838 cm^−1^; δ_H_ (300 MHz, DMSO-d_6_) 10.12 (1H, bs, CONHNH), 8.83 (1H, d, *J* 7, Np-CONH), 8.56 (1H, s, Np–*H*), 8.44 (1H, bs, CONHNH), 8.10–7.94 (4H, m, Np–*H*), 7.65–7.55 (2H, m, Np–*H*), 7.60 (2H, d, *J* 8, Ar–H), 6.87 (1H, d, *J* 8, Ar–H), 4.69 (1H, m, NHCH(CH_2_CH_2_SCH_3_)), 2.74–2.55 (2H, m, NHCH(CH_2_CH_2_SCH_3_)), 2.19–2.06 (5H, m, NHCH(CH_2_CH_2_SCH_3_); δ_C_ (75 MHz, DMSO-d_6_) 172.1 (CONHNH), 167.4 (CONH), 152.9 (*ipso*-Ar–C), 134.7 (*ipso*-Ar–C), 134.0 (*ipso*-Ar–C), 132.5 (*ipso*-Ar–C), 131.8 (*ipso*-Ar–C), 129.3 (Np–*C*), 128.4 (Np–*C*), 128.2 (Np–*C*), 128.1 (Np–*C*), 127.2 (Np–*C*), 126.6 (Ar–C), 125.0 (Np–*C*), 118.8 (1C, q, ^2^*J*_C–F_ 32, *ipso*-Ar-CCF_3_), 111.9 (Ar–C), 52.3 (NHCH(CH_2_CH_2_SCH_3_)), 31.5 (NHCH(CH_2_CH_2_SCH_3_)), 30.5 (NHCH(CH_2_CH_2_SCH_3_)), 15.2 (NHCH(CH_2_CH_2_SCH_3_)); δ_F_ (282 MHz, DMSO-d_6_) 59.26 (3F, s, CF_3_); *m*/*z* (ES^+^) 462 (MH^+^), 484 (MNa^+^); HRMS (ES^+^) found MH^+^, 462.1465 (C_23_H_23_F_3_N_3_O_2_S requires 462.1463).

(*S*)-*N-*(1-(2-(3-chlorophenyl)hydrazineyl)-1-oxopropan-2-yl)-2-naphthamide, **20**, following the standard procedure B, *tert-*butyl (*S*)-(1-(2-(3-chlorophenyl)hydrazineyl)-1-oxopropan-2-yl)carbamate (0.10 g, 0.32 mmol) and 2-napthoic acid were transformed to afford the title compound as a white solid (86 mg, 73%); R_f_ 0.32 (DCM/EtOH/ sat. aq. NH_3_ [200:8:1]); *ν*_max_ (ATR) 3331, 3236 (NH), 3062 (Ar-CH), 2983, 2926 (CH), 1634, 1626 (C=O),1599, 1536, 1536 (C=C), 1334 (C–N) cm^−1^; δ_H_ (400 MHz, DMSO-d_6_) 9.96 (1H, bs, CONHNH), 8.82 (1H, d, *J* 7, Np-CONH), 8.56 (1H, s, Np–*H*), 8.07–7.95 (4H, m, Np–*H*), 7.67–7.57 (2H, m, Np–*H*), 7.14 (1H, dd, *J* 8, Ar–H), 6.77 (1H, t, *J* 2, Ar–H), 6.73–6.69 (2H, m, Ar–H), 4.58 (1H, m, NHCH(CH_3_)CO), 1.47 (3H, d, *J* 7, NHCH(CH_3_)CO); δ_C_ (100 MHz, DMSO-d_6_) 173.0 (CHCONHNH), 166.9 (Np-CONH), 151.4 (Ar–C), 134.7 (Np–*C*), 134.0 (*ipso*-Ar–C), 132.5 (Np–*C*), 131.8 (Np–*C*), 130.8 (Ar–C), 129.3 (Np–*C*), 128.3 (Np–*C*), 128.2 (Np–*C*), 128.1 (Np–*C*), 127.2 (Np–*C*), 125.0 (Np–*C*), 118.2 (Ar–C), 111.9 (Ar–C), 111.3 (Ar–C), 48.7 (NHCH(CH_3_)CO), 18.1 (NHCH(CH_3_)CO); *m*/*z* (ES^+^) 368 (^[35^Cl]MH^+^), 370 (^[37^Cl]MH^+^); HRMS (ES^+^) found ([^35^Cl]MH^+^, 368.1170 (C_20_H_19_N_3_O_2_^35^Cl requires 368.1166).

(*S*)-*N-*(1-(2-(3-chlorophenyl)hydrazineyl)-4-(methylthio)-1-oxobutan-2-yl)-2-naphthamide, **21**, following the standard procedure B, *tert-*Butyl *N-*[(1*S*)-1-[*N′*-(3-chlorophenyl)hydrazinecarbonyl]-3-(methylsulfanyl)propyl] carbamate (70 mg, 0.23 mmol) and 2-naphthoic acid were transformed to afford the title compound as a white solid (18 mg, 19%); m.p. 205–208 °C; *ν*_max_ (ATR) 3224, 1636, 1625, 1597, 1527, 1494, 1452, 1440, 1335, 1303, 1268, 1231, 1201, 1076, 989, 955, 892, 863, 839 cm^−1^; δ_H_ (400 MHz, DMSO-d_6_) 10.04 (1H, bs, CONHNH), 8.85 (1H, d, *J* 7, Np-CONH), 8.56 (1H, s, Np–*H*), 8.11 (1H, bs, CONHNH), 8.10–7.97 (4H, m, Np–*H*), 7.68–7.54 (2H, m, Np–*H*), 7.15 (1H, dd, *J* 8, Ar–H), 6.77 (1H, t, *J* 2, Ar–H), 6.73–6.65 (2H, m, Ar–H), 4.67 (1H, m, NHCH(CH_2_CH_2_SCH_3_)), 2.68–2.56 (2H, m, NHCH(CH_2_CH_2_SCH_3_)), 2.12–2.06 (5H, m, NHCH(CH_2_CH_2_SCH_3_); δ_C_ (176 MHz, DMSO-d_6_) 172.0 (CHCONHNH), 167.4 (Np-CONH), 151.4 (Ar–C), 134.7 (Np–*C*), 134.0 (*ipso*-Ar–C), 132.5 (Np–*C*), 131.8 (Np–*C*), 130.8 (Ar–C), 129.3 (Np–*C*), 128.3 (Np–*C*), 128.2 (Np–*C*), 128.1 (Np–*C*), 127.7 (Np–*C*), 125.0 (Np–*C*), 118.3 (Ar–C), 111.9 (Ar–C), 111.3 (Ar–C), 52.3 (NHCH(CH_2_CH_2_SCH_3_)), 31.4 (NHCH(CH_2_CH_2_SCH_3_)), 30.5 (NHCH(CH_2_CH_2_SCH_3_)), 15.1 (NHCH(CH_2_CH_2_SCH_3_)); *m*/*z* (ES^+^) 428 ([^35^Cl]MH^+^), 430 ([^37^Cl]MH^+^); HRMS (ES^+^) found [^35^Cl]MH^+^, 428.1201 (C_22_H_23_^35^ClN_3_O_2_S requires 428.1200).

(*S*)-*N-*(1-Oxo-1-(2-(4-(trifluoromethyl)phenyl)hydrazineyl)propan-2-yl)-1-naphthamide, **22**, following the standard procedure B, *tert-*butyl (*S*)-(1-oxo-1-(2-(4-(trifluoromethyl) phenyl)hydrazineyl)propan-2-yl)carbamate (0.10 g, 0.29 mmol) and 1-napthoic acid were transformed to afford the title compound as a white solid (54 mg, 46%); R_f_ 0.24 (DCM/EtOH/ sat. aq. NH_3_ [200:8:1]; *ν*_max_ (ATR) 3315, 3242 (N–H), 3074 (C–H), 2999, 2865 (C–H), 1667, 1633 (C=O), 1617, 1536, 1324 (C–F), 1159 (C–N), 1095, 1065 (C–F), 828, 787, 776 (C–C) cm^−1^; δ_H_ (400 MHz, DMSO-d_6_) 10.10 (1H, bs, CONHNH), 8.83 (1H, d, *J* 7, NpCONH), 8.47 (1H, bs, CONHNH), 8.28 (1H, d, *J* 8, Np–*H*), 8.03 (1H, d, *J* 8, Np–*H*), 7.98 (1H, d, *J* 8, Np–*H*) 7.68 (1H, d, *J* 7, Np–*H*), 7.59–7.50 (3H, m, Np–*H*), 7.47 (2H, d, *J* 8, Ar–H), 6.91 (2H, d, *J* 8, Ar–H), 4.60 (1H, m, NHCH(CH_3_)CO), 1.44 (3H, d, *J* 7, NHCH(CH_3_)CO); δ_C_ (100 MHz, DMSO-d_6_) 173.1 (CONHNH), 167.3 (NpCONH), 153.0 (*ipso*-NHNHAr–C)*,* 134.1 (Np–*C*), 134.0 (Np–*C*), 133.9 (Np–*C*), 130.2 (Np–*C*), 128.6 (Np–*C*), 126.9 (Np–*C*), 126.7 (Np–*C*), 126.6 (Ar–C), 126.1 (Np–*C*), 125.9 (Np–*C*), 125.3 (Np–*C*), 118.4 (1C, q, ^2^*J*_C–F_ 32, *ipso*-Ar-CCF_3_), 112.0 (Ar–C), 48.5 (NHCH(CH_3_)CO), 18.0 (NHCH(CH_3_)CO); δ_F_ (282 MHz, DMSO-d_6_) −59.25 (3F, s, CF_3_); *m*/*z* (ES^+^) 402 (MH^+^); HRMS (ES^+^) found MH^+^, 402.1433 (C_21_H_19_F_3_N_3_O_2_ requires 402.1429).

(*S*)-*N-*(4-(methylthio)-1-oxo-1-(2-(4-(trifluoromethyl)phenyl)hydrazineyl)butan-2-yl)-1-naphthamide, **23**, following the standard procedure B, *tert-*Butyl *N-*[(1*S*)-3-(methylsulfanyl)-1-{*N′*-[4-(trifluoromethyl)phenyl]hydrazine carbonyl}propyl]carbamate (65 mg, 0.19 mmol) and 1-naphthoic acid were transformed to afford the title compound as a white solid (53 mg, 61%); m.p. 189–192 °C; *ν*_max_ (ATR) 3239, 1671, 1635, 1616, 1592, 1521, 1439, 1414, 1378, 1325, 1256, 1231, 1182, 1104, 1065, 1011, 966, 939, 886, 834 cm^−1^; δ_H_ (300 MHz, DMSO-d_6_) 10.18 (1H, bs, CONHNH), 8.86 (1H, d, *J* 7, NpCONH), 8.50 (1H, bs, CONHNH), 8.27 (1H, d, *J* 8, Np–*H*), 8.08–7.94 (2H, m, Np–*H*), 7.66–7.52 (3H, m, Np–*H*), 7.47 (2H, d, *J* 8, Ar–H), 6.92 (2H, d, *J* 8, Ar–H), 4.70 (1H, m, NHCH(CH_2_CH_2_SCH_3_)), 2.74–2.55 (2H, m, NHCH(CH_2_CH_2_SCH_3_)), 2.15–2.01 (5H, m, NHCH(CH_2_CH_2_SCH_3_); δ_C_ (75 MHz, DMSO-d_6_) 172.1 (CONHNH), 167.3 (NpCONH), 153.0 (*ipso*-Ar–C)*,* 134.7 (*ipso*-Ar–C), 133.6 (*ipso*-Ar–C), 130.4 (*ipso*-Ar–C), 130.3 (Np–*C*), 128.6 (Np–*C*), 127.0 (Np–*C*), 126.7 (Ar–C), 126.5 (*ipso*-Ar–C), 126.1 (Np–*C*), 126.0 (Np–*C*), 125.4 (Np–*C*), 125.4 (Np–*C*), 118.3 (1C, q, ^2^*J*_C–F_ 31, *ipso*-Ar-CCF_3_), 112.0 (Ar–C), 52.1 (NHCH(CH_2_CH_2_SCH_3_)), 31.4 (NHCH(CH_2_CH_2_SCH_3_)), 30.4 (NHCH(CH_2_CH_2_SCH_3_)), 15.1 (NHCH(CH_2_CH_2_SCH_3_)); δ_F_ (282 MHz, DMSO-d_6_) 59.27 (3F, s, CF_3_); *m*/*z* (ES^+^) 462 (MH^+^), 484 (MNa^+^); HRMS (ES^+^) found MH^+^, 462.1468 (C_23_H_23_F_3_N_3_O_2_S requires 462.1463).

(*S*)-*N-*(1-(2-(3-chlorophenyl)hydrazineyl)-1-oxopropan-2-yl)-1-naphthamide, **24**, following the standard procedure B, *tert-*butyl (*S*)-(1-(2-(3-chlorophenyl)hydrazineyl)-1-oxopropan-2-yl)carbamate (0.10 g, 0.32 mmol) and 1-napthoic acid were transformed to afford the title compound as a white solid (55 mg, 45%); *ν*_max_ (ATR) 3404, 3237 (NH), 3052 (Ar-CH), 2995, 2946 (CH), 1634 (C=O), 1595 (C=C), 1324 (C–N) cm^−1^; δ_H_ (400 MHz, DMSO-d_6_) 10.00 (1H, bs, CONHNH), 8.83 (1H, d, *J* 7, Np-CONH), 8.32 (1H, dd, *J* 8, 2, Np–*H*), 8.15 (1H, s, CONHNH), 8.03 (1H, d, *J* 8, Np–*H*), 7.98 (1H, dd, *J* 8, 2, Np–*H*), 7.68 (1H, d, *J* 8, Np–*H*), 7.60–7.50 (2H, m, Np–*H*), 7.16 (1H, dd, *J* 8, Ar–H), 6.83 (1H, bs, Ar–H), 6.76–6.71 (2H, m, Ar–H), 4.59 (1H, m, NHCH(CH_3_)CO), 1.42 (3H, d, *J* 7, NHCH(CH_3_)CO); δ_C_ (100 MHz, DMSO-d_6_) 173.1 (CONHNH), 169.2 (Np-CONH), 151.4 (Ar–C), 134.6 (Np–*C*), 134.0 (Ar–C), 133.5 (Np–*C*), 130.8 (Ar–C), 130.3 (Np–*C*), 128.5 (Np–*C*), 127.0 (Np–*C*), 126.6 (Np–*C*), 126.2 (Np–*C*), 126.0 (Np–*C*), 125.3 (Np–*C*), 118.3 (Ar–C), 111.9 (Ar–C), 111.3 (Ar–C), 48.4 (NHCH(CH_3_)CO), 18.0 (NHCH(CH_3_)CO); *m*/*z* (ES^+^) 368 (^[35^Cl]MH^+^), 370 (^[37^Cl]MH^+^); HRMS (ES^+^) found ([^35^Cl]MH^+^, 368.1170 (C_20_H_19_N_3_O_2_^35^Cl requires 368.1166).

(*S*)-*N-*(1-(2-(3-chlorophenyl)hydrazineyl)-4-(methylthio)-1-oxobutan-2-yl)-1-naphthamide, **25**, following the standard procedure B, *tert-*Butyl *N-*[(1*S*)-1-[*N′*-(3-chlorophenyl)hydrazinecarbonyl]-3-(methylsulfanyl)propyl] carbamate (70 mg, 0.23 mmol) and 1-naphthoic acid were transformed to afford the title compound as a pale yellow solid (54 mg, 56%); m.p. 183–186 °C; *ν*_max_ (ATR) 3239, 1667, 1635, 1594, 1524, 1471, 1435, 1313, 1255, 1216, 1074, 992, 885, 851, 765 cm^−1^; δ_H_ (300 MHz, DMSO-d_6_) 10.07 (1H, bs, CONHNH), 8.87 (1H, d, *J* 7, NpCONH), 8.27 (1H, d, *J* 8, Np–*H*), 8.15 (1H, bs, CONHNH), 8.05–7.93 (2H, m, Np–*H*), 7.69 (1H, m, Np–H), 7.63–7.50 (3H, m, Np–*H*), 7.17 (1H, t, *J* 6, Ar–H), 6.82 (1H, s, Ar–H), 6.76–6.70 (2H, m, Ar–H), 4.68 (1H, m, NHCH(CH_2_CH_2_SCH_3_)), 2.70–2.58 (2H, m, NHCH(CH_2_CH_2_SCH_3_)), 2.15–2.01 (5H, m, NHCH(CH_2_CH_2_SCH_3_); δ_C_ (75 MHz, DMSO-d_6_) 172.1 (CONHNH), 169.5 (NpCONH), 151.4 (*ipso*-Ar–C)*,* 134.6 (*ipso*-Ar–C), 134.0 (*ipso*-Ar–C), 130.4 (*ipso*-Ar–C), 130.3 (Np–*C*), 128.6 (Np–*C*), 127.1 (Np–*C*), 126.7 (Ar–C), 126.7 (*ipso*-Ar–C), 126.1 (Np–*C*), 125.4 (Np–*C*), 118.3 (*ipso*-Ar–C), 112.0 (Ar–C), 111.4 (Ar–C), 52.1 (NHCH(CH_2_CH_2_SCH_3_)), 31.4 (NHCH(CH_2_CH_2_SCH_3_)), 30.4 (NHCH(CH_2_CH_2_SCH_3_)), 15.1 (NHCH(CH_2_CH_2_SCH_3_)); *m*/*z* (ES^+^) 428 ([^35^Cl]MH^+^), 430 ([^37^Cl]MH^+^); HRMS (ES^+^) found [^35^Cl]MH^+^, 428.1192 (C_22_H_23_N_3_O_2_S^35^Cl requires 428.1200).

(*S*)-*N-*(1-Oxo-1-(2-(4-(trifluoromethyl)phenyl)hydrazineyl)propan-2-yl)quinoline-3-carboxamide, **26**, following the standard procedure B, *tert-*butyl (*S*)-(1-oxo-1-(2-(4-(trifluoromethyl) phenyl)hydrazineyl)propan-2-yl)carbamate (0.10 g, 0.30 mmol) and 3-quinolinecarboxylic acid were transformed to afford the title compound as a white solid (53 mg, 44%); R_f_ 0.15 (DCM/EtOH/ sat. aq. NH_3_ [200:8:1]); *ν*_max_ (ATR) 3259 (N–H), 2998, 2935 (C–H), 1637, 1617 (C=O), 1522, 1450, 1325, 1158, 1103 1065 (C–F) cm^−1^; δ_H_ (400 MHz, DMSO-d_6_) 10.09 (1H, bs, CONHNH), 9.34 (1H, d, *J* 2, Quin-*H*), 9.06 (1H, d, *J* 7, QuinCONH), 8.93 (1H, s, Quin-*H*), 8.43 (1H, s, CONHNH), 8.11 (2H, m, Quin-*H*), 7.89 (1H, dd, *J* 8, Quin-*H*), 7.71 (1H, dd, *J* 8, Quin-*H*), 7.47 (2H, d, *J* 8, Ar–H), 6.87 (2H, d, *J* 8, Ar–H), 4.63 (1H, quin, *J* 7, NHCH(CH_3_)CO), 1.50 (3H, d, *J* 7, NHCH(CH_3_)CO); δ_C_ (100 MHz, DMSO-d_6_) 172.8 (CONHNH), 165.6 (Quin-CO), 152.9 (*ipso*-Ar–C), 149.7 (Quin-*C*), 148.9 (*ipso*-Ar–C), 136.4 (Quin-*C*), 131.7 (Quin-*C*), 129.6 (*ipso*-Ar–C), 129.2 (Quin-*C*), 127.9 (Quin-*C*), 127.1 (*ipso*-Ar–C), 126.6 (*ipso*-Ar–C), 124.2 (1C, q, ^1^*J*_C–F_ 272, Ar-C*C*F_3_), 118.6 (1C, q, ^2^*J*_C–F_ 31, *ipso*-Ar-CCF_3_), 111.9 (Ar–C), 48.8 (NHCH(CH_3_)CO), 18.1 (NHCH(CH_3_)CO); δ_F_ (282 MHz, DMSO-d_6_) -59.28 (3F, s, CF_3_); *m*/*z* (ES^+^) 403 (MH^+^); HRMS (ES^+^) found MH^+^, 403.1367 (C_20_H_18_F_3_N_4_O_2_ requires 403.1382).

(*S*)-*N-*(4-(methylthio)-1-oxo-1-(2-(4-(trifluoromethyl)phenyl)hydrazineyl)butan-2-yl)quinoline-3-carboxamide, **27**, following the standard procedure B, *tert-*Butyl *N-*[(1*S*)-3-(methylsulfanyl)-1-{*N′*-[4-(trifluoromethyl)phenyl]hydrazine carbonyl}propyl]carbamate (65 mg, 0.19 mmol) and 3-quinolinecarboxylic acid were transformed to afford the title compound as a beige solid (57 mg, 65%); m.p. 138–141 °C; *ν*_max_ (ATR) 3258, 3001, 1636, 1616, 1538, 1496, 1418, 1328, 1231, 1185, 1158, 1098, 1066, 1009, 959, 917, 902, 865, 832 cm^−1^; δ_H_ (300 MHz, DMSO-d_6_) 10.15 (1H, bs, CONHNH), 9.34 (1H, d, *J* 2, Quin-*H*), 9.06 (1H, d, *J* 7, QuinCONH), 8.93 (1H, s, Quin-*H*), 8.45 (1H, s, CONHNH), 8.12 (2H, m, Quin-*H*), 7.88 (1H, dd, *J* 8, Quin-*H*), 7.71 (1H, dd, *J* 8, Quin-*H*), 7.47 (2H, d, *J* 8, Ar–H), 6.85 (2H, d, *J* 8, Ar–H), 4.70 (1H, m, NHCH(CH_2_CH_2_SCH_3_)), 2.75–2.56 (2H, m, NHCH(CH_2_CH_2_SCH_3_)), 2.20–2.05 (5H, m, NHCH(CH_2_CH_2_SCH_3_); δ_C_ (75 MHz, DMSO-d_6_) 171.9 (CONHNH), 166.0 (Quin-CO), 152.9 (*ipso*-Ar–C), 149.7 (Quin-*C*), 148.9 (*ipso*-Ar–C), 136.4 (Quin-*C*), 131.7 (Quin-*C*), 129.6 (*ipso*-Ar–C), 129.3 (Quin-*C*), 127.9 (Quin-*C*), 127.1 (*ipso*-Ar–C), 126.9 (Ar–C), 123.7 (1C, q, ^1^*J*_C–F_ 270, Ar-C*C*F_3_), 118.6 (1C, q, ^2^*J*_C–F_ 31, *ipso*-Ar-CCF_3_), 112.0 (Ar–C), 52.4 (NHCH(CH_2_CH_2_SCH_3_)), 31.4 (NHCH(CH_2_CH_2_SCH_3_)), 30.5 (NHCH(CH_2_CH_2_SCH_3_)), 15.1 (NHCH(CH_2_CH_2_SCH_3_)); δ_F_ (282 MHz, DMSO-d_6_) -59.27 (3F, s, CF_3_); *m*/*z* (ES^+^) 463 (MH^+^); HRMS (ES^+^) found MH^+^, 463.1416 (C_22_H_22_F_3_N_4_O_2_S requires 463.1416).

(*S*)-*N-*(1-(2-(3-chlorophenyl)hydrazineyl)-1-oxopropan-2-yl)quinoline-3-carboxamide, **28**, following the standard procedure B, *tert-*butyl (*S*)-(1-(2-(3-chlorophenyl)hydrazineyl)-1-oxopropan-2-yl)carbamate (0.10 g, 0.32 mmol) and 3-quinolinecarboxylic acid were transformed to afford the title compound as a white solid (37 mg, 30%); R_f_ 0.29 (DCM/EtOH/ sat. aq. NH_3_ [200:8:1]); *ν*_max_ (ATR) 3324 (N–H), 2988, 2947 (C–H), 1646, 1613 (C=O), 1513 (C=N), 1151, 1103, 1065 (C–F) cm^−1^; δ_H_ (400 MHz, DMSO-d_6_) 10.00 (1H, bs, CONHNH), 9.35 (1H, d, *J* 2, Quin-NCH), 9.08 (1H, d, *J* 7, Quin-CONH), 8.93 (1H, s, Quin-CH), 8.15–8.07 (3H, m, Quin-CH, CONHNH), 7.89 (1H, t, *J* 8, Quin-CH), 7.72 (1H, t, *J* 8, Quin-CH), 7.15 (1H, t, *J* 8, Ar–H), 6.78 (1H, t, *J* 2, Ar–H), 6.71 (2H, m, Ar–H), 4.60 (1H, m, NHCH(CH_3_)), 1.48 (3H, d, *J* 7, NHCH(CH_3_)); δ_C_ (75 MHz, DMSO-d_6_) 172.7 (CONHNH), 165.6 (Quin-CONH), 151.4 (*ipso*-Ar–C), 149.7 (Quin-*C*), 149.0 (*ipso*-Quin-*C*), 136.4 (Quin-*C*), 134.0 (*ipso*-Ar–C), 131.7 (Quin-*C*), 130.8 (Ar–C), 129.6 (Quin-*C*), 129.3 (Quin-*C*), 127.9 (Quin-*C*), 127.1 (*ipso*-Ar–C), 126.9 (*ipso*-Ar–C), 118.3 (Ar–C), 111.9 (Ar–C), 111.3 (Ar–C), 48.7 (NHCH(CH_3_)), 18.1 (NHCH(CH_3_)); *m*/*z* (ES^+^) 369 ([^35^Cl]MH^+^), 371 ([^37^Cl]MH^+^); HRMS (ES^+^) found [^35^Cl]MH^+^, 369.1120 (C_19_H_18_N_4_O_2_^35^Cl requires 369.1118).

(*S*)-*N-*(1-(2-(3-chlorophenyl)hydrazineyl)-4-(methylthio)-1-oxobutan-2-yl)quinoline-3-carboxamide, **29**, following the standard procedure B, *tert-*Butyl *N-*[(1*S*)-1-[*N′*-(3-chlorophenyl)hydrazinecarbonyl]-3-(methylsulfanyl)propyl] carbamate (70 mg, 0.23 mmol) and 3-quinolinecarboxylic acid were transformed to afford the title compound as a white solid (68 mg, 71%); m.p. 141–144 °C; *ν*_max_ (ATR) 3211, 3029, 2913, 1664, 1638, 1595, 1570, 1534, 1492, 1424, 1374, 1307, 1272, 1229, 1203, 1155, 1136, 1112, 1080, 1019, 993, 981, 960, 948, 914, 900, 876, 855 cm^−1^; δ_H_ (400 MHz, DMSO-d_6_) 10.08 (1H, bs, CONHNH), 9.35 (1H, d, *J* 2, Quin-NCH), 9.07 (1H, d, *J* 7, Quin-CONH), 8.93 (1H, s, Quin-CH), 8.15–8.07 (3H, m, Quin-CH, CONHNH), 7.89 (1H, t, *J* 8, Quin-CH), 7.72 (1H, t, *J* 8, Quin-CH), 7.15 (1H, t, *J* 8, Ar–H), 6.77 (1H, t, *J* 2, Ar–H), 6.70 (2H, m, Ar–H), 4.67 (1H, m, NHCH(CH_2_CH_2_SCH_3_)), 2.71–2.59 (2H, m, NHCH(CH_2_CH_2_SCH_3_)), 2.16–2.06 (5H, m, NHCH(CH_2_CH_2_SCH_3_); δ_C_ (75 MHz, DMSO-d_6_) 171.8 (CONHNH), 166.0 (Quin-CONH), 151.4 (*ipso*-Ar–C), 149.7 (Quin-*C*), 149.0 (*ipso*-Quin-*C*), 136.4 (Quin-*C*), 134.0 (*ipso*-Ar–C), 131.7 (Quin-*C*), 130.8 (Ar–C), 129.6 (Quin-*C*), 129.3 (Quin-*C*), 127.9 (Quin-*C*), 127.1 (*ipso*-Ar–C), 126.9 (*ipso*-Ar–C), 118.3 (Ar–C), 111.9 (Ar–C), 111.4 (Ar–C), 52.4 (NHCH(CH_2_CH_2_SCH_3_)), 31.4 (NHCH(CH_2_CH_2_SCH_3_)), 30.4 (NHCH(CH_2_CH_2_SCH_3_)), 15.1 (NHCH(CH_2_CH_2_SCH_3_)); *m*/*z* (ES^+^) 429 ([^35^Cl]MH^+^), 431 ([^37^Cl]MH^+^); HRMS (ES^+^) found [^35^Cl]MH^+^, 429.1148 (C_21_H_22_^35^ClN_4_O_2_S requires 429.1152).

(*S*)-3,5-Dinitro-*N-*(1-oxo-1-(2-(4-(trifluoromethyl)phenyl)hydrazineyl)propan-2-yl)benzamide, **30**, following the standard procedure B, *tert-*butyl (*S*)-(1-oxo-1-(2-(4-(trifluoromethyl) phenyl)hydrazineyl)propan-2-yl)carbamate (0.10 g, 0.30 mmol) and 3,5-dinitrobenzoic acid were transformed to afford the title compound as a white solid (79 mg, 60%); *ν*_max_ (ATR) 3250 (NH), 3101 (Ar-CH), 2896 (CH), 1641 (C=O), 1617 (Ar C–C), 1535, 1342, 1330, 1160, 1107, 1066 (C–F) cm^−1^; δ_H_ (400 MHz, DMSO-d_6_) 10.13 (1H, bs, CONHNH), 9.47 (1H, d, *J* 7, ArCONH), 9.14 (2H, d, *J* 2, Ar–H), 8.98 (1H, dd, *J* 2, Ar–H), 8.43 (1H, bs, CONHNH), 7.47 (2H, d, *J* 8, Ar–H), 6.84 (2H, d, *J* 8, Ar–H), 4.61 (1H, m, NHCH(CH_3_)CO), 1.49 (3H, d, *J* 7, NHCH(CH_3_)CO); δ_C_ (100 MHz, DMSO-d_6_) 172.4 (CONHNH), 163.0 (ArCONH), 152.9 (*ipso*-Ar–C), 148.6 (*ipso*-Ar–C), 137.1 (*ipso*-Ar–C), 128.4 (Ar–C), 126.7 (Ar–C), 121.5 (Ar–C), 111.9 (Ar–C), 49.2 (NHCH(CH_3_)CO), 18.0 (NHCH(CH_3_)CO); δ_F_ (282 MHz, DMSO-d_6_) -59.34 (3F, s, CF_3_); *m*/*z* (ES^+^) 442 (MH^+^); HRMS (ES^+^) found MH^+^, 442.0958 (C_17_H_15_F_3_N_5_O_6_ requires 442.0974).

(*S*)-*N-*(4-(methylthio)-1-oxo-1-(2-(4-(trifluoromethyl)phenyl)hydrazineyl)butan-2-yl)-3,5-dinitrobenzamide, **31**, following the standard procedure B, *tert-*Butyl *N-*[(1*S*)-3-(methylsulfanyl)-1-{*N′*-[4-(trifluoromethyl)phenyl]hydrazine carbonyl}propyl]carbamate (65 mg, 0.19 mmol) and 3,5-dinitrobenzoic acid were transformed to afford the title compound as a beige solid (36 mg, 38%); m.p. 97–100 °C; *ν*_max_ (ATR) 3464, 3212, 3125, 3075, 1662, 1633, 1614, 1541, 1440, 1342, 1322, 1251, 1162, 1115, 1063, 1010, 933, 837 cm^−1^; δ_H_ (300 MHz, DMSO-d_6_) 10.20 (1H, bs, CONHNH), 9.45 (1H, d, *J* 7, ArCONH), 9.15 (2H, d, *J* 2, Ar–H), 8.99 (1H, dd, *J* 2, Ar–H), 8.44 (1H, bs, CONHNH), 7.47 (2H, d, *J* 8, Ar–H), 6.84 (2H, d, *J* 8, Ar–H), 4.68 (1H, m, NHCH(CH_2_CH_2_SCH_3_)), 2.74–2.53 (2H, m, NHCH(CH_2_CH_2_SCH_3_)), 2.19–2.04 (5H, m, NHCH(CH_2_CH_2_SCH_3_); δ_C_ (75 MHz, DMSO-d_6_) 171.4 (CONHNH), 163.4 (ArCONH), 152.8 (*ipso*-Ar–C), 148.6 (*ipso*-Ar–C), 137.0 (*ipso*-Ar–C), 128.5 (Ar–C), 127.3 (*ipso*-Ar–C), 126.6 (Ar–C), 121.5 (Ar–C), 118.7 (1C, q, ^2^*J*_C–F_ 32, *ipso*-Ar-CCF_3_), 111.9 (Ar–C), 52.7 (NHCH(CH_2_CH_2_SCH_3_)), 31.2 (NHCH(CH_2_CH_2_SCH_3_)), 30.4 (NHCH(CH_2_CH_2_SCH_3_)), 15.1 (NHCH(CH_2_CH_2_SCH_3_)); δ_F_ (282 MHz, DMSO-d_6_) -59.30 (3F, s, CF_3_); *m*/*z* (ES^+^) 502 (MH^+^); HRMS (ES^+^) found MH^+^, 502.1010 (C_19_H_19_F_3_N_5_O_6_S requires 502.1008).

(*S*)-*N-*(1-(2-(3-chlorophenyl)hydrazineyl)-1-oxopropan-2-yl)-3,5-dinitrobenzamide, **32**, following the standard procedure B, *tert-*butyl (*S*)-(1-(2-(3-chlorophenyl)hydrazineyl)-1-oxopropan-2-yl)carbamate (0.10 g, 0.32 mmol) and 3,5-dinitrobenzoic acid were transformed to afford the title compound as a light brown solid (54 mg, 42%); R_f_ 0.21 (DCM/EtOH/ sat. aq. NH_3_ [200:8:1]); *ν*_max_ (ATR) 3264 (NH), 3094 (Ar-CH), 2983, 2926 (CH), 1651, 1597 (C=O), 1537, 1342 (N–O), 1334 (C=C) cm^−1^; δ_H_ (300 MHz, DMSO-d_6_) 10.05 (1H, bs, CONHNH), 9.48 (1H, d, *J* 7, ArCONH), 9.15 (2H, d, *J* 2, Ar–H), 8.99 (1H, m, Ar–H), 8.10 (1H, bs, CONHNH), 7.14 (1H, t, *J* 8, Ar–H), 6.76 (1H, t, *J* 2, Ar–H), 6.73–6.66 (2H, m, Ar–H), 4.57 (1H, m, NHCH(CH_3_)CO), 1.48 (3H, d, *J* 7, NHCH(CH_3_)CO); δ_C_ (100 MHz, DMSO-d_6_) 172.3 (CONHNH), 163.0 (ArCONH), 151.3 (*ipso*-Ar–C), 148.5 (*ipso*-Ar–C), 137.1 (ipso-Ar–C), 134.0 (*ipso*-Ar–C), 130.7 (Ar–C), 128.4 (Ar–C), 118.3 (Ar–C), 111.9 (Ar–C), 111.3 (Ar–C), 49.2 (NHCH(CH_3_)CO), 17.9 (NHCH(CH_3_)CO); *m*/*z* (ES^+^) 408 ([^35^Cl]MH^+^), 410 ([^37^Cl]MH^+^); HRMS (ES^+^) found [^35^Cl]MH^+^, 408.0701 (C_16_H_15_N_5_O_6_^35^Cl requires 408.0711).

(*S*)-*N-*(1-(2-(3-chlorophenyl)hydrazineyl)-4-(methylthio)-1-oxobutan-2-yl)-3,5-dinitrobenzamide, **33**, following the standard procedure B, *tert-*Butyl *N-*[(1*S*)-1-[*N′*-(3-chlorophenyl)hydrazinecarbonyl]-3-(methylsulfanyl)propyl] carbamate (100 mg, 0.32 mmol) and 3,5-dinitrobenzoic acid were transformed to afford the title compound as a light brown gum (149 mg, 99%); m.p. 87–89 °C; *ν*_max_ (ATR) 3259, 3071, 1649, 1640, 1596, 1536, 1476, 1341, 1340, 1320, 1076, 1041, 918, 847 cm^−1^; δ_H_ (300 MHz, DMSO-d_6_) 10.12 (1H, bs, CONHNH), 9.44 (1H, d, *J* 7, ArCONH), 9.15 (2H, d, *J* 2, Ar–H), 8.99 (1H, dd, *J* 2, Ar–H), 8.10 (1H, bs, CONHNH), 7.15 (1H, t, *J* 8, Ar–H), 6.78–6.62 (3H, m, Ar–H), 4.68 (1H, m, NHCH(CH_2_CH_2_SCH_3_)), 2.74–2.53 (2H, m, NHCH(CH_2_CH_2_SCH_3_)), 2.19–2.04 (5H, m, NHCH(CH_2_CH_2_SCH_3_); δ_C_ (75 MHz, DMSO-d_6_) 171.4 (CONHNH), 163.4 (ArCONH), 151.2 (*ipso*-Ar–C), 148.6 (*ipso*-Ar–C), 137.1 (*ipso*-Ar–C), 134.0 (*ipso*-Ar–C), 130.7 (Ar–C), 128.5 (Ar–C), 121.3 (Ar–C), 118.5 (Ar–C), 111.9 (Ar–C), 111.3 (Ar–C), 52.8 (NHCH(CH_2_CH_2_SCH_3_)), 31.2 (NHCH(CH_2_CH_2_SCH_3_)), 30.4 (NHCH(CH_2_CH_2_SCH_3_)), 15.1 (NHCH(CH_2_CH_2_SCH_3_)); *m*/*z* (ES^+^) 468 ([^35^Cl]MH^+^), 470 ([^37^Cl]MH^+^); HRMS (ES^+^) found [^35^Cl]MH^+^, 468.0743 (C_18_H_19_^35^ClN_5_O_6_S requires 468.0745).

(*S*)-6-chloro-2-ethyl-*N-*(1-oxo-1-(2-(4-(trifluoromethyl)phenyl)hydrazineyl)propan-2-yl)imidazo[1,2-a]pyridine-3-carboxamide, **34**, following the standard procedure B, *tert-*butyl (*S*)-(1-oxo-1-(2-(4-(trifluoromethyl) phenyl)hydrazineyl)propan-2-yl)carbamate (0.1 g, 0.35 mmol) and 6-chloro-2-ethylimidazo[1,2-*a*]pyridine-3-carboxylic acid were transformed to afford the title compound as a pale yellow solid (69 mg, 43%); m.p. 221–224 °C; *ν*_max._ (ATR) 3252, 2967, 2932, 1667, 1609, 1541, 1522, 1493, 1453, 1442, 1391, 1329, 1281, 1251, 1220, 1160, 1097, 1066, 1009, 995, 952, 906 cm^−1^; δ_H_ (400 MHz, DMSO-d_6_) 10.10 (1H, d, *J* 2, CONHNH), 9.01 (1H, d, *J* 2, Ar–H), 8.47 (1H, d, *J* 2, CONHNH), 8.33 (1H, d, *J* 7, NHCH(CH_3_)CO), 7.68 (1H, d, *J* 9, Ar–H), 7.51–7.45 (3H, m, Ar–H), 6.91 (2H, d, *J* 8, Ar–H), 4.60 (1H, q, *J* 7, NHCH(CH_3_)CO), 3.00 (2H, q, *J* 7, CH_2_CH_3_), 1.46 (3H, d, *J* 7, NHCH(CH_3_)CO), 1.26 (3H, t, *J* 7, CH_2_CH_3_); δ_C_ (100 MHz, DMSO-d_6_) 172.8 (CONHNH), 161.1 (CONH), 153.0 (*ipso*-Ar–C), 151.8 (*ipso*-Ar–C), 143.7 (*ipso*-Ar–C), 127.5 (*ipso*-Ar–C), 126.6 (Ar–C), 125.2 (Ar–C), 120.1 (*ipso*-Ar–C), 117.7 (Ar–C), 112.0 (Ar–C), 48.3 (NHCH(CH_3_)CO), 22.2 (CH_2_CH_3_), 18.2 (NHCH(CH_3_)CO), 13.5 (CH_2_CH_3_); *m*/*z* (ES^+^) 454 ([^35^Cl]MH^+^), 456 ([^37^Cl]MH^+^); HRMS (ES^+^) found [^35^Cl]MH^+^, 454.1256 (C_20_H_20_^35^ClF_3_N_5_O_2_ requires 454.1258).

(*S*)-6-chloro-2-ethyl-*N-*(4-(methylthio)-1-oxo-1-(2-(4-(trifluoromethyl)phenyl)hydrazineyl)butan-2-yl)imidazo[1,2-a]pyridine-3-carboxamide, **35**, following the standard procedure B, *tert-*Butyl *N-*[(1*S*)-3-(methylsulfanyl)-1-{*N′*-[4-(trifluoromethyl)phenyl]hydrazine carbonyl}propyl]carbamate (65 mg, 0.19 mmol) and 6-chloro-2-ethylimidazo[1,2-*a*]pyridine-3-carboxylic acid were transformed to afford the title compound as a white solid (53 mg, 55%); m.p. 227–230 °C; *ν*_max_ (ATR) 3248, 1664, 1612, 1526, 1487, 1417, 1394, 1374, 1328, 1291, 1189, 1158, 1108, 1066, 1013, 1000, 924, 897, 838 cm^−1^; δ_H_ (300 MHz, DMSO-d_6_) 10.16 (1H, d, *J* 2, CONHNH), 8.96 (1H, d, *J* 2, Ar–H), 8.48 (1H, d, *J* 2, CONHNH), 8.42 (1H, d, *J* 7, ArCONH), 7.67 (1H, d, *J* 9, Ar–H), 7.52–7.45 (3H, m, Ar–H), 6.92 (2H, d, *J* 8, Ar–H), 4.70 (1H, m, NHCH(CH_2_CH_2_SCH_3_)), 3.00 (2H, q, *J* 7, CH_2_CH_3_), 2.75–2.56 (2H, m, NHCH(CH_2_CH_2_SCH_3_)), 2.20–2.05 (5H, m, NHCH(CH_2_CH_2_SCH_3_), 1.28 (3H, t, *J* 7, CH_2_CH_3_); δ_C_ (75 MHz, DMSO-d_6_) 171.9 (CONHNH), 161.6 (CONH), 152.9 (*ipso*-Ar–C), 151.8 (*ipso*-Ar–C), 143.7 (*ipso*-Ar–C), 127.5 (*ipso*-Ar–C), 126.5 (Ar–C), 125.1 (Ar–C), 120.1 (*ipso*-Ar–C), 117.7 (Ar–C), 116.3 (*ipso*-Ar–C), 112.0 (Ar–C), 52.4 (NHCH(CH_2_CH_2_SCH_3_)), 31.2 (NHCH(CH_2_CH_2_SCH_3_)), 30.4 (NHCH(CH_2_CH_2_SCH_3_)), 22.2 (CH_2_CH_3_), 15.1 (NHCH(CH_2_CH_2_SCH_3_)), 13.4 (CH_2_CH_3_); δ_F_ (282 MHz, DMSO-d_6_) -59.32 (3F, s, CF_3_); *m*/*z* (ES^+^) 514 ([^35^Cl]MH^+^), 516 ([^37^Cl]MH^+^); HRMS (ES^+^) found [^35^Cl]MH^+^, 514.1290 (C_22_H_24_^35^ClF_3_N_5_O_2_S requires 514.1291).

(*S*)-6-chloro-*N-*(1-(2-(3-chlorophenyl)hydrazineyl)-1-oxopropan-2-yl)-2-ethylimidazo[1,2-a]pyridine-3-carboxamide, **36**, following the standard procedure B, *tert-*butyl (*S*)-(1-(2-(3-chlorophenyl)hydrazineyl)-1-oxopropan-2-yl)carbamate (0.1 g, 0.4 mmol) and 6-chloro-2-ethylimidazo[1,2-*a*]pyridine-3-carboxylic acid were transformed to afford the title compound as a white solid (28 mg, 17%); m.p. 213–215 °C; *ν*_max._ (ATR) 3258, 2978, 1666, 1608, 1605, 1538, 1492, 1455, 1417, 1394, 1370, 1317, 1269, 1248, 1218, 1159, 1139, 1110, 1075, 1044, 993, 950, 906, 850, 802 cm^−1^; δ_H_ (400 MHz, DMSO-d_6_) 10.00 (1H, d, *J* 2, CONHNH), 9.07 (1H, d, *J* 2, Ar–H), 8.31 (1H, d, *J* 7, NHCH(CH_3_)CO), 8.13 (1H, d, *J* 2, CONHNH), 7.68 (1H, d, *J* 9, Ar–H), 7.48 (1H, dd, *J* 9, 2, Ar–H), 7.15 (1H, t, *J* 6, Ar–H), 6.85 (1H, s, Ar–H), 6.74–6.70 (2H, m, Ar–H), 4.54 (1H, q, *J* 7, NHCH(CH_3_)CO), 3.00 (2H, q, *J* 7, CH_2_CH_3_), 1.46 (3H, d, *J* 7, NHCH(CH_3_)CO), 1.28 (3H, t, *J* 7, CH_2_CH_3_); δ_C_ (100 MHz, DMSO-d_6_) 172.8 (CONHNH), 161.1 (CONH), 151.9 (*ipso*-Ar–C), 151.3 (*ipso*-Ar–C), 143.7 (*ipso*-Ar–C), 134.1 (*ipso*-Ar–C), 130.7 (Ar–C), 127.6 (Ar–C), 125.3 (Ar–C), 120.1 (*ipso*-Ar–C), 118.3 (*ipso*-Ar–C), 117.7 (Ar–C), 116.3 (*ipso*-Ar–C), 112.0 (Ar–C), 111.4 (Ar–C), 48.3 (NHCH(CH_3_)CO), 22.2 (CH_2_CH_3_), 18.1 (NHCH(CH_3_)CO), 13.4 (CH_2_CH_3_); *m*/*z* (ES^+^) 420 ([^35,35^Cl]MH^+^), 422 ([^35,37^Cl]MH^+^), 424 ([^37,37^Cl]MH^+^); HRMS (ES^+^) found [^35,35^Cl]MH^+^, 420.0992 (C_19_H_20_^35,35^Cl_2_N_5_O_2_ requires 420.0994).

(*S*)-6-chloro-*N-*(1-(2-(3-chlorophenyl)hydrazineyl)-4-(methylthio)-1-oxobutan-2-yl)-2-ethylimidazo[1,2-a]pyridine-3-carboxamide, **37**, following the standard procedure B, *tert-*Butyl *N-*[(1*S*)-1-[*N′*-(3-chlorophenyl)hydrazinecarbonyl]-3-(methylsulfanyl)propyl] carbamate (70 mg, 0.23 mmol) and 6-chloro-2-ethylimidazo[1,2-*a*]pyridine-3-carboxylic acid were transformed to afford the title compound as a white solid (20 mg, 18%); m.p. 188–191 °C; *ν*_max_ (ATR) 3334, 2916, 1666, 1603, 1519, 1489, 1417, 1396, 1341, 1305, 1288, 1212, 1151, 1120, 1048, 998, 963, 872, 847, 814 cm^−1^; δ_H_ (400 MHz, DMSO-d_6_) 10.07 (1H, d, *J* 2, CONHNH), 9.04 (1H, d, *J* 2, Ar–H), 8.41 (1H, d, *J* 7, CONH), 8.15 (1H, d, *J* 2, CONHNH), 7.70 (1H, d, *J* 9, Ar–H), 7.48 (1H, dd, *J* 9, 2, Ar–H), 7.16 (1H, t, *J* 6, Ar–H), 6.86 (1H, s, Ar–H), 6.76–6.68 (2H, m, Ar–H), 4.59 (1H, q, *J* 7, NHCH(CH_2_CH_2_SCH_3_)), 3.00 (2H, q, *J* 7, CH_2_CH_3_), 2.68–2.56 (2H, m, NHCH(CH_2_CH_2_SCH_3_)), 2.12–2.06 (5H, m, NHCH(CH_2_CH_2_SCH_3_), 1.28 (3H, t, *J* 7, CH_2_CH_3_); δ_C_ (176 MHz, DMSO-d_6_) 172.0 (CONHNH), 161.6 (CONH), 152.0 (*ipso*-Ar–C), 151.3 (*ipso*-Ar–C), 143.8 (*ipso*-Ar–C), 134.1 (*ipso*-Ar–C), 130.7 (Ar–C), 127.6 (Ar–C), 125.3 (Ar–C), 120.1 (*ipso*-Ar–C), 118.3 (*ipso*-Ar–C), 117.7 (Ar–C), 116.4 (*ipso*-Ar–C), 112.0 (Ar–C), 111.4 (Ar–C), 52.0 (NHCH(CH_2_CH_2_SCH_3_)), 31.2 (NHCH(CH_2_CH_2_SCH_3_)), 31.1 (NHCH(CH_2_CH_2_SCH_3_)), 22.3 (CH_2_CH_3_), 15.1 (NHCH(CH_2_CH_2_SCH_3_)), 13.6 (CH_2_CH_3_); *m*/*z* (ES^+^) 480 ([^35,35^Cl]MH^+^), 482 ([^35,37^Cl]MH^+^), 484 ([^37,37^Cl]MH^+^); HRMS (ES^+^) found [^35,35^Cl]MH^+^, 480.1026 (C_21_H_24_^35,35^Cl_2_N_5_O_2_S requires 480.1028).

### 3.2. Biological Assessment

#### 3.2.1. Cell Lines

The bacterial species and cell lines used in this study can be found in Table 2.

The cell line utilized for cytotoxicity studies were the murine macrophage cell line RAW264.7 (ATCC TIB-71).

#### 3.2.2. Bacterial Growth Conditions

Mycobacterial species were cultured in either Middlebrook 7H9 broth or Middlebrook 7H10 agar media supplemented with albumin–dextrose–catalase (ADC) or oleic acid–albumin–dextrose–catalase (OADC) enrichments and 0.2% glycerol, 0.2% casamino acids, 24 μg/mL pantothenate, 1 μg/mL penicillin G, 10 μg/mL cyclohexamide, purchased from BD Biosciences. All reagents were purchased from Sigma-Aldrich, Gillingham, UK unless stated otherwise.

#### 3.2.3. Spontaneous Mutant Generation

*Mtb* mc^2^7000 mono-resistant mutants were generated by plating 1 × 10^8^ mid-log cells on solid media (7H11 agar supplemented with 10% OADC and 0.2% glycerol, 0.2% casamino acids, 24 μg/mL pantothenate, 1 μg/mL penicillin G, 10 μg/mL cyclohexamide) containing 0.1, 0.2, 0.4, 1.6 and 3.2 μg/mL of RIF or INH. Plates were incubated at 37 °C, 5% CO_2_ until the growth of colonies was visible. Spontaneous resistant mutants were confirmed by minimum inhibitory concentration (MIC) testing in liquid media as described below (REMA). The genomic DNA from validated mutants was extracted, and Next Generation Sequencing (NG*S*) analysis was performed to confirm mutation locations.

#### 3.2.4. Bacterial Growth Inhibition Assays

Bacterial minimum inhibition concentrations (MIC) were determined using the resazurin microtiter assay (REMA) method, according to Palomino et al. [35]. Stock solutions of the tested compounds were prepared in sterile dimethyl sulfoxide (DMSO), then diluted in Middlebrook 7H9 broth (Difco, Detroit, MI, USA) supplemented with oleic acid, albumin, dextrose and catalase (OADC enrichment) to obtain a final compound concentration range of 0.0625–64 μg/mL. A suspension of the test *Mycobacterium* was cultured in Middlebrook 7H9 broth supplemented with 10% OADC and 0.2% glycerol, 0.2% casamino acids, 24 μg/mL pantothenate, 1 μg/mL penicillin G, 10 μg/mL cyclohexamide and 0.05% Tween 80 for one week at 37 °C in an atmosphere of 5% CO_2_. The concentration was adjusted at McFarland 0.5 and diluted to 1 × 10^6^ CFU/mL and diluted in growth media 1:25. A 100 μL of the inoculum was added to each well of a 96-well microplate together with 100 μL of the compounds. The plate was incubated at 37 °C in an atmosphere of 5% CO_2_. After 5 days, 10 μL 0.2% (*w*/*v*) resazurin (solubilized in sterile water) was added. MIC_99_ was defined as the lowest concentration resulting in 99% inhibition of growth of *Mycobacterium* resulting in no colour change. Samples were set up in quadruplet and tested in two independent assays.

#### 3.2.5. Determination of Cytotoxicity of Compounds against Mammalian Macrophages

RAW 264.7 cell lines were purchased from American Type Culture Collection, Middlesex, UK (ATCC1 TIB-71™). Cells were cultured in DMEM high glucose medium, supplemented with 10% FBS, 2 mM L-Glutamine and 1% penicillin/streptomycin, at 37 °C in a humidified incubator containing 5% CO_2_. Cells were passaged after reaching 90% confluence.

Cells, in logarithmic growth, were plated in 96-well plates at a density of 1 × 10^4^ cells/well in 200 µL of media and incubated for 24 h. The media on the cells was removed and replaced with 180 µL of fresh media. To each respective well, 20 µL of test compound at a concentration of 1 mM was added, resulting in a final compound concentration of 100 µM. To control wells, DMSO drug solvent was added. In all wells, the final concentration of DMSO did not exceed 0.1%. Following incubation for a further 96 h at 37 °C in a humidified incubator containing 5% CO_2_, all media was removed and replaced with 100 µL of fresh media. To each well, 20 µL of 3-(4,5-dimethylthiazol-2-yl)-5-(3-carboxymethoxyphenyl)-2-(4-sulfophenyl)-2H-tetrazolium, inner salt (MT*S*) assay solution (Promega, Madison, WI, USA) was added, and the plate was incubated in the dark at 37 °C for 4 h. Finally, the absorbance of each well was recorded at 490 nm using a microplate reader. The percentage of cell viability was calculated relevant to the compound solvent (DMSO) control.

## 4. Conclusions

With the end TB strategy faltering and the pipeline for new TB drugs being minimal, the requirement for novel architectures to combat the rise in drug-resistant TB infections is of paramount importance. The existing benzoxa-[2,1,3]-diazole amino acid hydrazide scaffolds were modified in order to demonstrate that the removal of the benzoxa-[2,1,3]-diazole moiety and replacement, utilising a scaffold hopping approach with relevant pharmacophores from existing anti-tubercular drugs, was a feasible tactic. The use of known chemical moieties present in molecules with anti-TB activity, such as quinoline or imidazo [1,2-a]pyridine-3-carboxy substituents, have provided a variety of antibacterial activities similar or greater to that with the benzoxa-[2,1,3]-diazole moiety and molecules with defined selectivity against drug-resistant strains of *Mtb* (Table 1 and [16]). In addition, the greater antibacterial activity observed between 3-chlorophenyl-substituted hydrazides compared to the 4-trifluorophenyl-substituted hydrazides remains to be elucidated; however, this may indicate a preferential binding pocket in the target site, favouring meta-substituted aromatic rings.

Overall, *N-*substituted amino acid hydrazides have provided a versatile scaffold for elaboration with known pharmacophores from existing drugs to provide active and clinically translatable anti-tubercular drugs. Whilst the exact mechanism of action is not understood, phenotypic observations have shown that these compounds favour isoniazid-resistant *Mtb* strains and so may target a pathway upstream of the site of INH activity or one involving perturbation of the ETC within mycobacteria. This hypothesis is yet to be tested and is currently under investigation.

## Figures and Tables

**Table 1:** Results of synthesis of substituted amino acid hydrazides and the antibacterial activity and cytotoxicity.

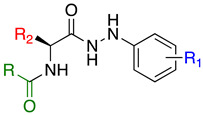									
				MIC (μM)					
				*Mtb*			*Mbv* BCG		Mammalian Cell Toxicity
R	R_1_	R_2_	Compound	WT	Rif ^R^	INH ^R^	+pan	-pan	
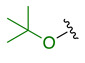	4-CF_3_	CH_3_	**6**	46	92	92	-	-	13%
		(CH_2_)_2_SCH_3_	**7**	79	79	78	157	157	>80%
	3-Cl	CH_3_	**8**	51	13	3	204	204	<10%
		(CH_2_)_2_SCH_3_	**9**	22	21	3	171	171	<5%
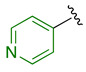	4-CF_3_	CH_3_	**10**	182	182	182	-	-	<5%
		(CH_2_)_2_SCH_3_	**11**	155	155	155	155	155	66%
	3-Cl	CH_3_	**12**	201	201	25	201	201	<5%
		(CH_2_)_2_SCH_3_	**13**	84	84	21	169	169	15%
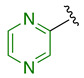	4-CF_3_	CH_3_	**14**	-	-	-	-	-	0%
		(CH_2_)_2_SCH_3_	**15**	155	155	155	-	-	55%
	3-Cl	CH_3_	**16**	50	50	6	50	50	25%
		(CH_2_)_2_SCH_3_	**17**	84	42	11	84	168	60%
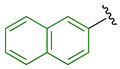	4-CF_3_	CH_3_	**18**	80	80	40	80	80	59%
		(CH_2_)_2_SCH_3_	**19**	35	35	35	69	69	>90%
	3-Cl	CH_3_	**20**	87	87	22	44	44	33%
		(CH_2_)_2_SCH_3_	**21**	-	-	19	-	-	60%
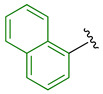	4-CF_3_	CH_3_	**22**	80	80	80	159	80	15%
		(CH_2_)_2_SCH_3_	**23**	69	69	69	69	69	40%
	3-Cl	CH_3_	**24**	-	-	44	-	-	<5%
		(CH_2_)_2_SCH_3_	**25**	75	37	19	-	-	43%
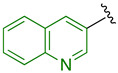	4-CF_3_	CH_3_	**26**	80	80	80	-	159	30%
		(CH_2_)_2_SCH_3_	**27**	138	35	69	138	138	>90%
	3-Cl	CH_3_	**28**	-	-	87	-	-	70%
		(CH_2_)_2_SCH_3_	**29**	37	37	9	75	75	60%
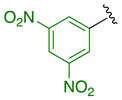	4-CF_3_	CH_3_	**30**	36	36	36	18	18	>90%
		(CH_2_)_2_SCH_3_	**31**	8	4	8	16	16	>90%
	3-Cl	CH_3_	**32**	78	78	20	39	39	75%
		(CH_2_)_2_SCH_3_	**33**	34	9	17	34	34	>90%
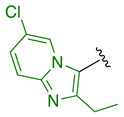	4-CF_3_	CH_3_	**34**	35	35	35	35	35	67%
		(CH_2_)_2_SCH_3_	**35**	-	-	62	-	-	>90%
	3-Cl	CH_3_	**36**	76	76	9	76	76	78%
		(CH_2_)_2_SCH_3_	**37**	33	33	8	33	33	>90%
			**Isoniazid**	0.2	0.07	-	0.4	0.4	
			**Rifampicin**	0.02	-	0.02	0.02	0.02	
			**Ethambutol**	4	4	4	20	20	
			**Ethionamide**	6	6	6	12	12	
			**Erythromycin**	0.2	0.17	0.17	0.09	0.09	
			**Kanamycin**	-	-	-	-	-	

WT = Wild type *Mtb* strain; Rif ^R^ = Rifampicin resistant *Mtb* strain; INH ^R^ = Isoniazid resistant *Mtb* strain; +pan = Pantothenate present in growth media; -pan = Pantothenate not present in growth media; - = Not active at the maximum assay concentration (64 μg/mL).

**Table 2:** Results of synthesis of the substituted amino acid hydrazides and the antibacterial activity and cytotoxicity.

Strain	Genotype	Comments	Reference
*Mtb* mc^2^7000	ΔRD1(esx1):GFP Δ*panCD*	Deletion of esx1 or RD1 responsible for virulence factor secretion, ESAT-6 and CFP-10 replaced with GFP. Pantothenate auxotroph.	W. Jacobs, Albert Einstein College of Medicine [32]
*Mbv* BCG	ΔRD1-14, 17-20	Bacillus Calmette–Guérin (BCG) Pasteur; Vaccine strain.	[33]
*Mtb* mc^2^7000 pMV261	ΔRD1(esx1):GFP Δ*panCD;* Kan ^R^	As *Mtb* mc^2^7000, contains pMV261 empty vector.	This study [34]
*Mbv* BCG pMV261	ΔRD1-14, 17-20*;* Kan ^R^	As *Mbv* BCG, contains pMV261 empty vector.	This study [34]
*Mtb* AKB7002	ΔRD1(esx1):GFP Δ*panCD;* Kan ^R^ *rpoB* (H445Y)	As *Mtb* mc^2^7000 pMV261, generated spontaneous mutant, rifampicin-resistant, *rpoB* His445→Lys.	This study
*Mtb* AKB7021	ΔRD1(esx1):GFP Δ*panCD;* Kan ^R^ *katG* (S315N)	As *Mtb* mc^2^7000 pMV261, generated spontaneous mutant, isoniazid-resistant, *katG* Ser315→Asp.	This study

Kan ^R^ = Kanamycin resistant strain.

## Supplementary Materials

The following are available online at https://www.mdpi.com/1420-3049/25/10/2387/s1, Figure S1: Representative comparison data between our current compounds (this study) and our lead benzoxa-[2,1,3]-diazoles compounds from our previous study, Figure S2: ^1^H and ^13^C NMR for all new compounds produced in this study.
