# Supplementary material for: Identification of Substituted Amino Acid Hydrazides as Novel Anti-Tubercular Agents, Using a Scaffold Hopping Approach
Source: Molecules. 2020 May 21;25(10):2387. doi: 10.3390/molecules25102387 (PMC7287914; doi:10.3390/molecules25102387)

Supplementary Materials

Identification of Substituted Amino Acid Hydrazides as Novel Anti-Tubercular Agents, Using a Scaffold Hopping Approach

Alistair K. Brown [1], Ahmed K.B. Aljohani [1], Fatimah M.A. Alsalem [1], Joseph L. Broadhead [2], Jason H. Gill [3,4], Yucheng Lu [1] and Jonathan D. Sellars [1,4,*]

1 Biosciences Institute, Faculty of Medical Sciences, Newcastle University, Newcastle upon Tyne NE2 4HH, UK; alistair.brown2@newcastle.ac.uk (A.K.B.); a.aljohani2@newcastle.ac.uk (A.K.B.A.); f.m.a.alsalem2@newcastle.ac.uk (F.M.A.A.); y.lu48@newcastle.ac.uk (Y.L.)

2 Arcinova, Taylor Drive, Alnwick NE66 2DH, UK; joseph190597@googlemail.com

3 Translational and Clinical Research Institute, Faculty of Medical Sciences, Newcastle University, Newcastle upon Tyne NE2 4HH, UK; jason.gill@newcastle.ac.uk

4 School of Pharmacy, Faculty of Medical Sciences, King George VI Building, Newcastle upon Tyne NE1 7RU, UK

* Correspondence: jon.sellars@newcastle.ac.uk; Tel.: +44-191-2082357

Figure S1: Representative comparison data between our current compounds (this study) and our lead benzoxa-[2,1,3]-diazoles compounds from our previous study.

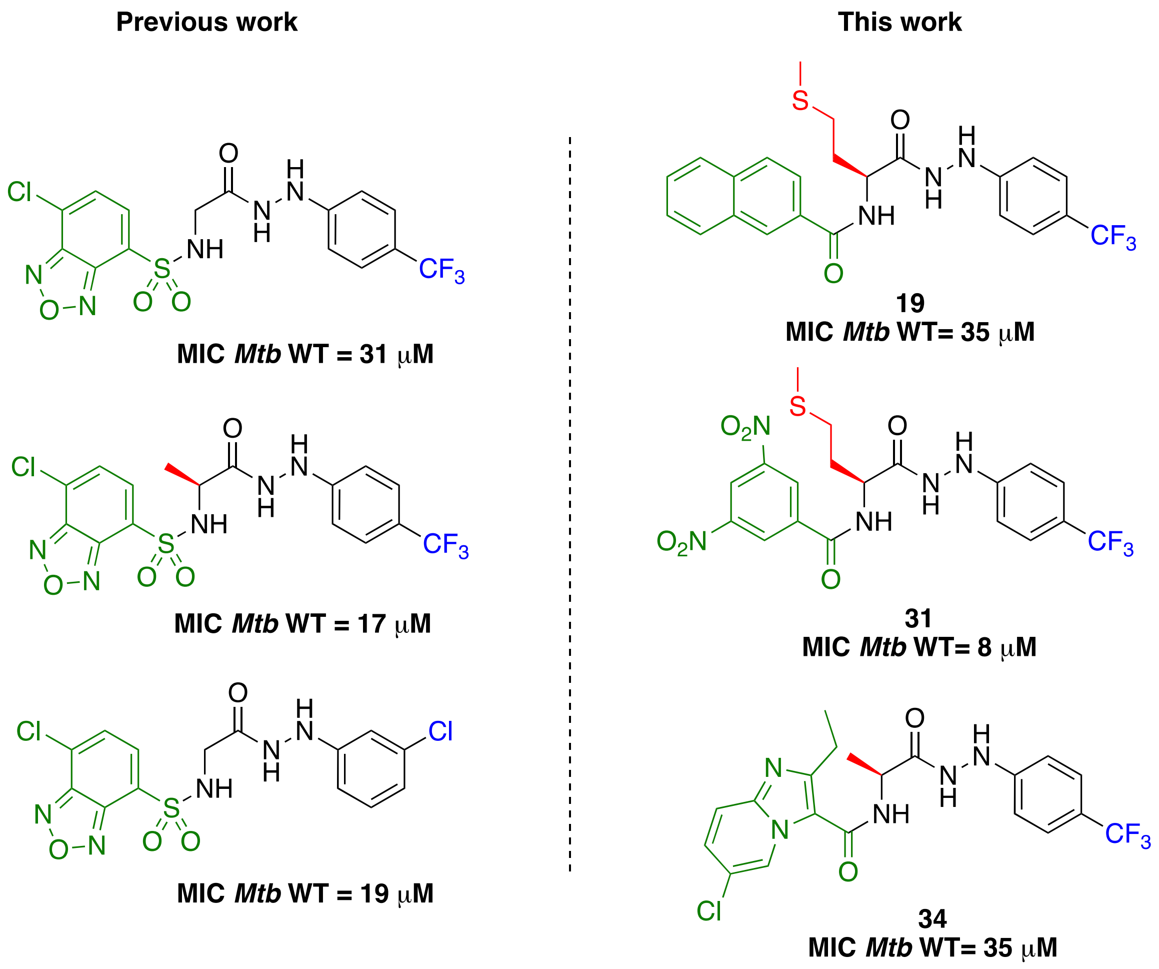

Figure S2: ^{1}H and ^{13}C NMR for all new compounds produced in this study.
Compound 7

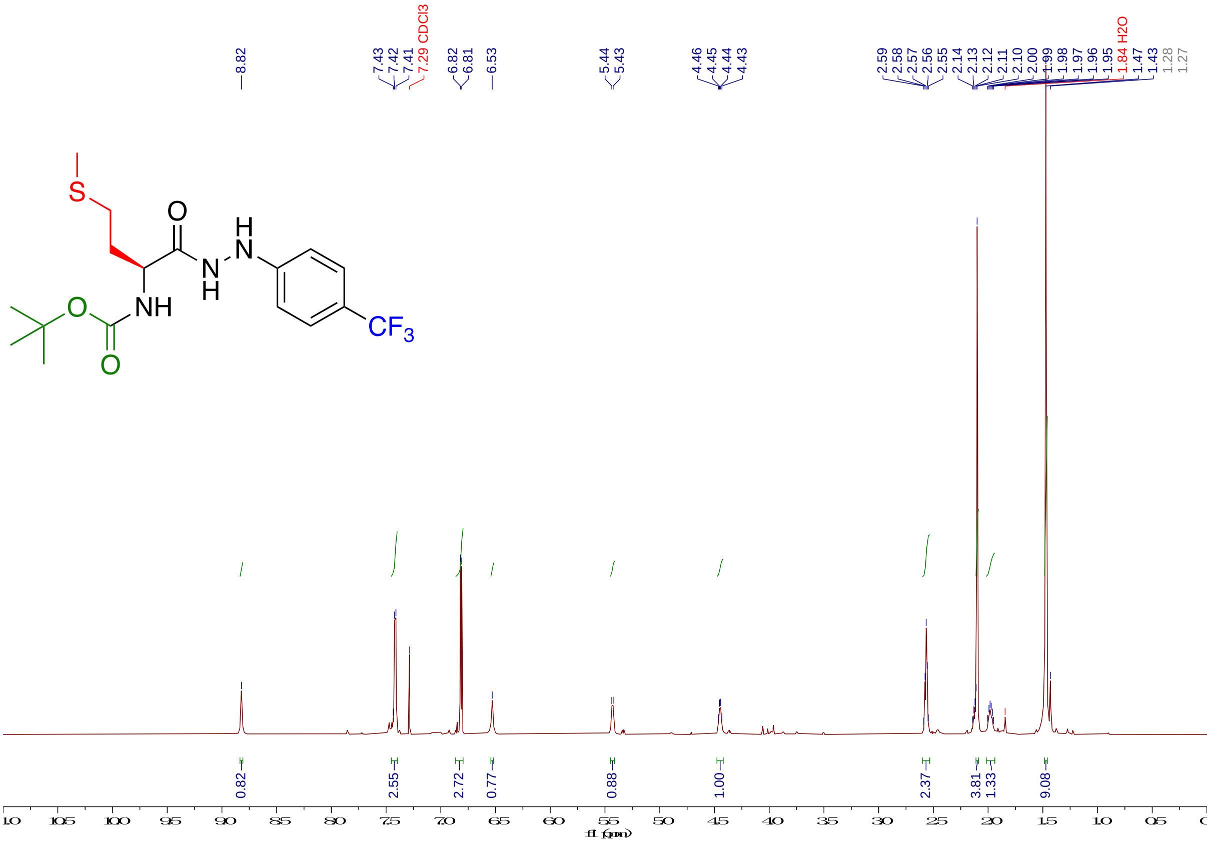

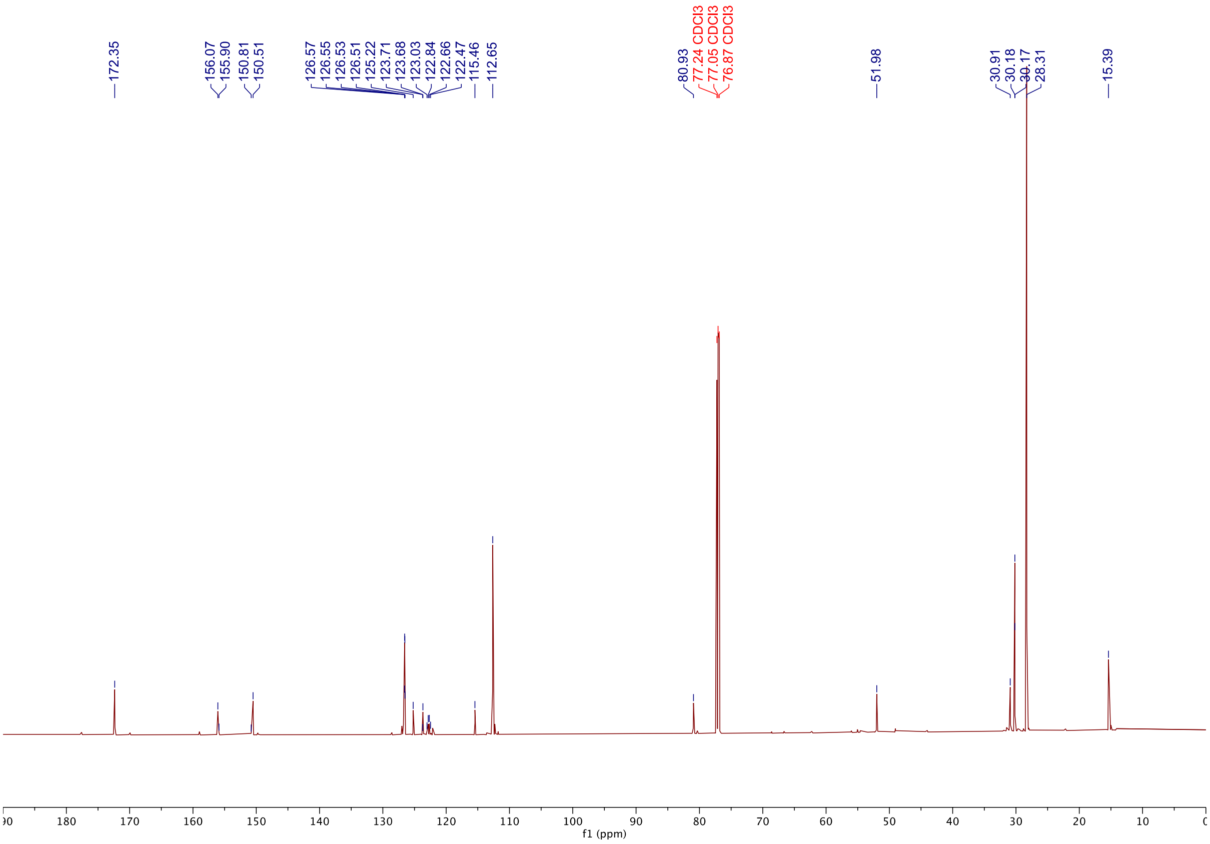

Compound 8

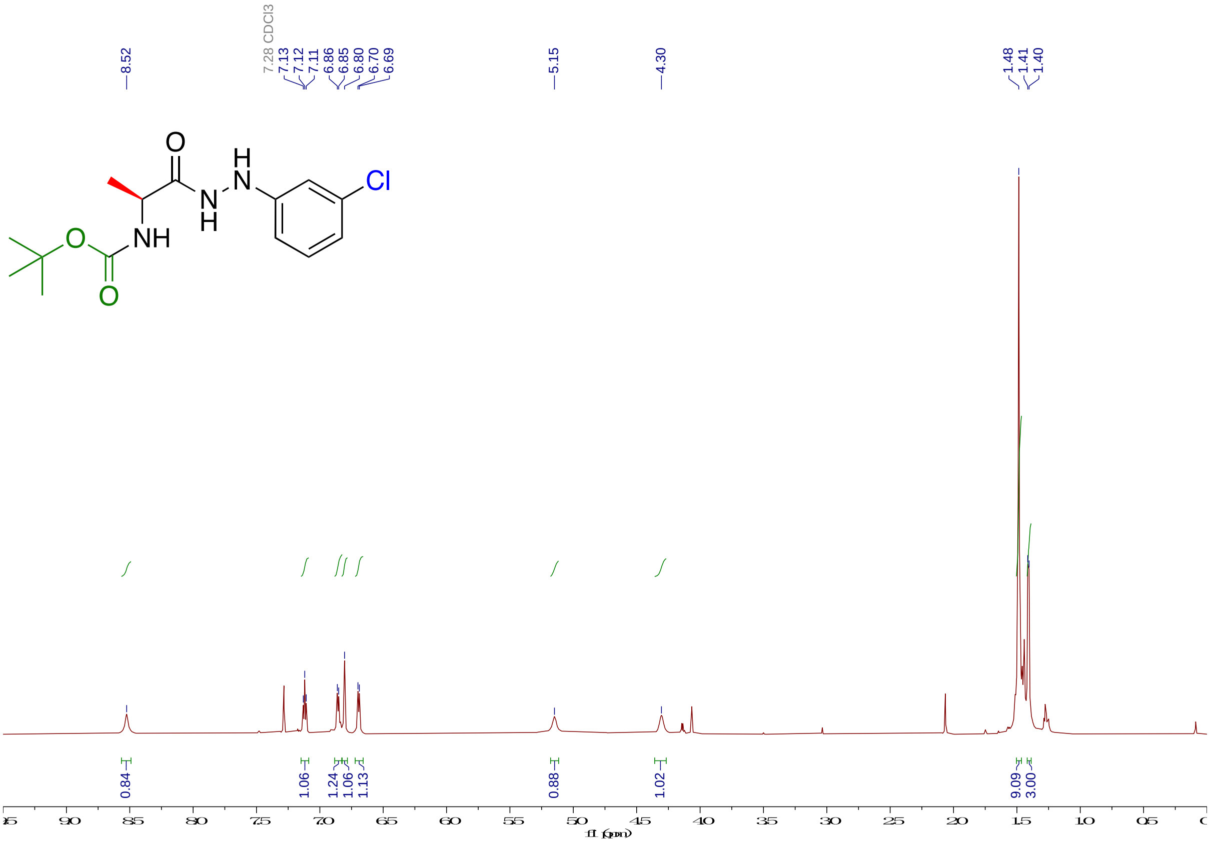
8.52
7.28 CDCl3
7.13
7.12
7.11
6.86
6.85
6.80
6.70
6.69
5.15
4.30
1.48
1.41
1.40
0.84
1.06
1.24
1.06
1.13
0.88
1.02
9.09
3.00
Cl
f1 (ppm)

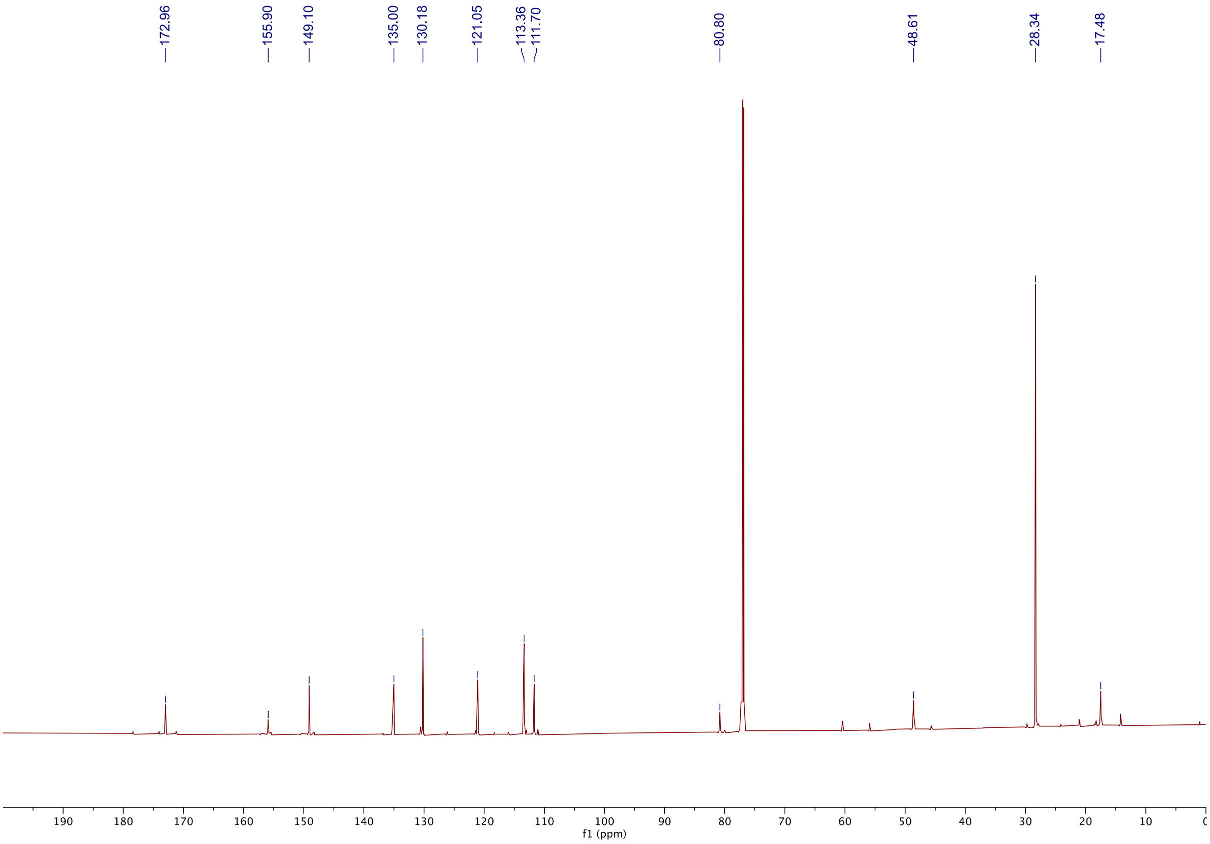
172.96
155.90
149.10
135.00
130.18
121.05
113.36
111.70
80.80
48.61
28.34
17.48
f1 (ppm)

Compound 9

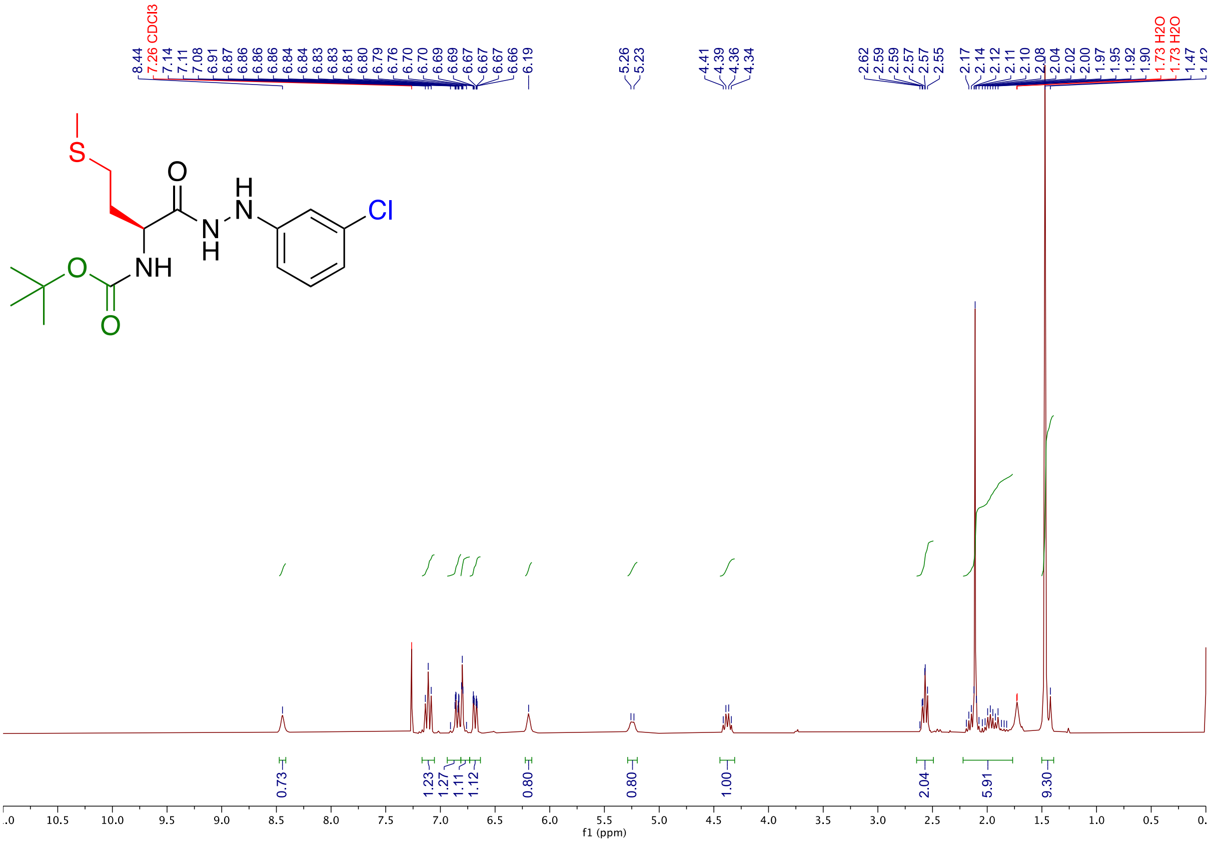

S
O
H
N
N
H
Cl
NH
O
O
8.44
7.26 CDCl3
7.14
7.11
7.08
6.91
6.87
6.86
6.86
6.84
6.84
6.83
6.81
6.80
6.79
6.76
6.70
6.69
6.69
6.67
6.67
6.67
6.66
6.19
5.26
5.23
4.41
4.39
4.36
4.34
2.62
2.59
2.59
2.57
2.57
2.55
2.17
2.14
2.12
2.11
2.10
2.08
2.04
2.02
2.00
1.97
1.95
1.92
1.90
1.73 H2O
1.73 H2O
1.47
0.73
1.23
1.27
1.11
1.12
0.80
0.80
1.00
2.04
5.91
9.30
f1 (ppm)

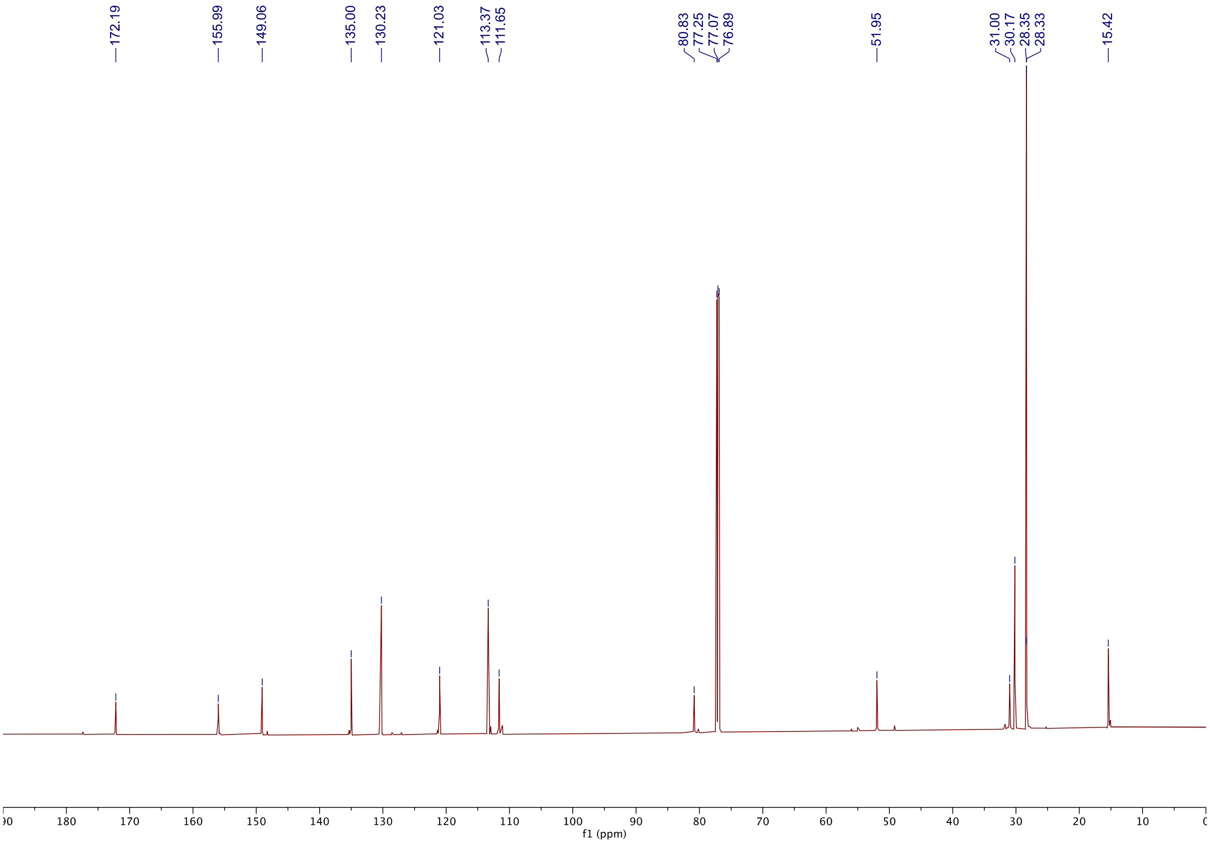

172.19
155.99
149.06
135.00
130.23
121.03
113.37
111.65
80.83
77.25
77.07
76.89
51.95
31.00
30.17
28.35
28.33
15.42
f1 (ppm)

Compound 10

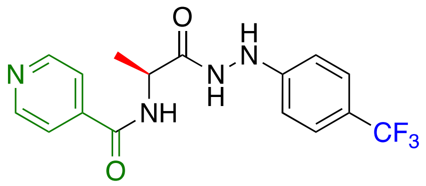

N
O
NH
O
N
H
H
N
CF3

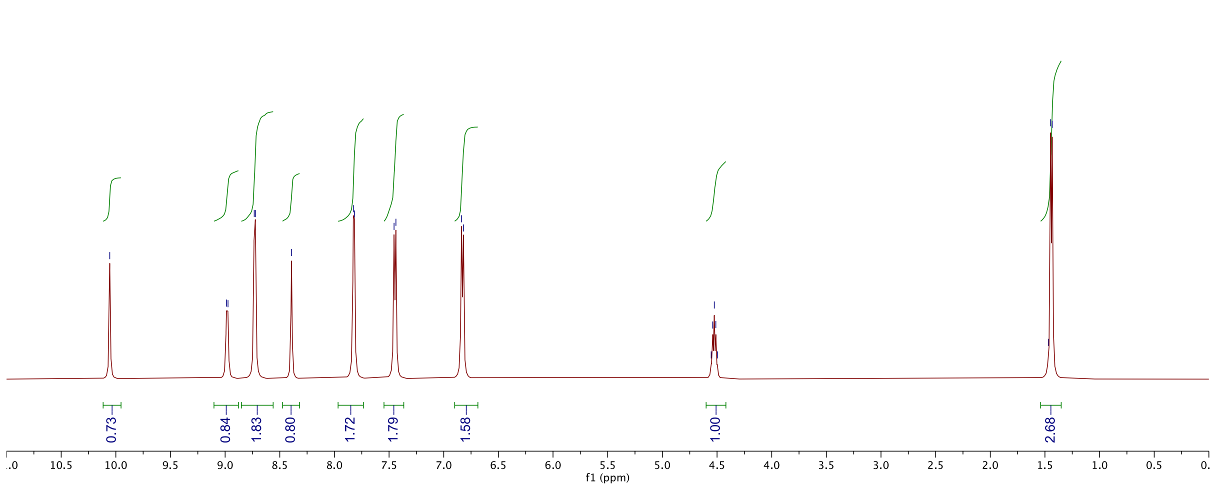

10.06
8.99
8.97
8.73
8.72
8.39
7.83
7.82
7.45
7.44
6.84
6.82
4.55
4.54
4.52
4.51
4.50
1.47
1.45
1.43
0.73
0.84
1.83
0.80
1.72
1.79
1.58
1.00
2.68
f1 (ppm)

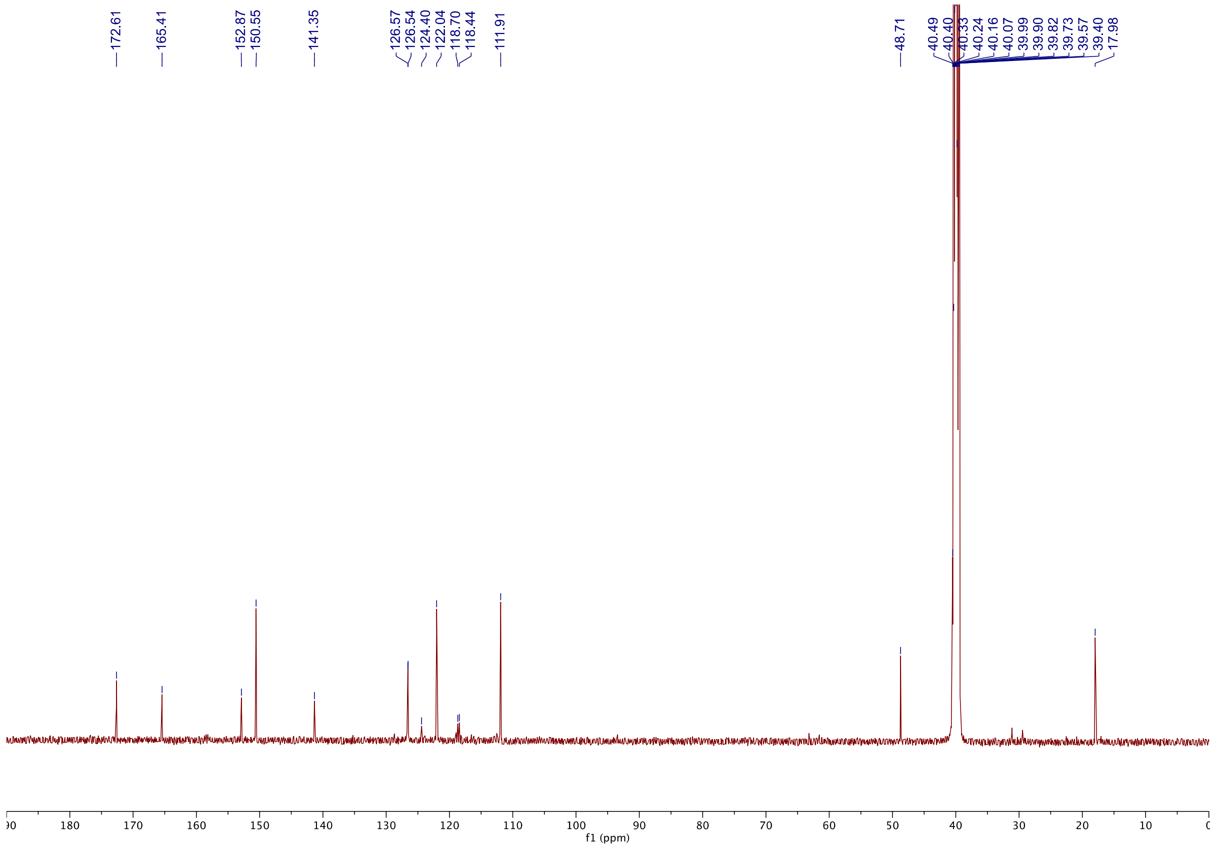

172.61
165.41
152.87
150.55
141.35
126.57
126.54
124.40
122.04
118.70
118.44
111.91
48.71
40.49
40.40
40.33
40.24
40.16
40.07
39.99
39.90
39.82
39.73
39.57
39.40
17.98
f1 (ppm)

Compound 11

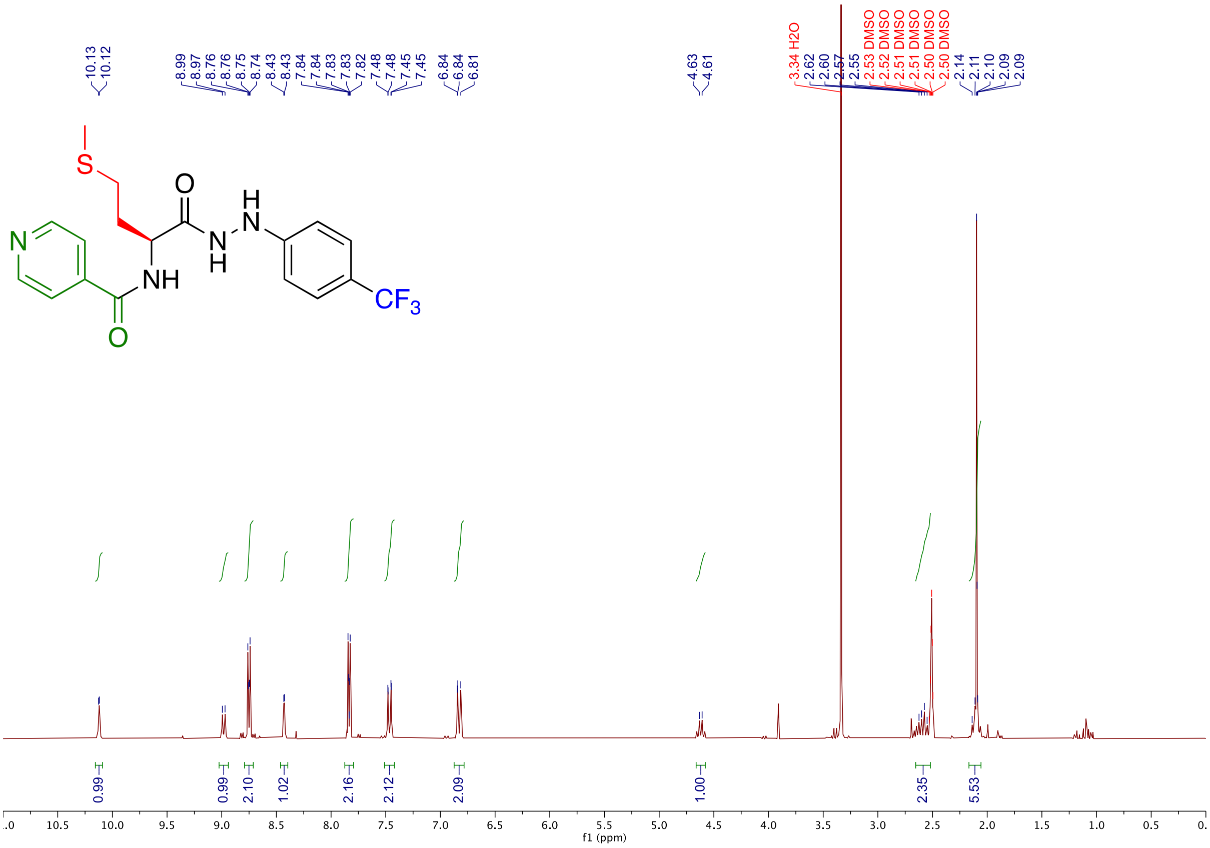

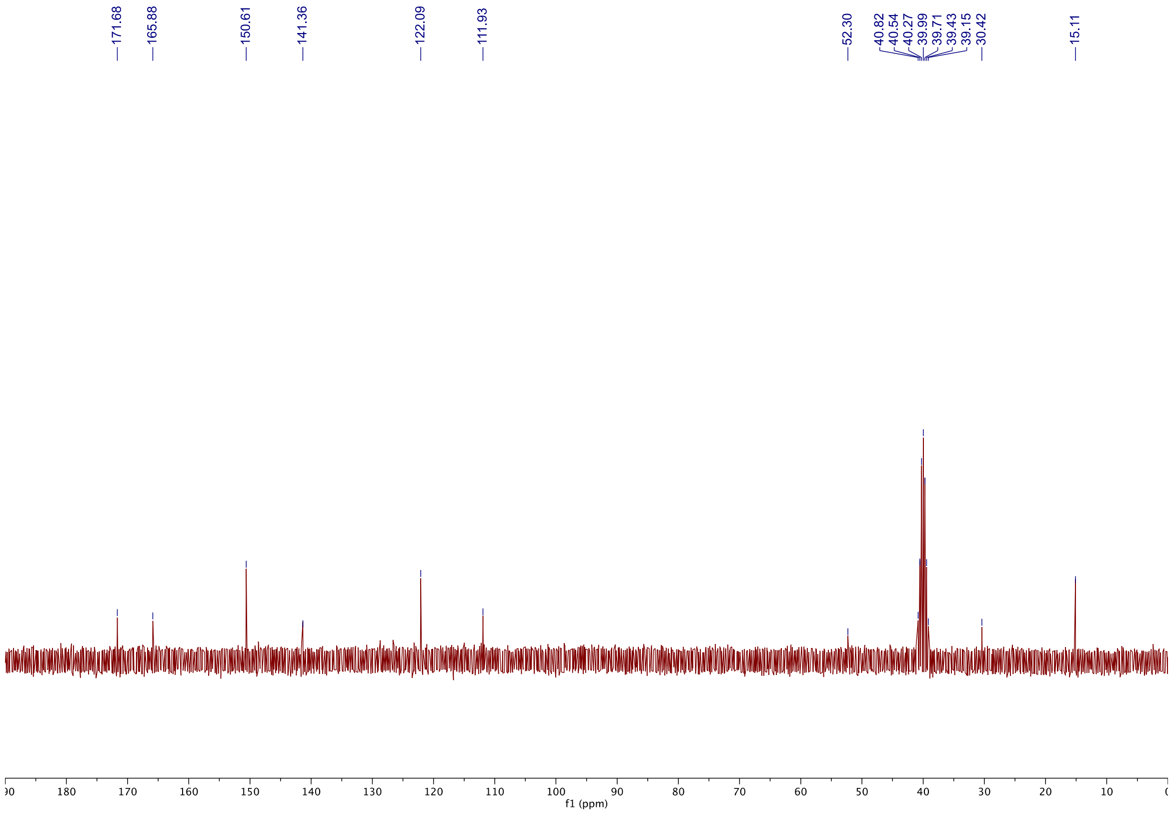

Compound 12

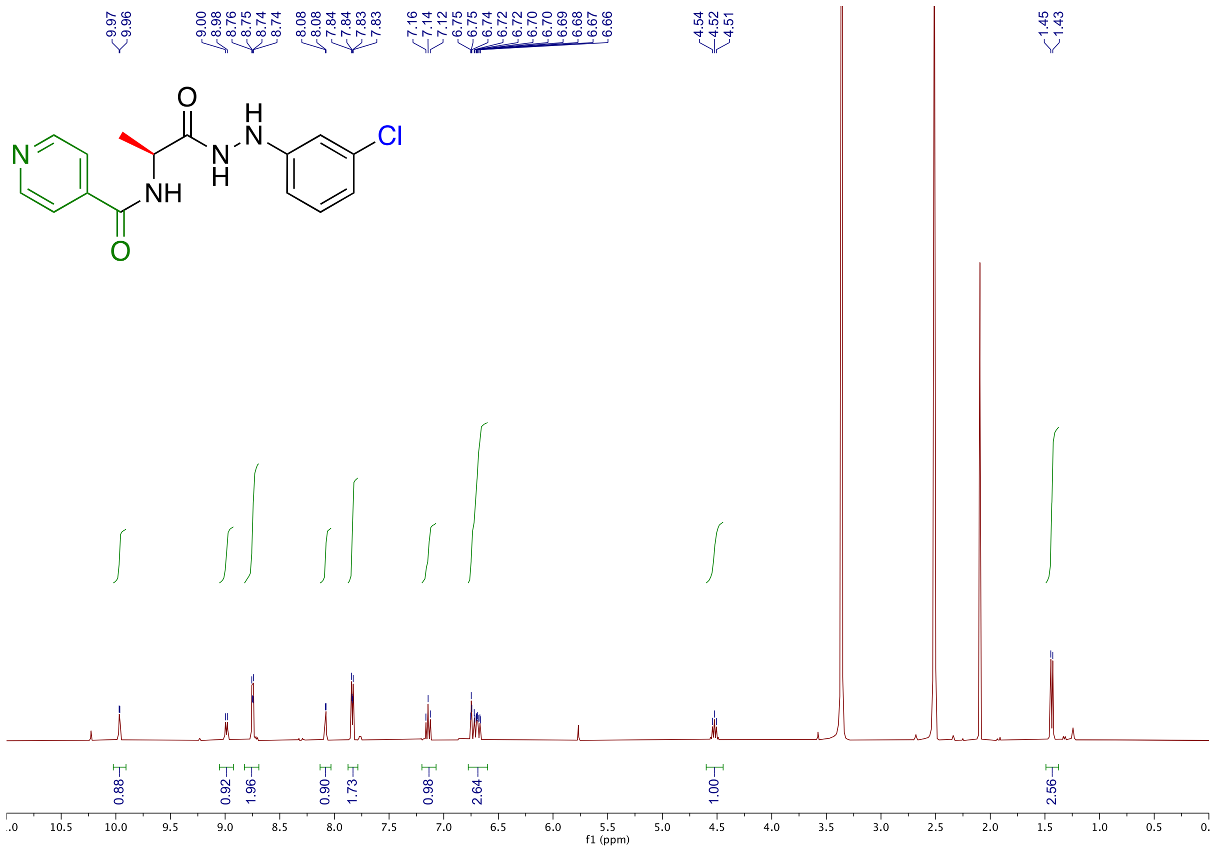
9.97
9.96
9.00
8.98
8.76
8.75
8.74
8.74
8.08
8.08
7.84
7.84
7.83
7.83
7.16
7.14
7.12
6.75
6.75
6.74
6.74
6.72
6.72
6.70
6.70
6.69
6.68
6.67
6.66
4.54
4.52
4.51
1.45
1.43
O
H
N
N
H
Cl
N
NH
O
0.88
0.92
1.96
0.90
1.73
0.98
2.64
1.00
2.56
f1 (ppm)

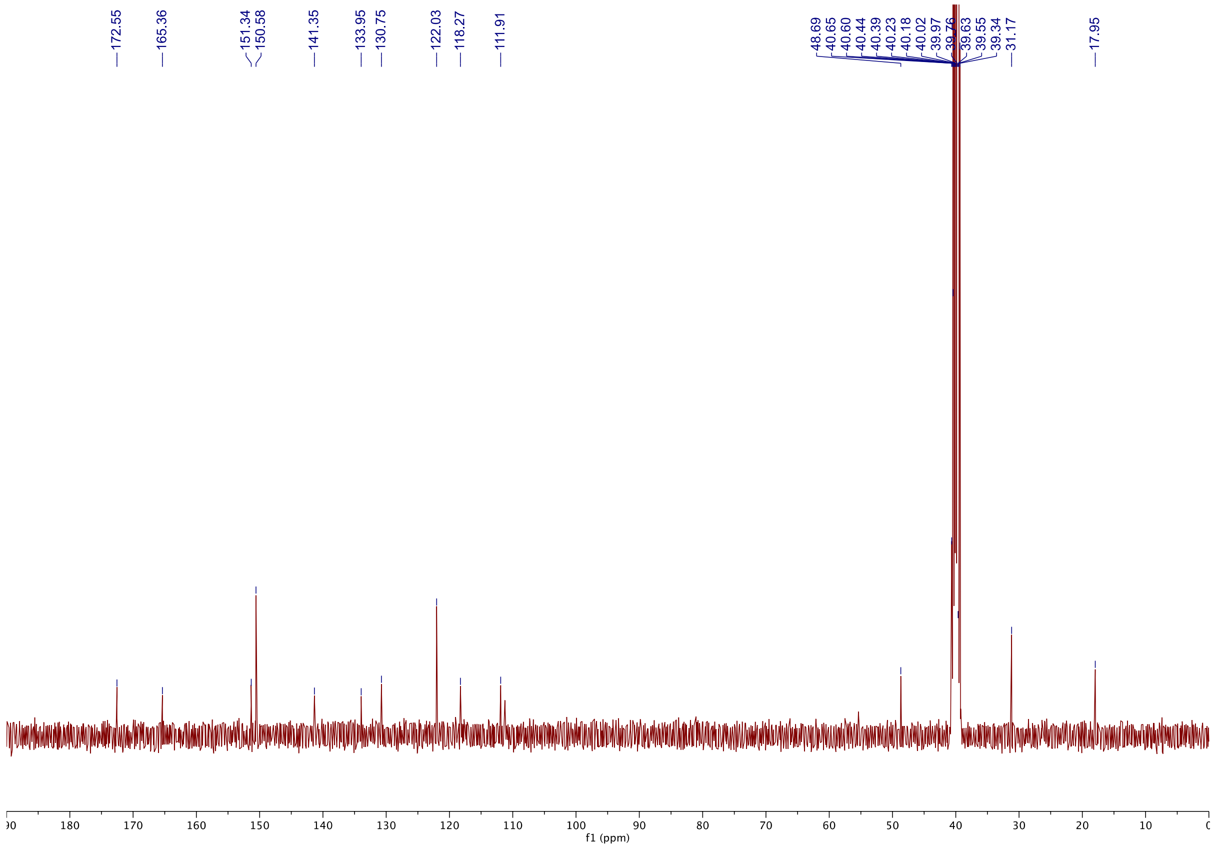
172.55
165.36
151.34
150.58
141.35
133.95
130.75
122.03
118.27
111.91
48.69
40.65
40.60
40.44
40.39
40.23
40.18
40.02
39.97
39.76
39.63
39.55
39.34
31.17
17.95
f1 (ppm)

Compound 13

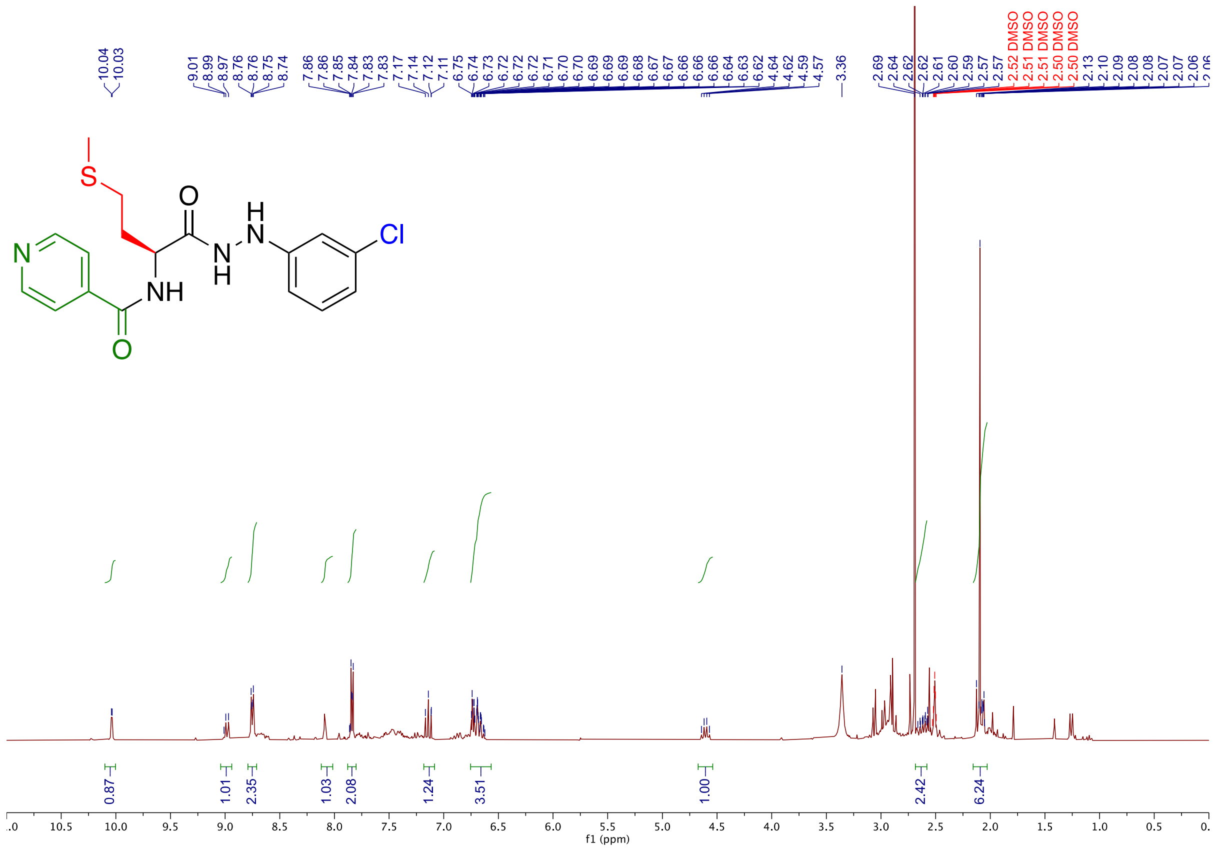

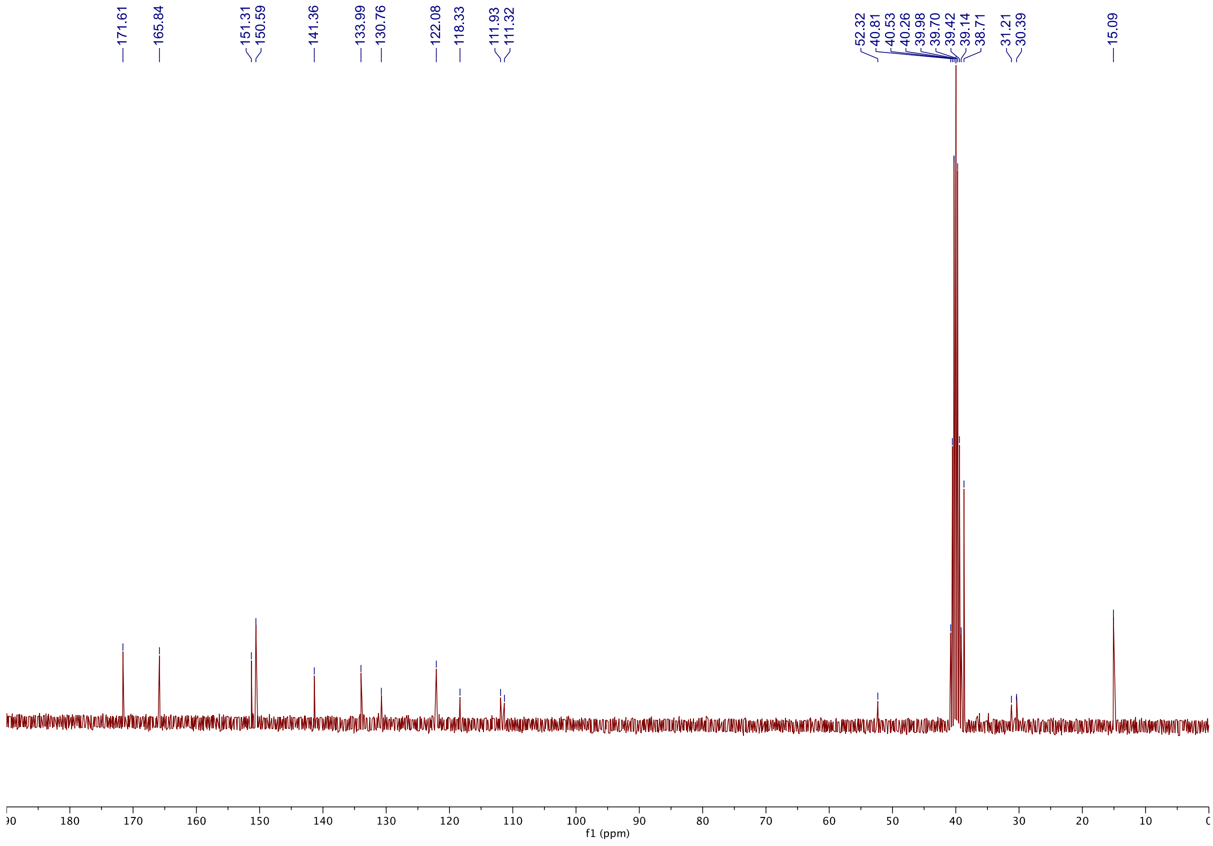

Compound 14

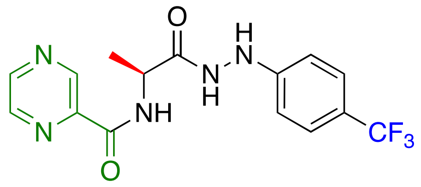

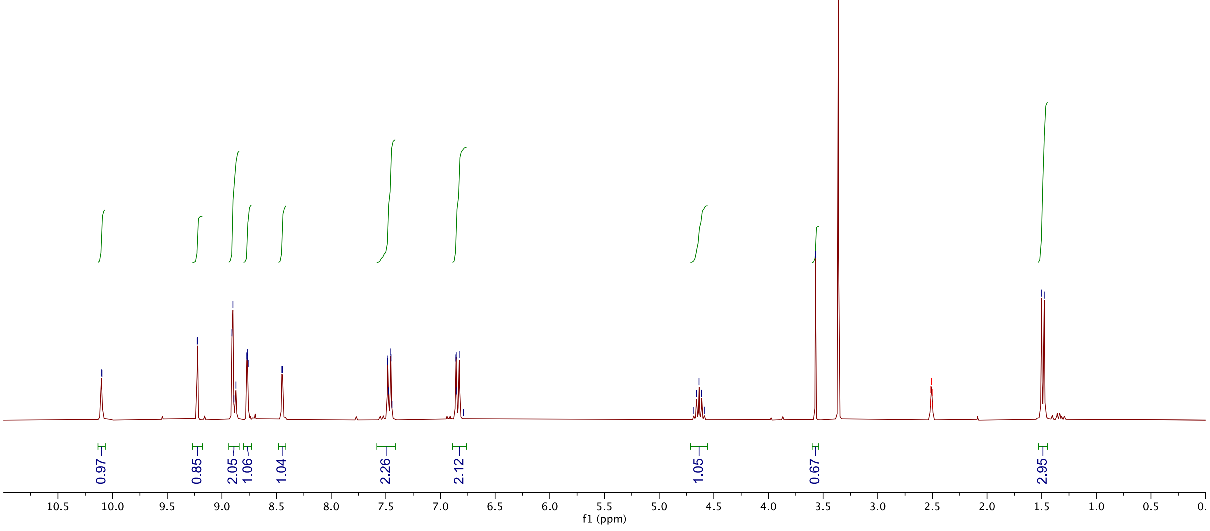

10.10
10.10
9.23
9.22
8.91
8.90
8.89
8.87
8.77
8.76
8.76
8.45
8.44
7.48
7.48
7.48
7.45
7.45
7.44
6.86
6.86
6.85
6.83
6.79
4.68
4.66
4.63
4.61
4.59
3.57
3.36 H2O
2.52 DMSO
2.51 DMSO
2.51 DMSO
2.50 DMSO
2.50 DMSO
1.50
1.48
0.97
0.85
2.05
1.06
1.04
2.26
2.12
1.05
0.67
2.95
f1 (ppm)

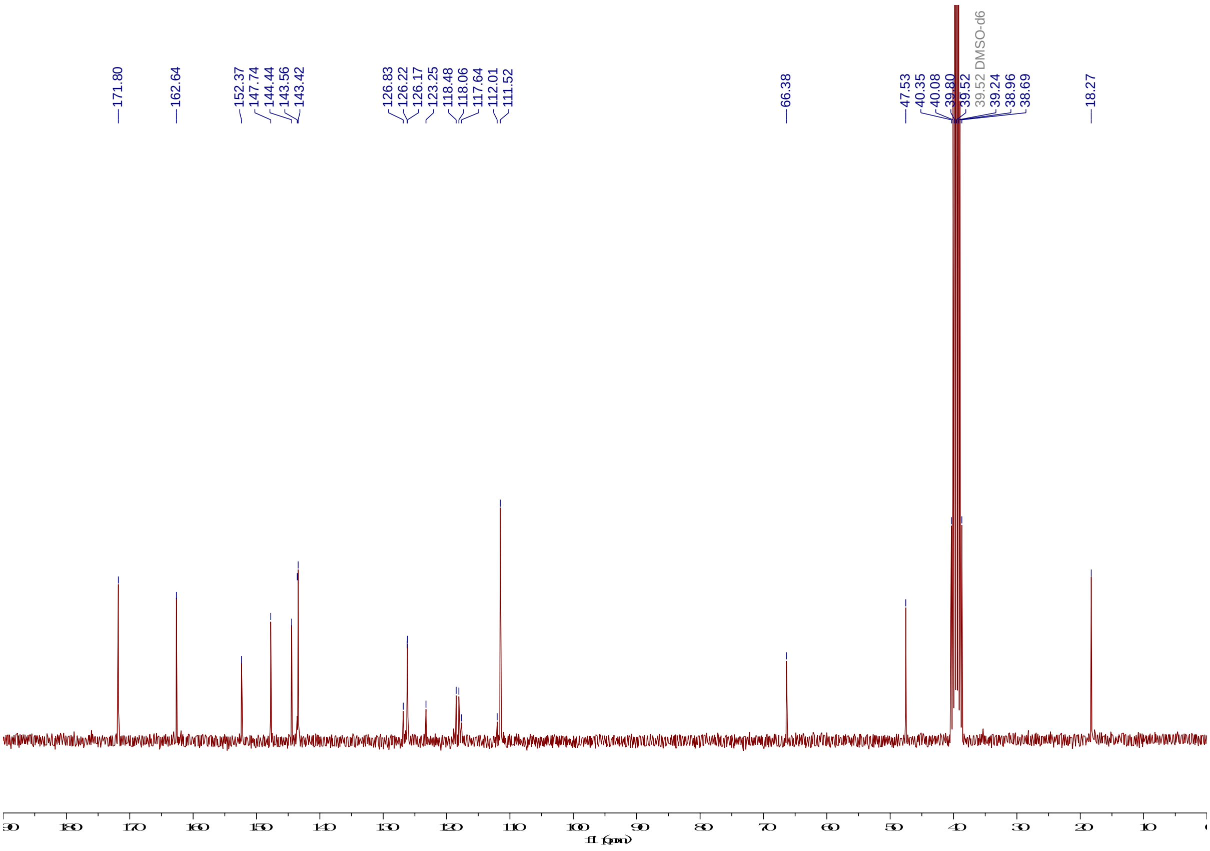

171.80
162.64
152.37
147.74
144.44
143.56
143.42
126.83
126.22
126.17
123.25
118.48
118.06
117.64
112.01
111.52
66.38
47.53
40.35
40.08
39.80
39.52
39.52 DMSO-d6
39.24
38.96
38.69
18.27
f1 (ppm)

Compound 15

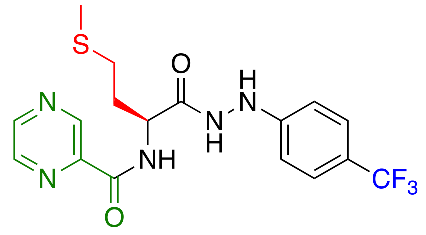

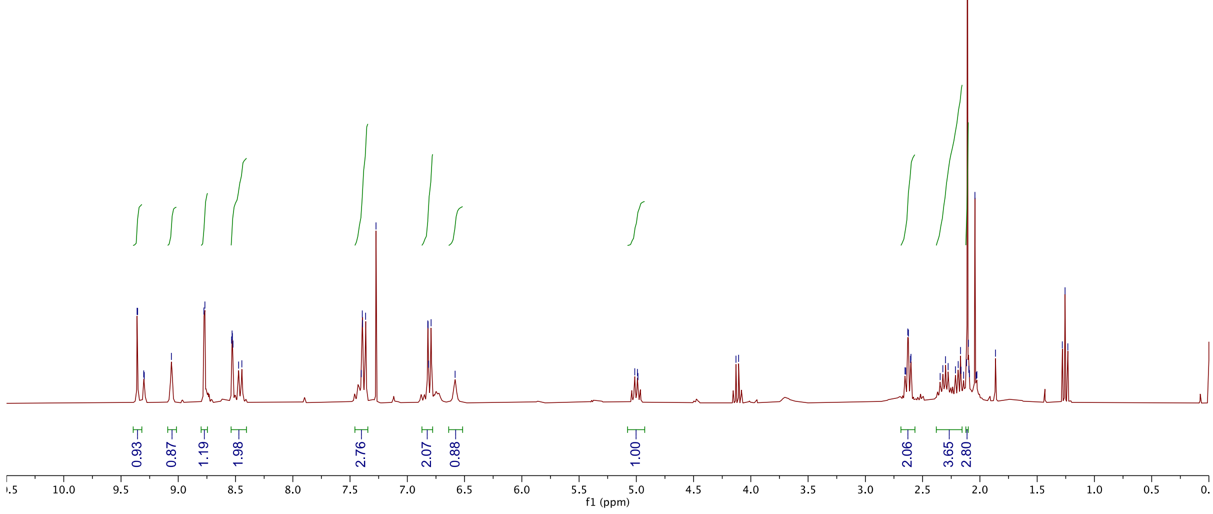
9.36
9.35
9.30
9.30
9.06
8.78
8.77
8.53
8.53
8.53
8.52
8.47
8.45
7.40
7.39
7.39
7.36
7.27
6.82
6.82
6.81
6.79
6.58
5.01
4.99
4.99
4.13
4.11
2.65
2.65
2.63
2.62
2.61
2.60
2.35
2.32
2.30
2.28
2.21
2.19
2.17
2.16
2.14
2.12
2.11
2.10
2.10
2.09
2.04
2.03
2.03
1.86
1.28
1.26
1.23
0.93
0.87
1.19
1.98
2.76
2.07
0.88
1.00
2.06
3.65
2.80
f1 (ppm)

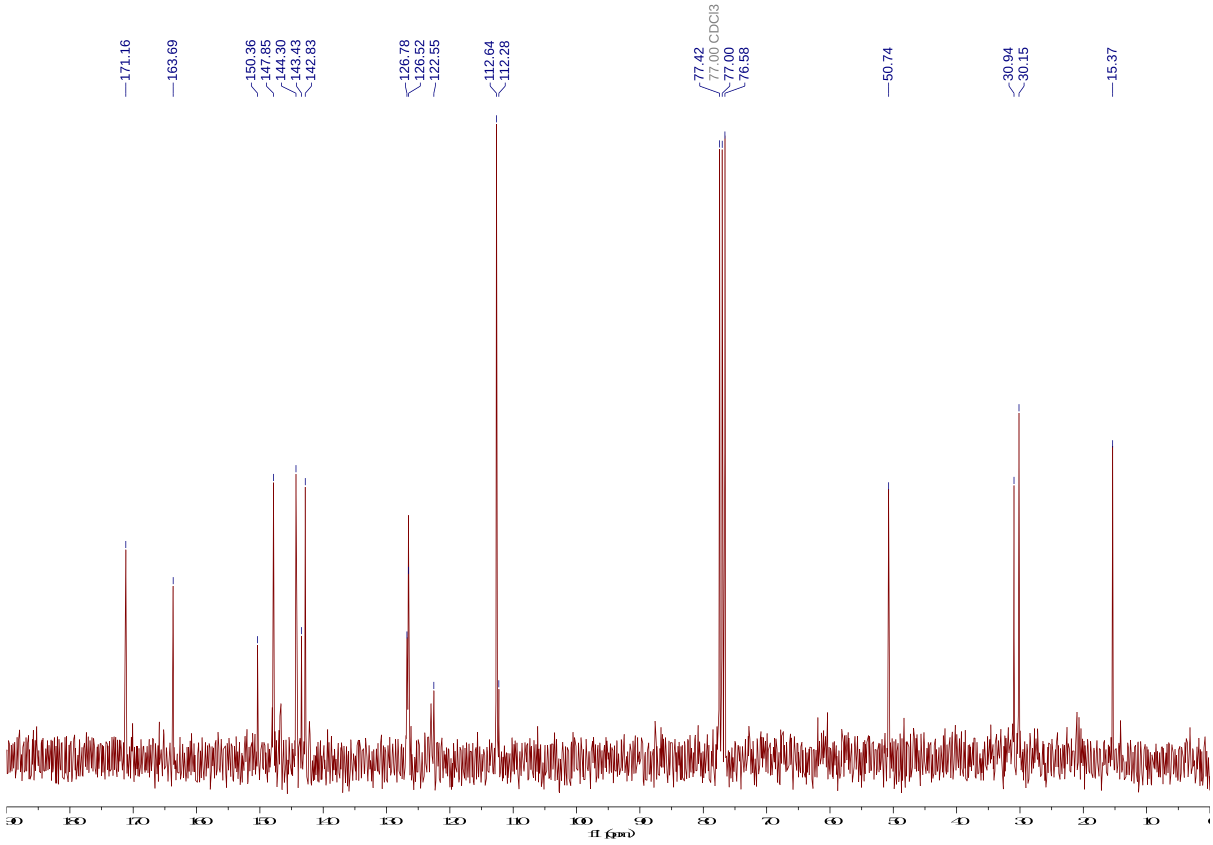
171.16
163.69
150.36
147.85
144.30
143.43
142.83
126.78
126.52
122.55
112.64
112.28
77.42
77.00 CDCl3
77.00
76.58
50.74
30.94
30.15
15.37

Compound 16

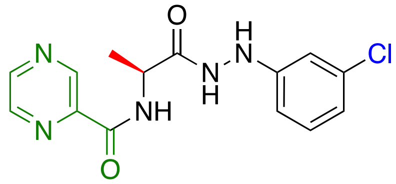

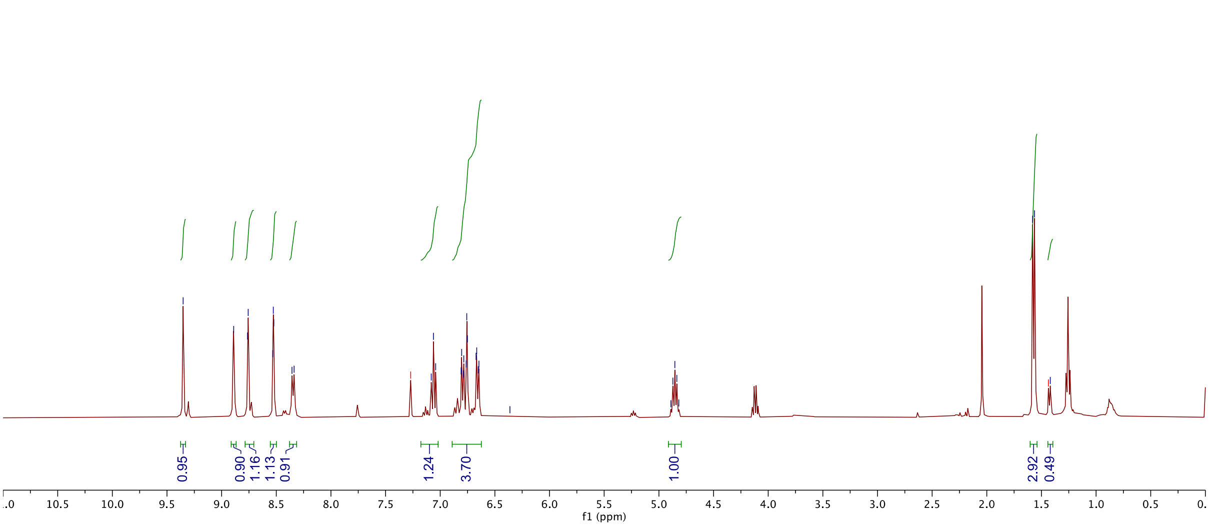
9.35
8.89
8.76
8.76
8.53
8.53
8.52
8.36
8.34
7.27 CDCl3
7.08
7.06
7.04
6.81
6.80
6.79
6.78
6.76
6.76
6.75
6.67
6.67
6.65
6.65
6.36
4.89
4.87
4.85
4.84
4.82
1.58
1.56
1.44 H2O
1.42
0.95
0.90
1.16
1.13
0.91
1.24
3.70
1.00
2.92
0.49
f1 (ppm)

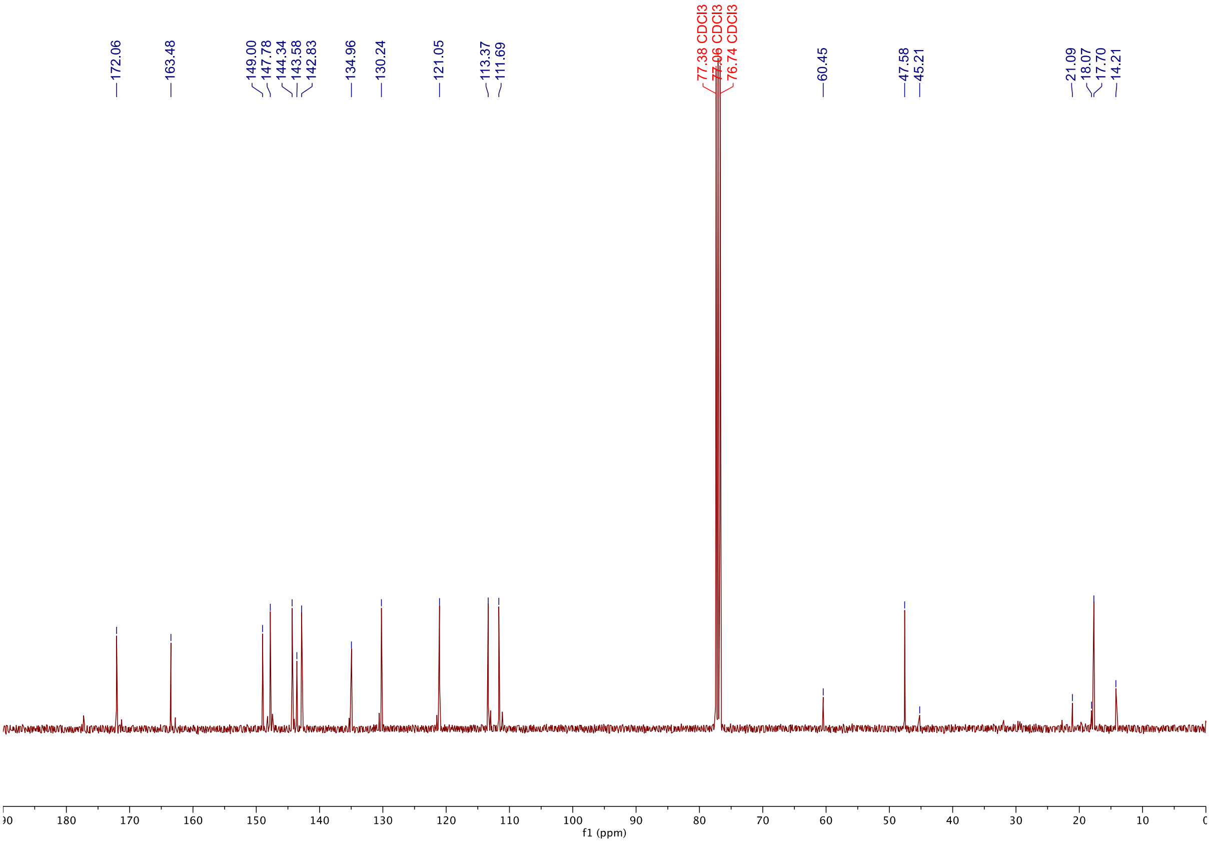
172.06
163.48
149.00
147.78
144.34
143.58
142.83
134.96
130.24
121.05
113.37
111.69
77.38 CDCl3
77.06 CDCl3
76.74 CDCl3
60.45
47.58
45.21
21.09
18.07
17.70
14.21
f1 (ppm)

Compound 17

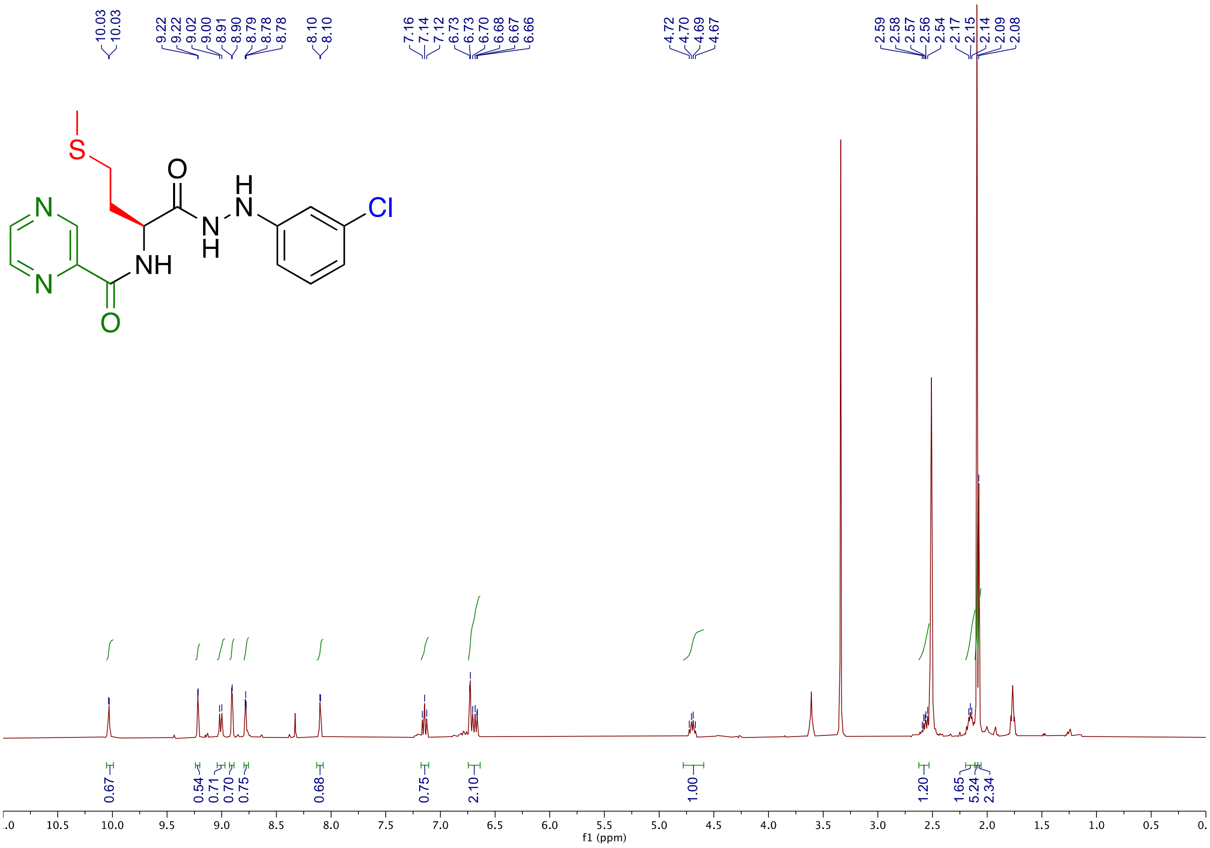

S
O
H
N
N
H
Cl
N
N
NH
O
10.03 10.03
9.22 9.22 9.02 9.00 8.91 8.90 8.79 8.78 8.78
8.10 8.10
7.16 7.14 7.12 6.73 6.73 6.70 6.68 6.67 6.66
4.72 4.70 4.69 4.67
2.59 2.58 2.57 2.56 2.54 2.17 2.15 2.14 2.09 2.08
0.67 0.54 0.71 0.70 0.75 0.68 0.75 2.10 1.00 1.20 1.65 5.24 2.34
f1 (ppm)

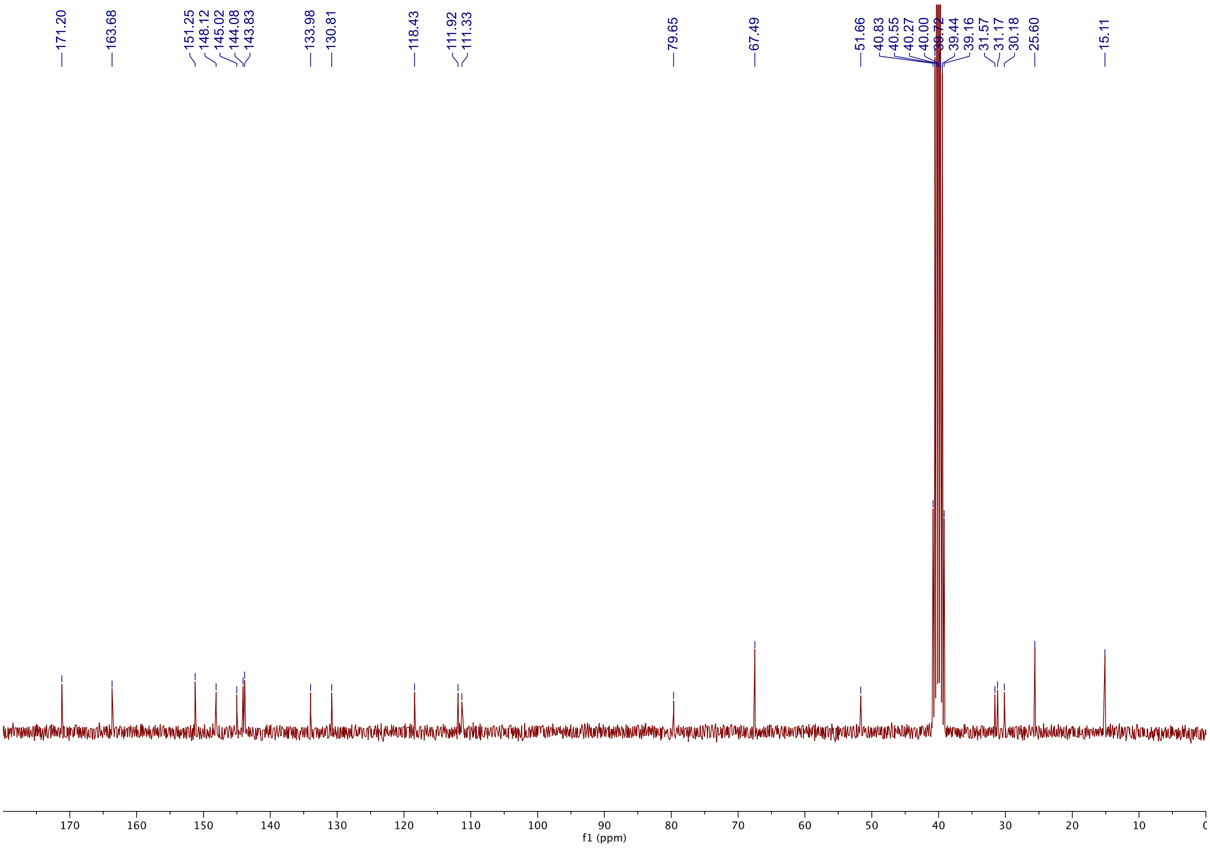

171.20
163.68
151.25 148.12 145.02 144.08 143.83
133.98 130.81
118.43
111.92 111.33
79.65
67.49
51.66 40.83 40.55 40.27 40.00 39.72 39.44 39.16 31.57 31.17 30.18 25.60
15.11
f1 (ppm)

Compound 18

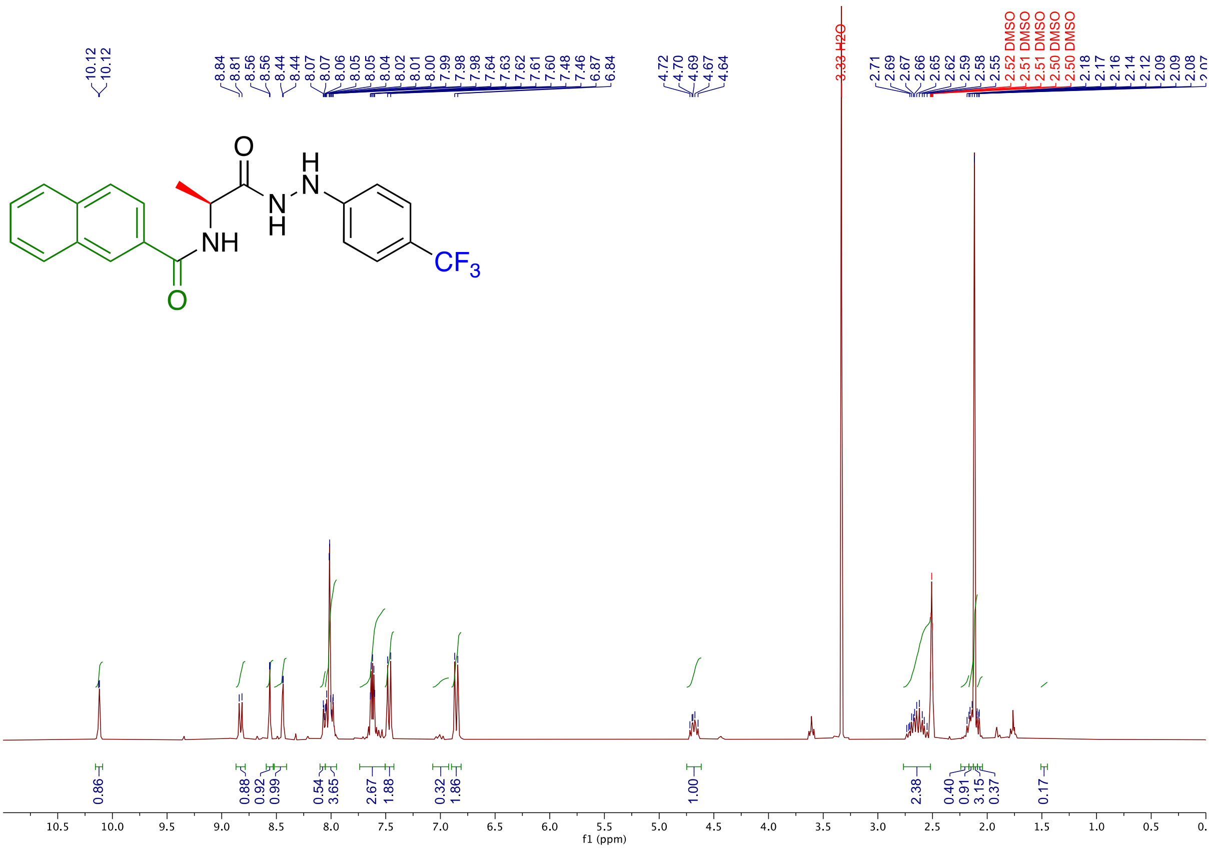

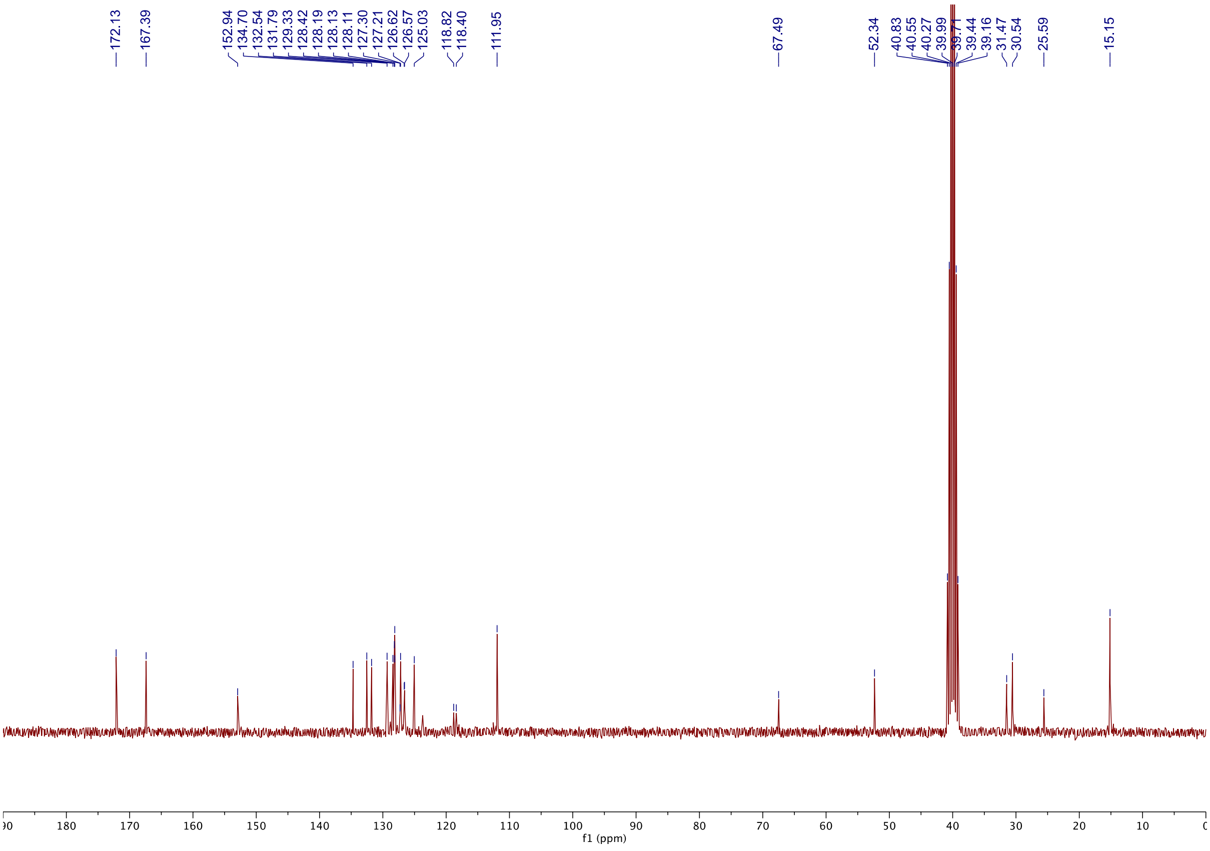

Compound 19

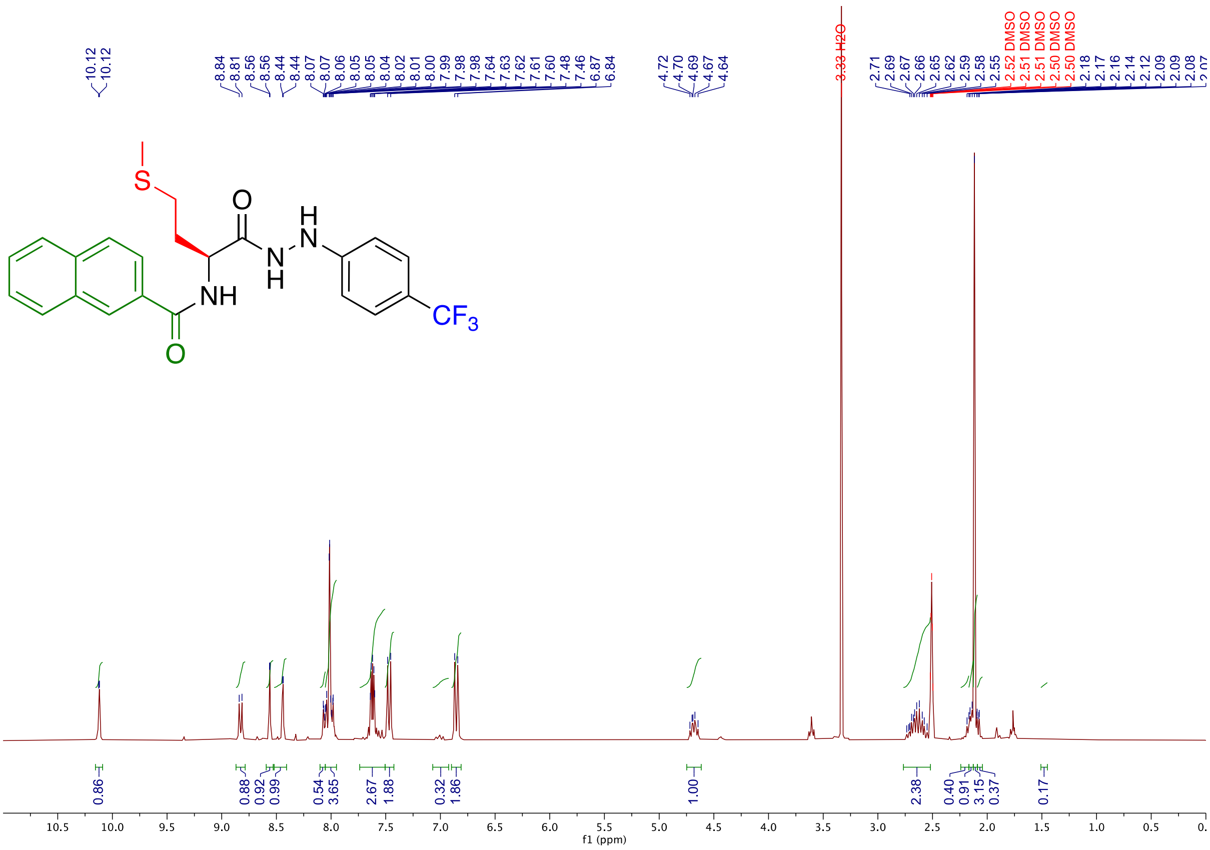
10.12
10.12
8.84
8.81
8.56
8.56
8.44
8.44
8.07
8.06
8.05
8.05
8.04
8.02
8.01
8.00
7.99
7.98
7.98
7.64
7.63
7.62
7.61
7.60
7.48
7.46
6.87
6.84
4.72
4.70
4.69
4.67
4.64
3.33 H2O
2.71
2.69
2.67
2.66
2.65
2.62
2.59
2.58
2.55
2.52 DMSO
2.51 DMSO
2.51 DMSO
2.50 DMSO
2.50 DMSO
2.18
2.17
2.16
2.14
2.12
2.09
2.08
S
O
H
N
N
H
NH
CF3
O
0.86
0.88
0.92
0.99
0.54
3.65
2.67
1.88
0.32
1.86
1.00
2.38
0.40
0.91
3.15
0.37
0.17
f1 (ppm)

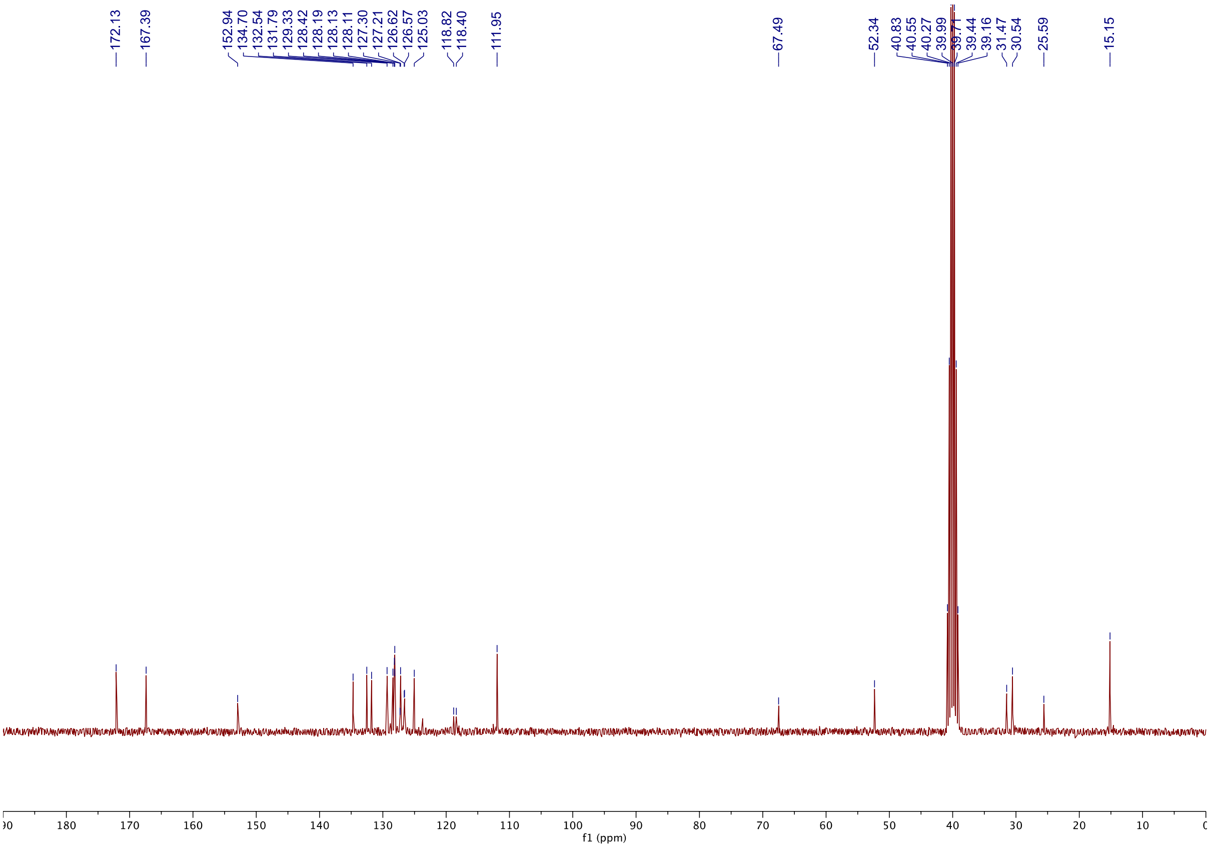
172.13
167.39
152.94
134.70
132.54
131.79
129.33
128.42
128.19
128.13
128.11
127.30
127.21
126.62
126.57
125.03
118.82
118.40
111.95
67.49
52.34
40.83
40.55
40.27
39.99
39.71
39.44
39.16
31.47
30.54
25.59
15.15
f1 (ppm)

Compound 20

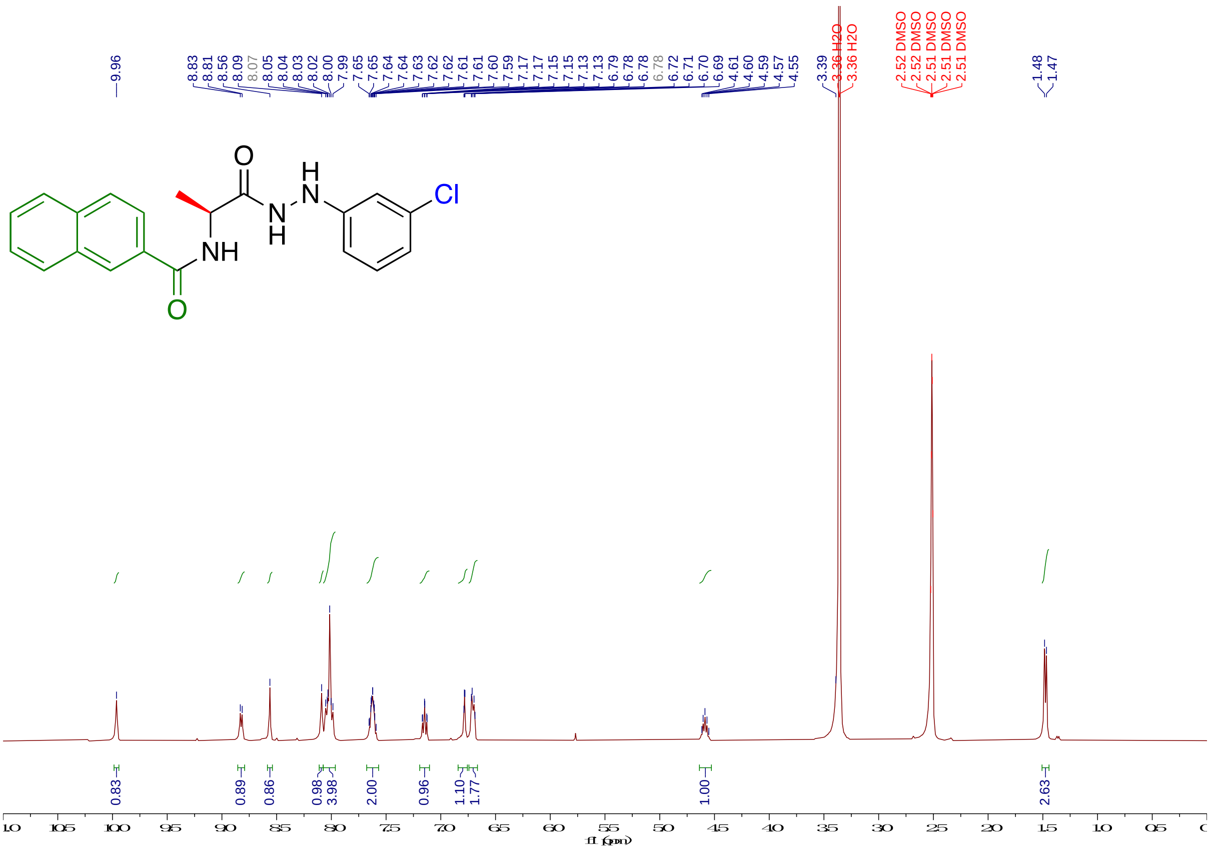
O
H
N
N
H
Cl
NH
O
9.96
8.83
8.81
8.56
8.09
8.07
8.05
8.04
8.03
8.02
8.00
7.99
7.65
7.65
7.64
7.64
7.63
7.62
7.62
7.61
7.61
7.60
7.59
7.17
7.17
7.15
7.15
7.13
6.79
6.78
6.78
6.78
6.72
6.71
6.70
6.69
4.61
4.60
4.59
4.57
4.55
3.39
3.36 H2O
3.36 H2O
2.52 DMSO
2.52 DMSO
2.51 DMSO
2.51 DMSO
2.51 DMSO
1.48
1.47
0.83
0.89
0.86
0.98
3.98
2.00
0.96
1.10
1.77
1.00
2.63
f1 (ppm)

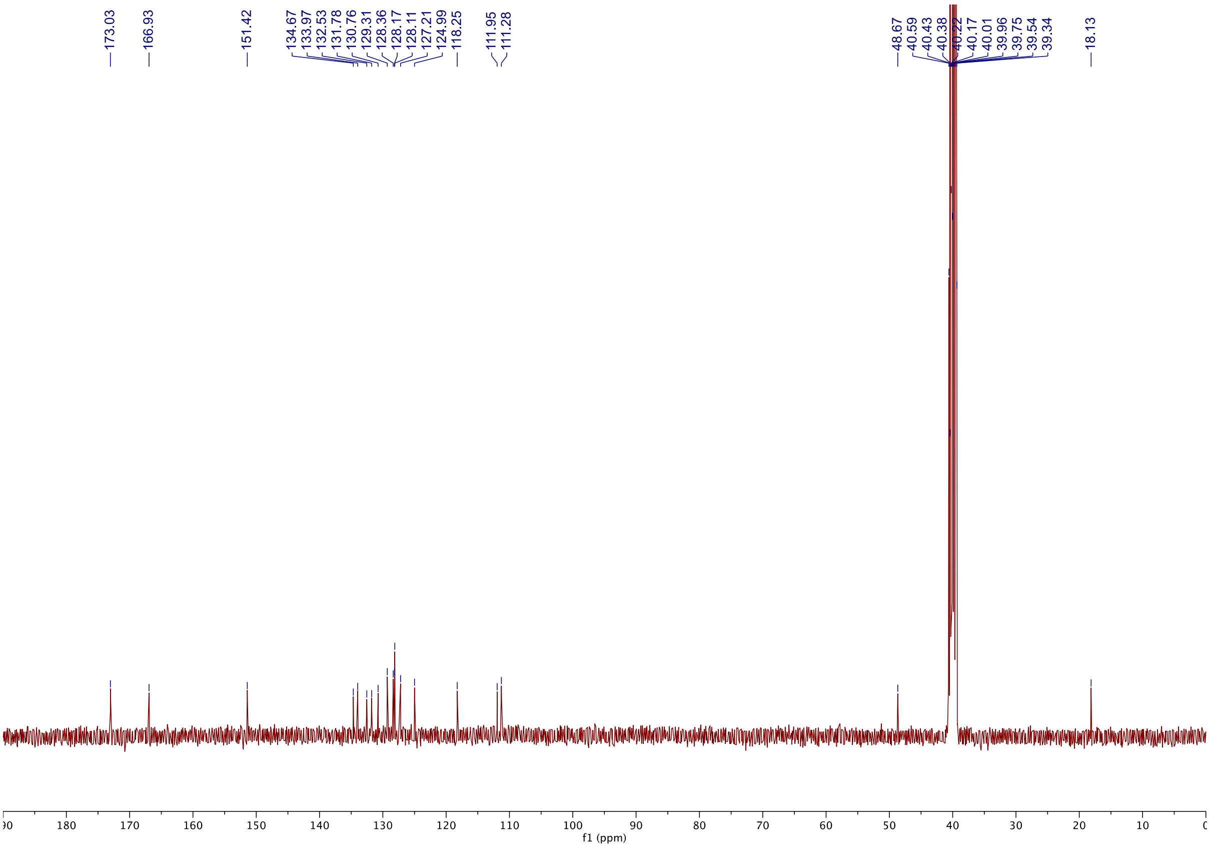
173.03
166.93
151.42
134.67
133.97
132.53
131.78
130.76
129.31
128.36
128.17
128.11
127.21
124.99
118.25
111.95
111.28
48.67
40.59
40.43
40.38
40.22
40.17
40.01
39.96
39.75
39.54
39.34
18.13
180
170
160
150
140
130
120
110
100
90
80
70
60
50
40
30
20
10
f1 (ppm)

Compound 21

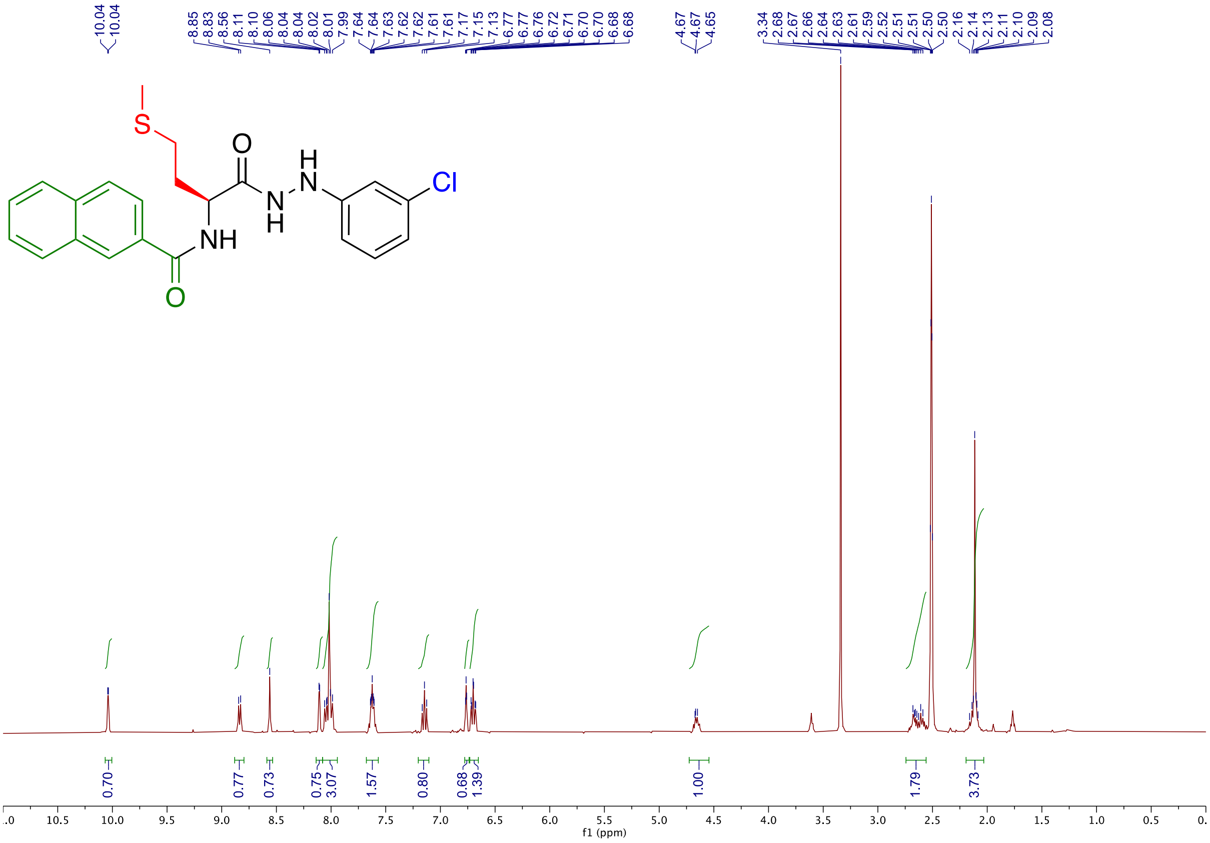

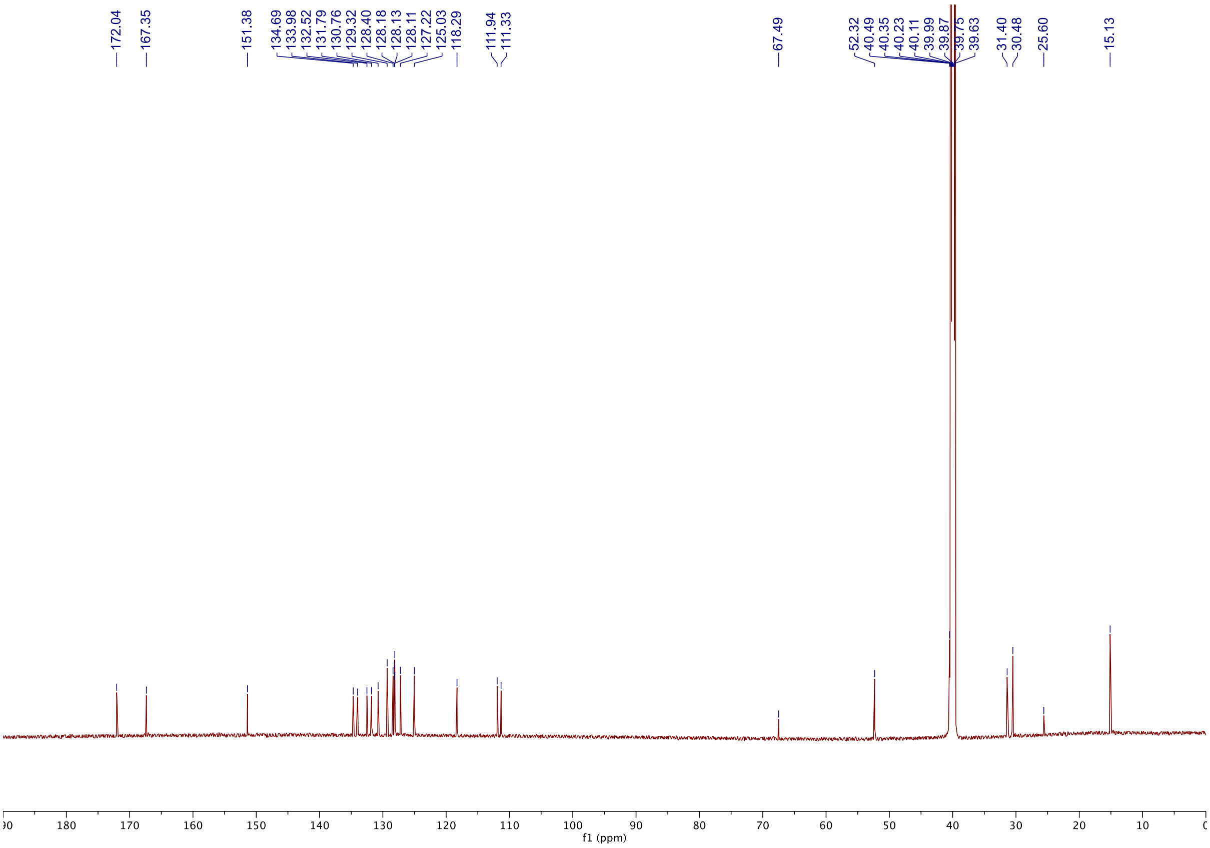

Compound 22

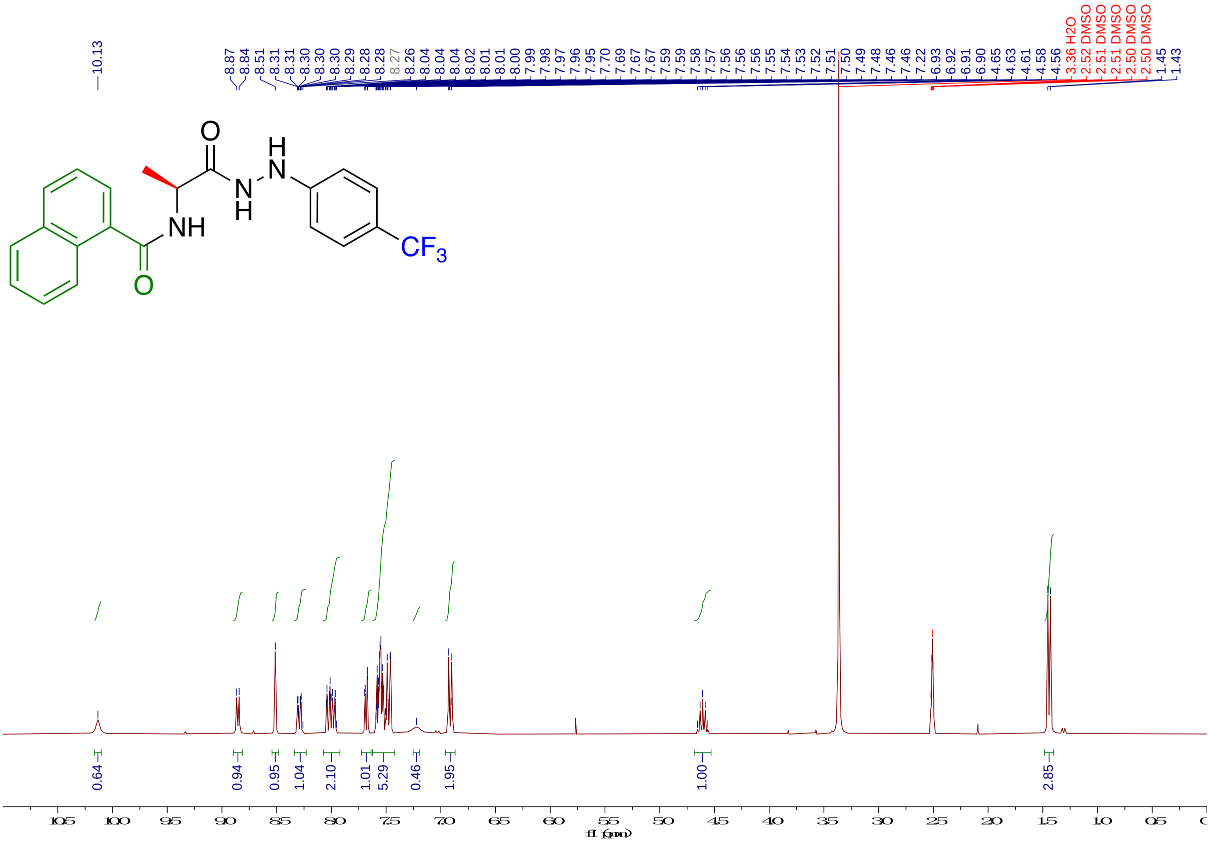

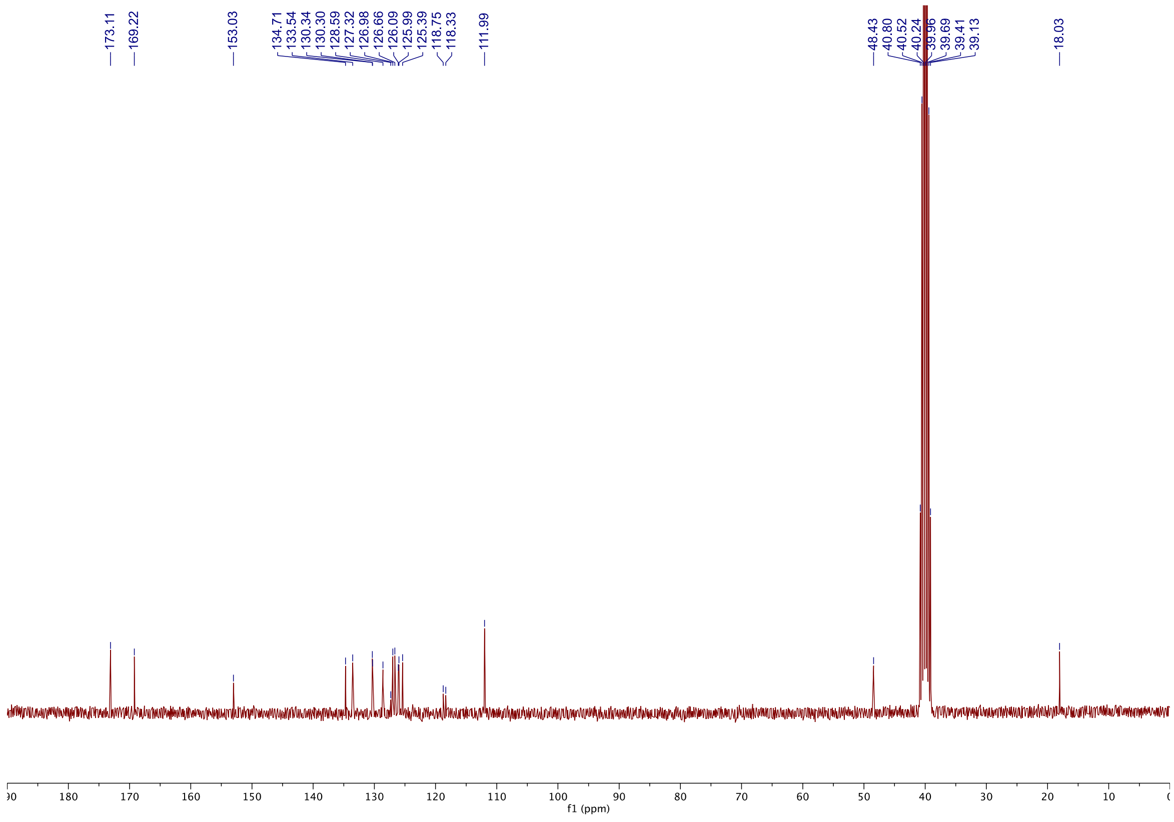

Compound 23

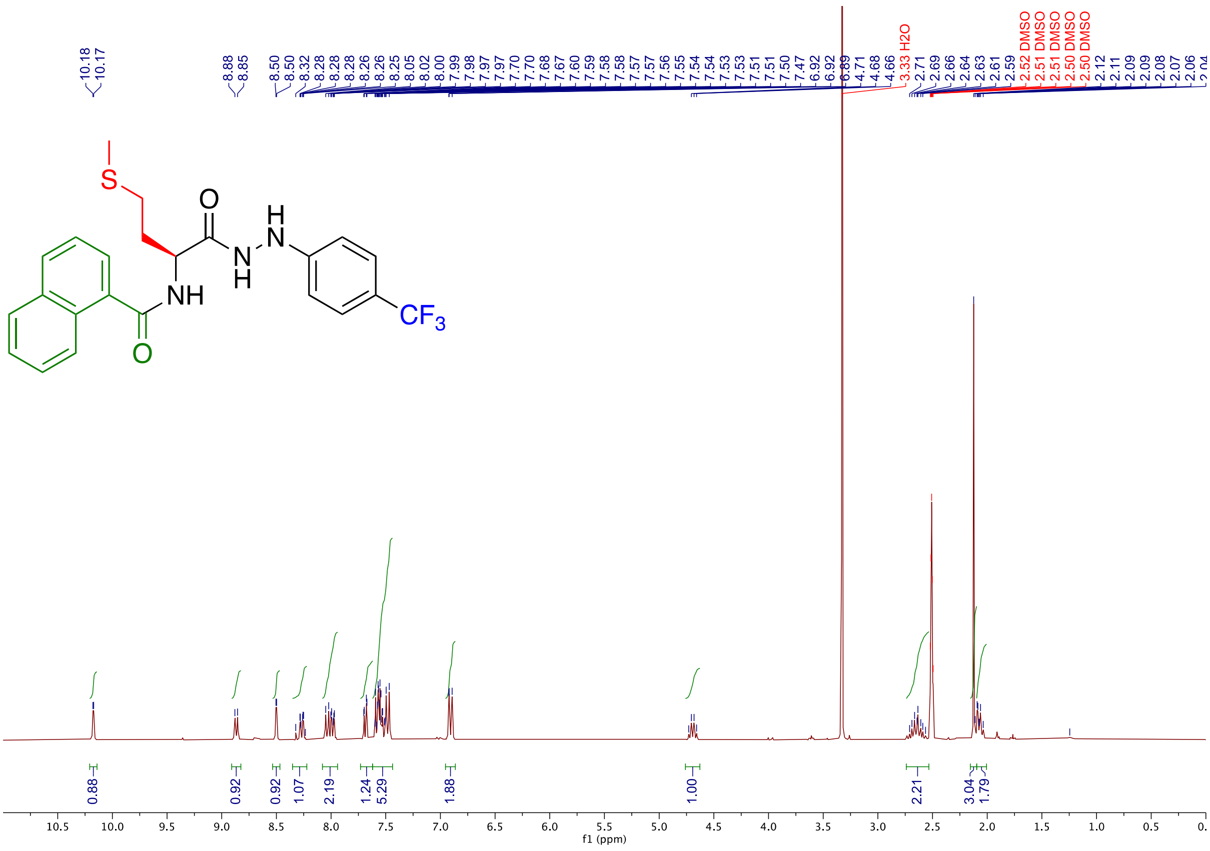

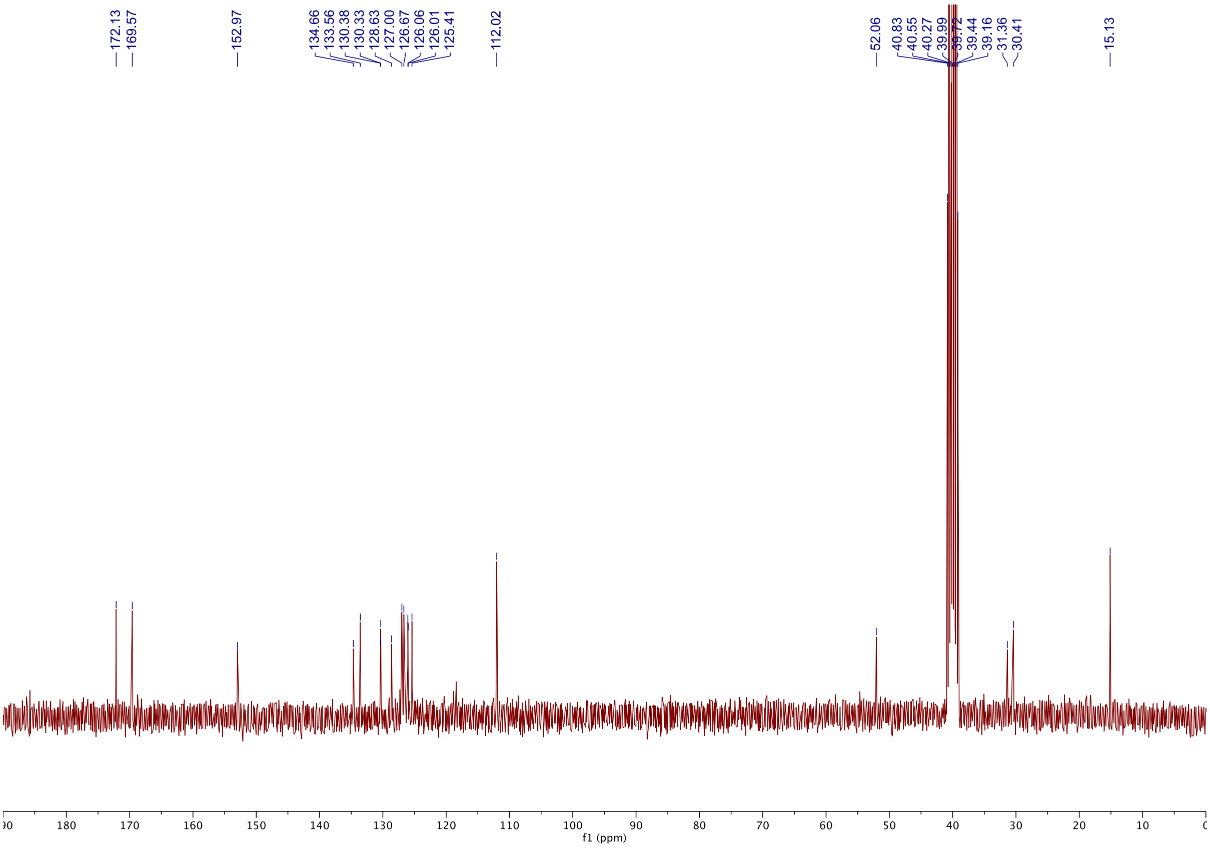

Compound 24

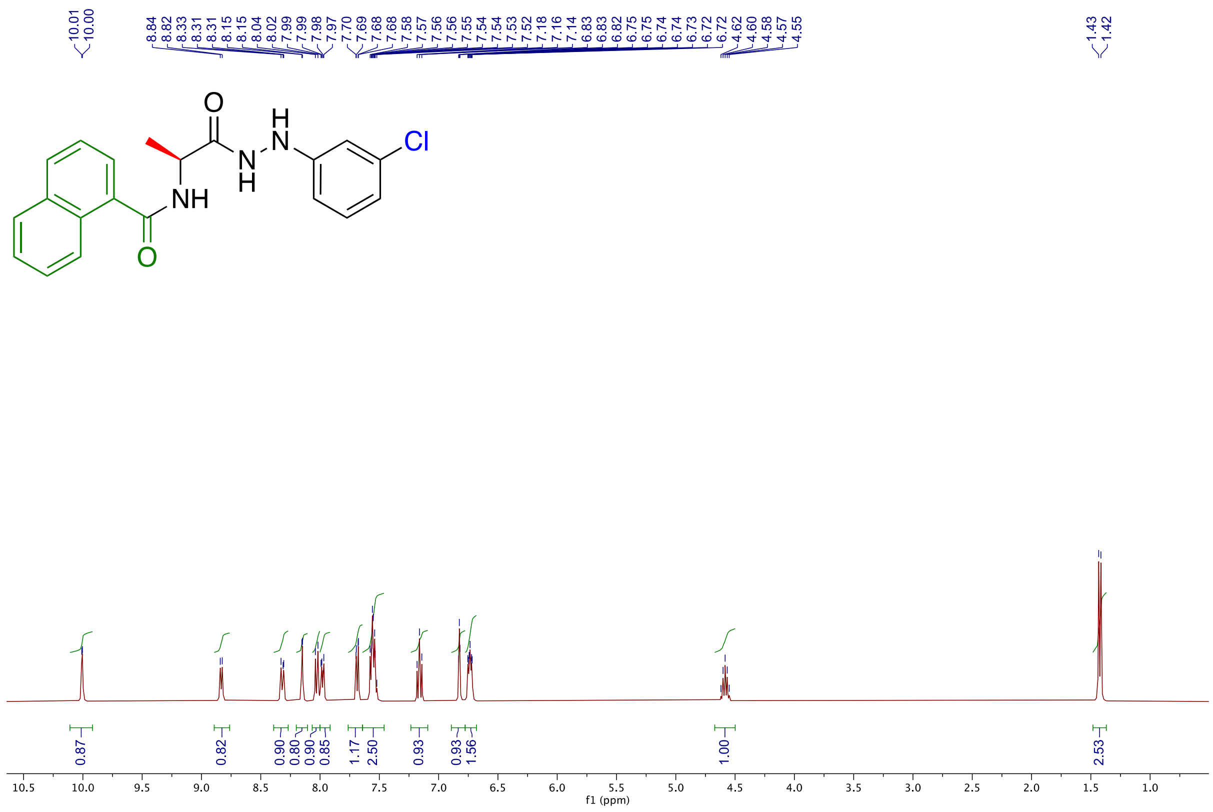

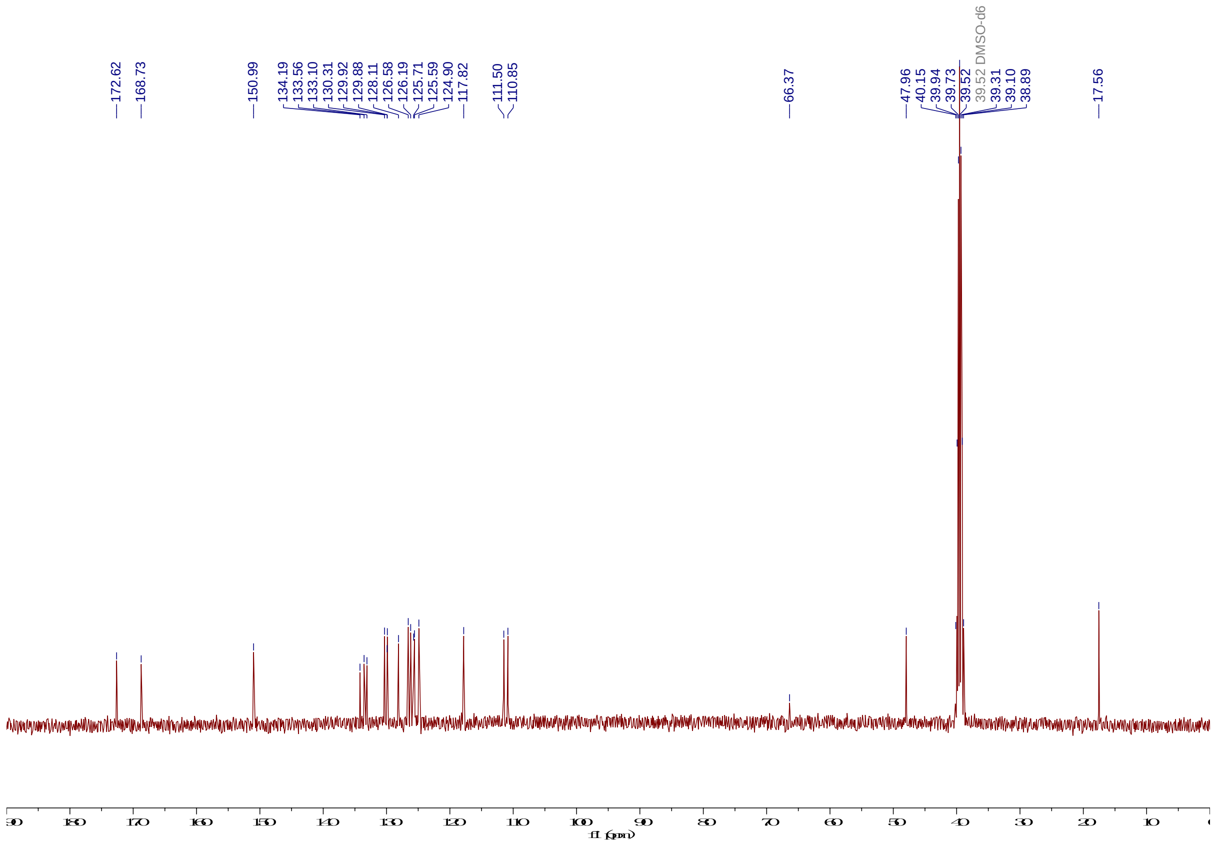

Compound 25

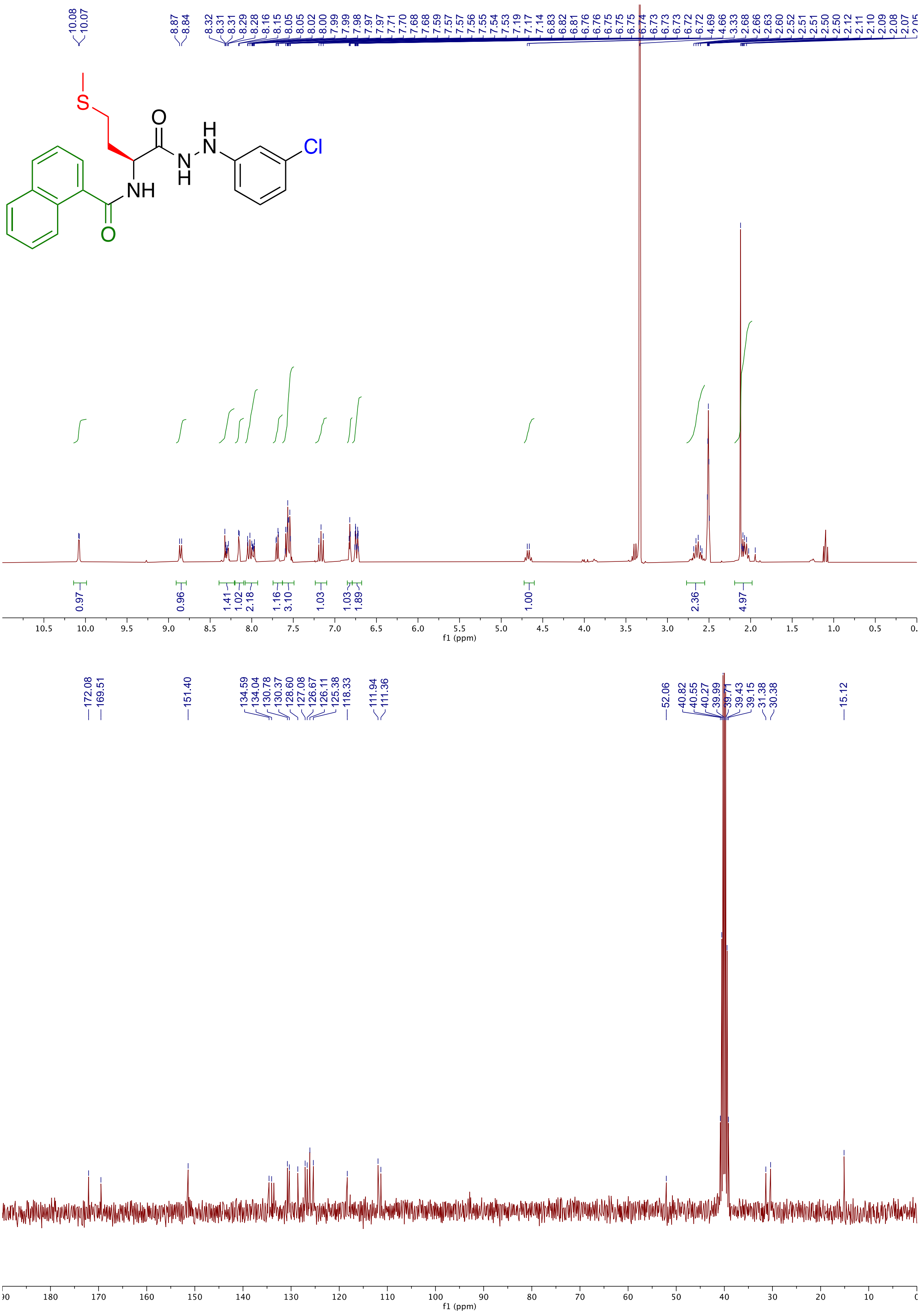

Compound 26

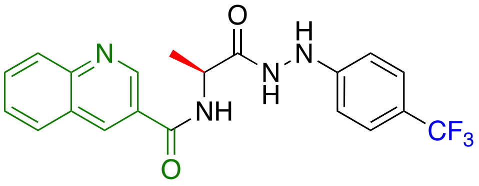

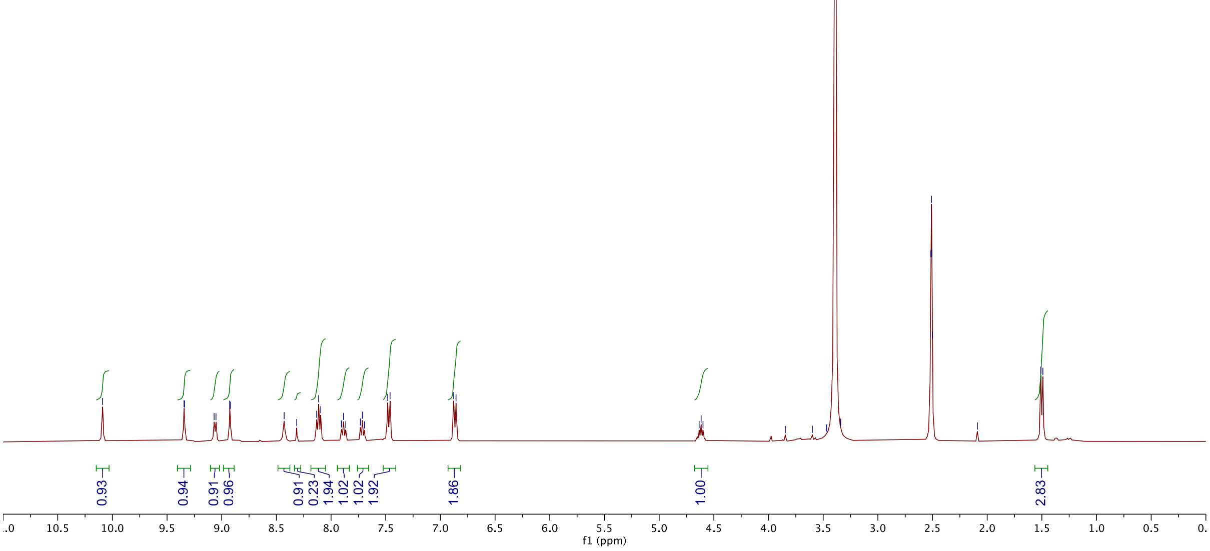

10.09
9.35
9.34
9.07
9.05
8.93
8.92
8.43
8.31
8.13
8.11
8.09
7.90
7.89
7.87
7.73
7.71
7.69
7.48
7.46
6.88
6.86
4.63
4.62
4.60
3.85
3.60
3.47
3.39
3.34
2.52
2.51
2.50
2.50
2.09
1.51
1.49
0.93
0.94
0.91
0.96
0.91
0.23
1.94
1.02
1.02
1.92
1.86
1.00
2.83
f1 (ppm)

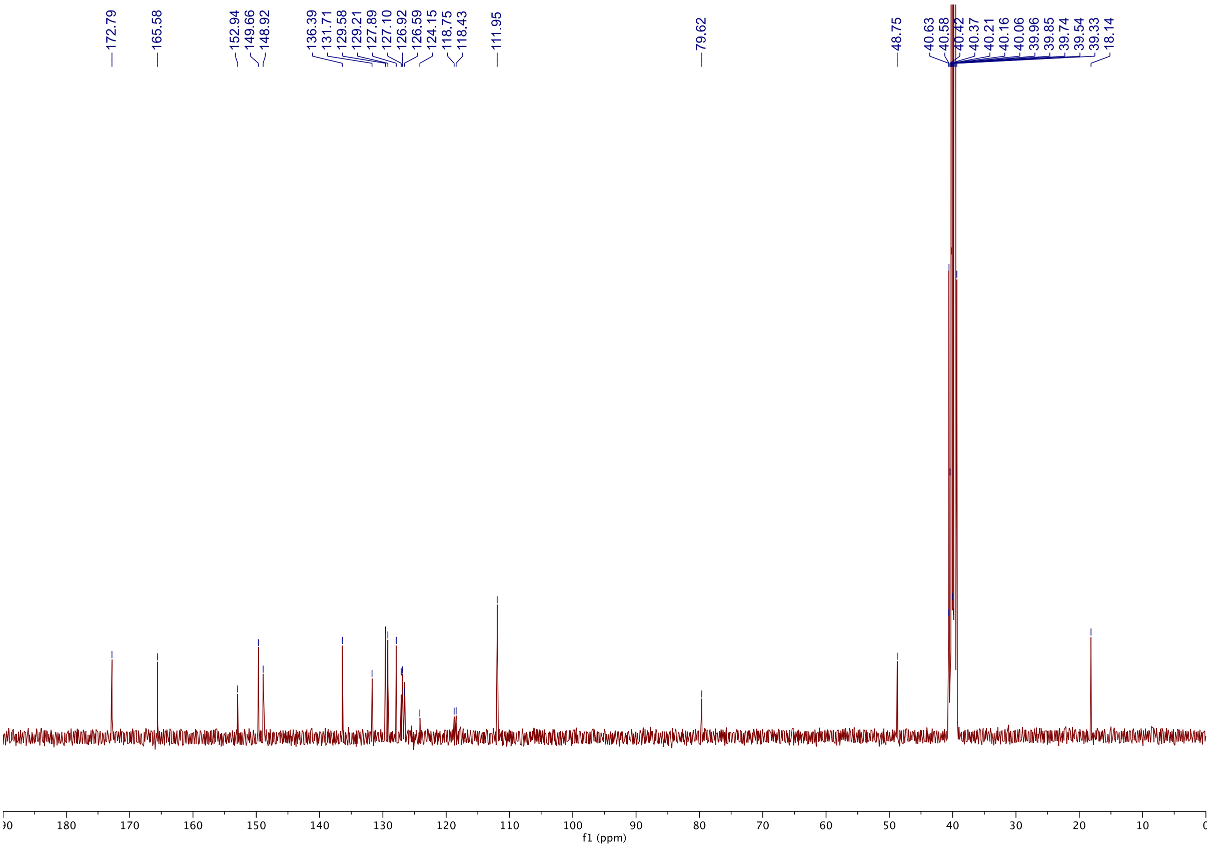

172.79
165.58
152.94
149.66
148.92
136.39
131.71
129.58
129.21
127.89
127.10
126.92
126.59
124.15
118.75
118.43
111.95
79.62
48.75
40.63
40.58
40.42
40.37
40.21
40.16
40.06
39.96
39.85
39.74
39.54
39.33
18.14
f1 (ppm)

Compound 27

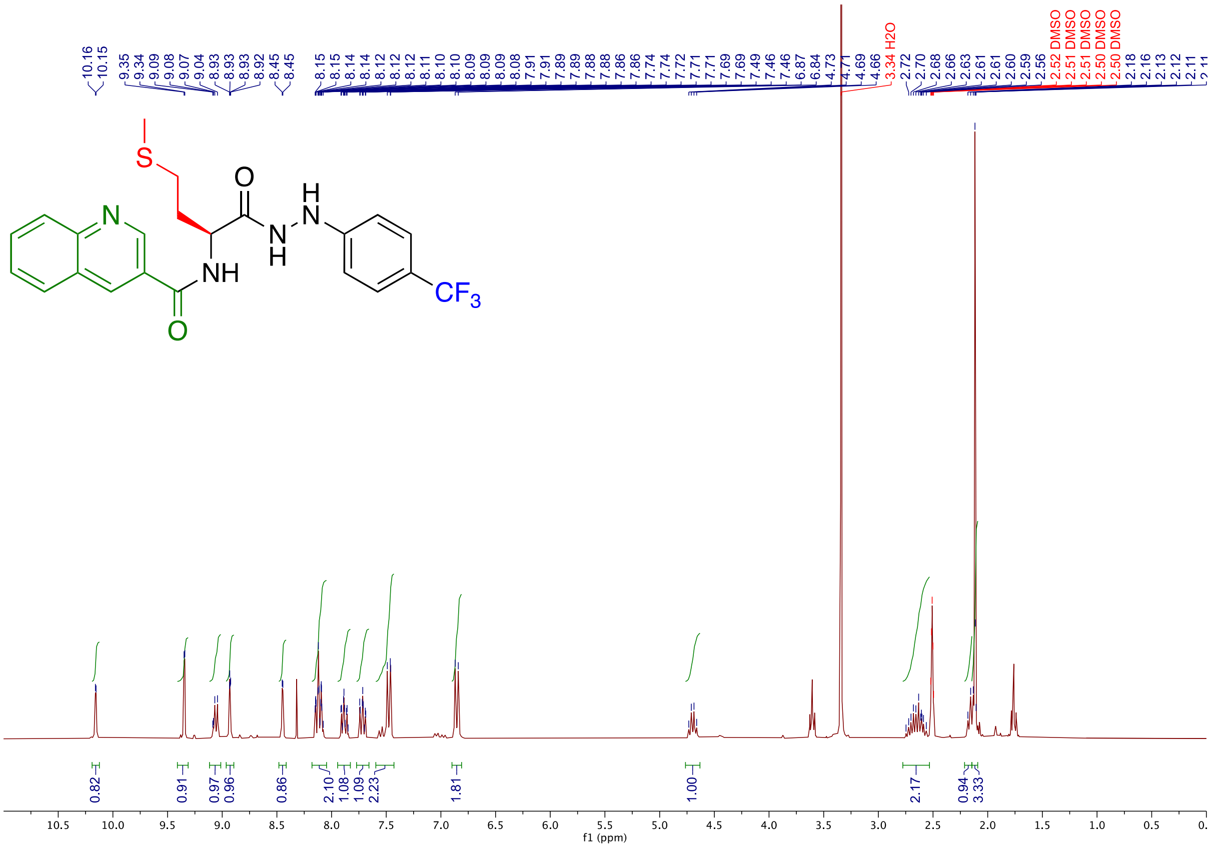
S
O
H
N
N
H
N
NH
O
CF3
10.16
10.15
9.35
9.34
9.09
9.08
9.07
9.04
8.93
8.93
8.92
8.45
8.45
8.15
8.15
8.14
8.14
8.12
8.12
8.12
8.11
8.10
8.10
8.09
8.09
8.09
8.08
7.91
7.91
7.89
7.89
7.88
7.88
7.86
7.74
7.74
7.72
7.71
7.71
7.69
7.69
7.49
7.46
7.46
6.87
6.84
4.73
4.71
4.69
4.66
3.34 H2O
2.72
2.70
2.68
2.66
2.63
2.61
2.61
2.60
2.59
2.56
2.52 DMSO
2.51 DMSO
2.51 DMSO
2.50 DMSO
2.50 DMSO
2.18
2.16
2.13
2.12
2.11
0.82
0.91
0.97
0.96
0.86
2.10
1.08
1.09
2.23
1.81
1.00
2.17
0.94
3.33
10.5
10.0
9.5
9.0
8.5
8.0
7.5
7.0
6.5
6.0
5.5
5.0
4.5
4.0
3.5
3.0
2.5
2.0
1.5
1.0
0.5
f1 (ppm)

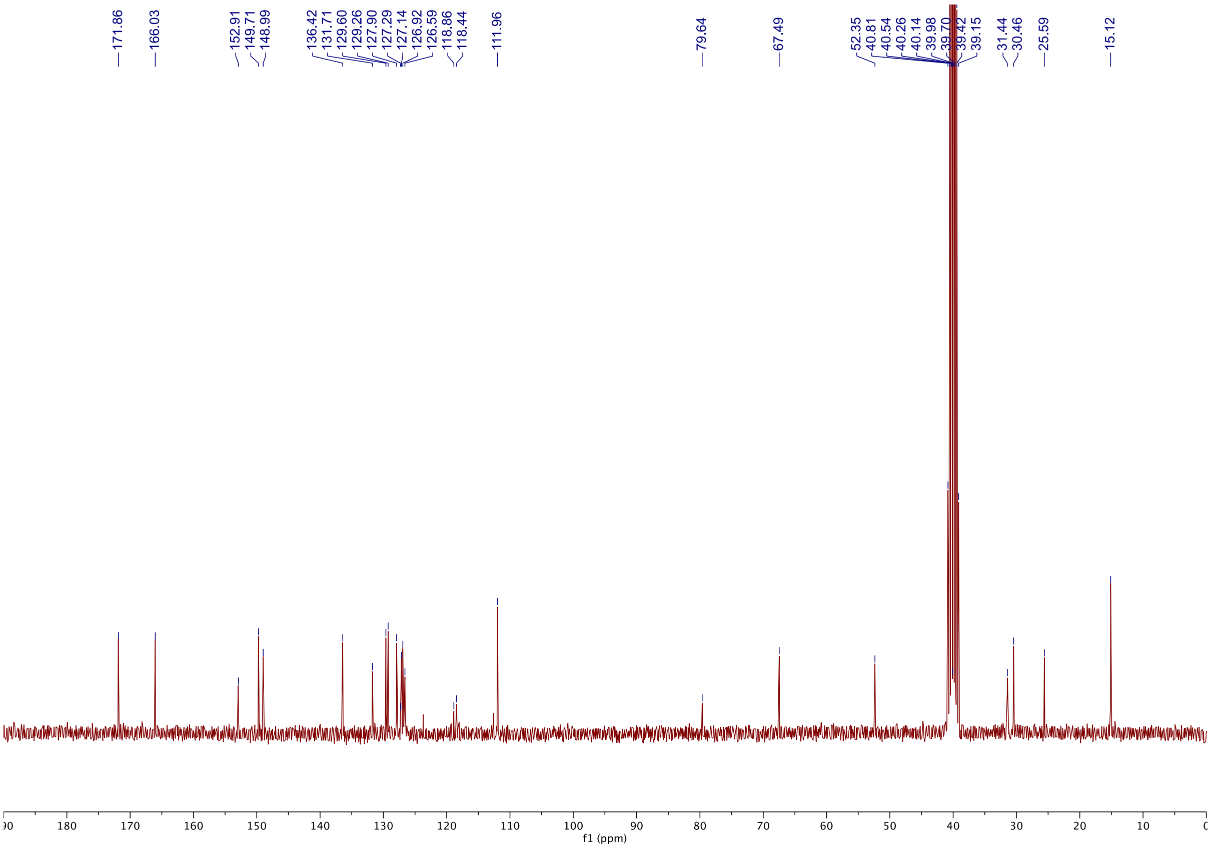
171.86
166.03
152.91
149.71
148.99
136.42
131.71
129.60
129.26
127.90
127.29
127.14
126.92
126.59
118.86
118.44
111.96
79.64
67.49
52.35
40.81
40.54
40.26
40.14
39.98
39.70
39.42
39.15
31.44
30.46
25.59
15.12
180
170
160
150
140
130
120
110
100
90
80
70
60
50
40
30
20
10
f1 (ppm)

Compound 28

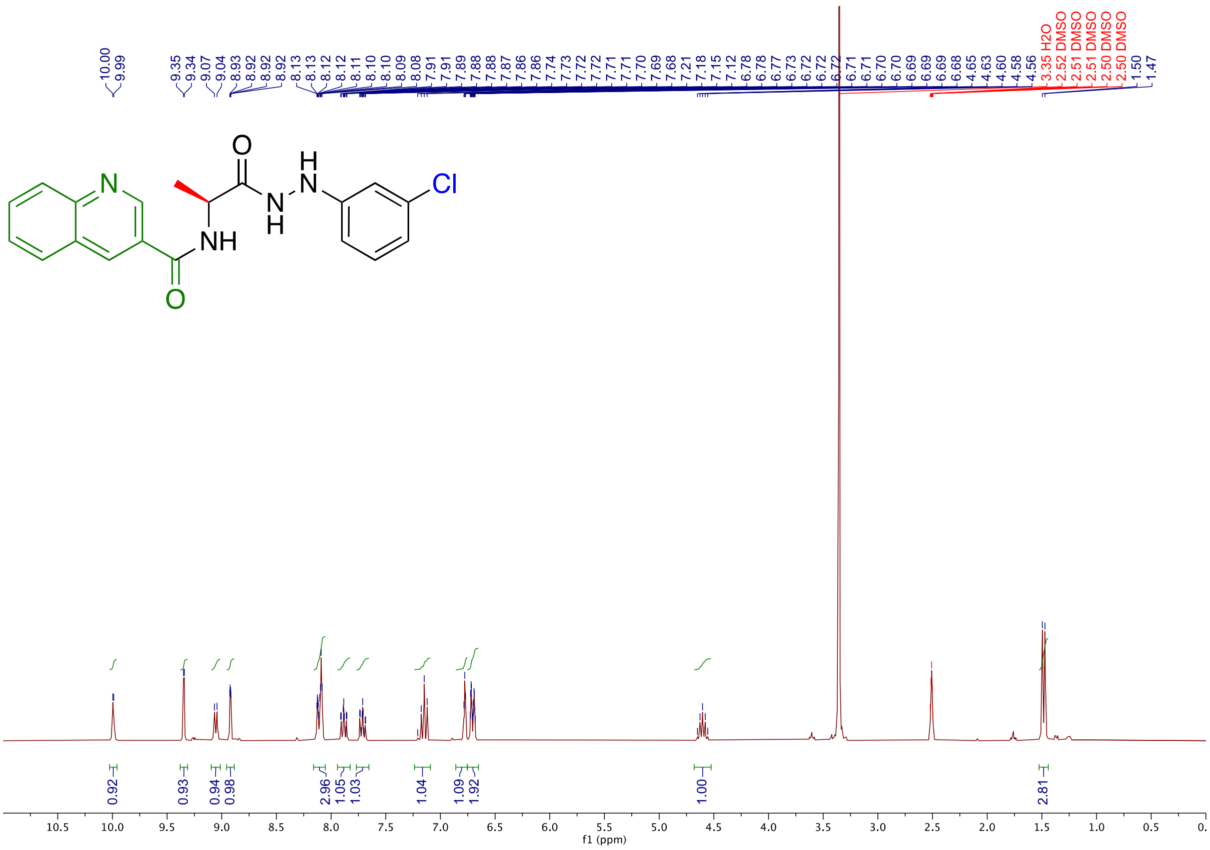
10.00
9.99
9.35
9.34
9.07
9.04
8.93
8.92
8.92
8.92
8.13
8.13
8.12
8.12
8.11
8.10
8.10
8.09
8.08
7.91
7.89
7.88
7.87
7.86
7.86
7.74
7.73
7.72
7.72
7.71
7.71
7.70
7.69
7.68
7.21
7.18
7.15
7.12
6.78
6.78
6.77
6.73
6.72
6.72
6.72
6.71
6.71
6.70
6.70
6.69
6.69
6.69
6.68
4.65
4.63
4.60
4.58
4.56
3.35 H2O
2.52 DMSO
2.51 DMSO
2.51 DMSO
2.50 DMSO
2.50 DMSO
1.50
1.47
N
O
H
N
N
H
Cl
NH
O
0.92
0.93
0.94
0.98
2.96
1.05
1.03
1.04
1.09
1.92
1.00
2.81
f1 (ppm)

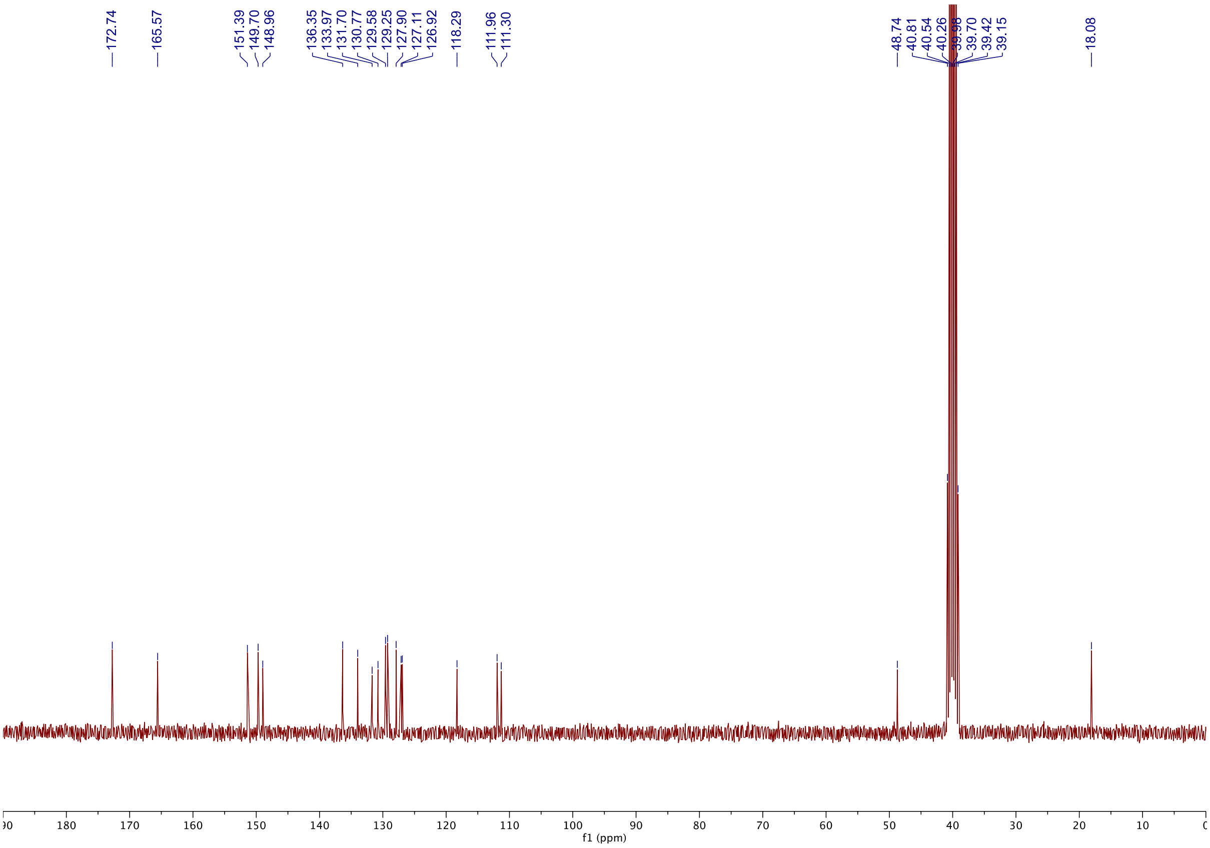
172.74
165.57
151.39
149.70
148.96
136.35
133.97
131.70
130.77
129.58
129.25
127.90
127.11
126.92
118.29
111.96
111.30
48.74
40.81
40.54
40.26
39.98
39.70
39.42
39.15
18.08
f1 (ppm)

Compound 29

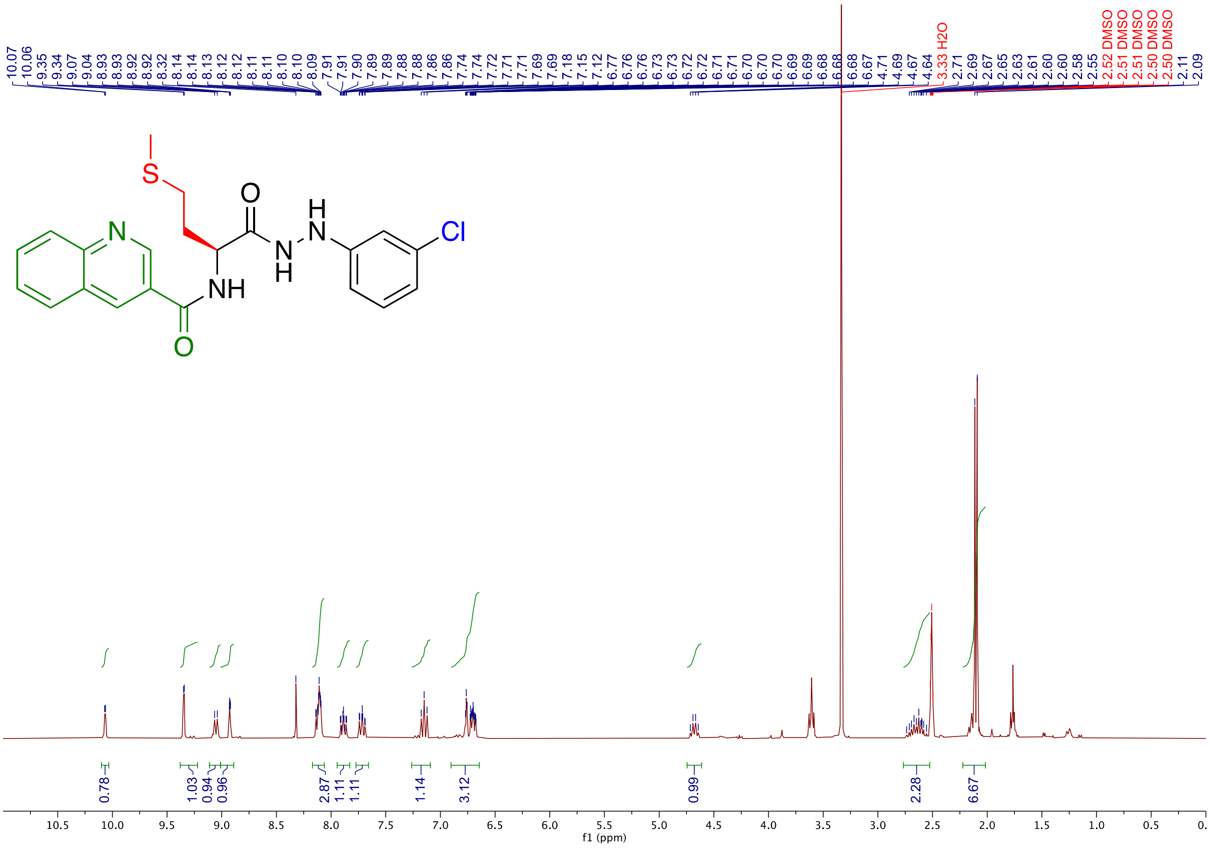

10.07
10.06
9.35
9.34
9.07
9.04
8.93
8.93
8.92
8.92
8.32
8.14
8.14
8.13
8.12
8.12
8.11
8.11
8.10
8.09
7.91
7.90
7.89
7.89
7.88
7.88
7.86
7.86
7.74
7.74
7.72
7.71
7.71
7.69
7.69
7.18
7.15
7.12
6.77
6.76
6.76
6.73
6.73
6.72
6.72
6.71
6.71
6.70
6.70
6.69
6.69
6.68
6.68
6.68
6.67
4.71
4.69
4.67
4.64
3.33 H2O
2.71
2.69
2.67
2.65
2.63
2.61
2.60
2.60
2.58
2.55
2.52 DMSO
2.51 DMSO
2.51 DMSO
2.50 DMSO
2.50 DMSO
2.11
2.09
S
O
H
N
N
H
Cl
N
NH
O
0.78
1.03
0.94
0.96
2.87
1.11
1.11
1.14
3.12
0.99
2.28
6.67
f1 (ppm)

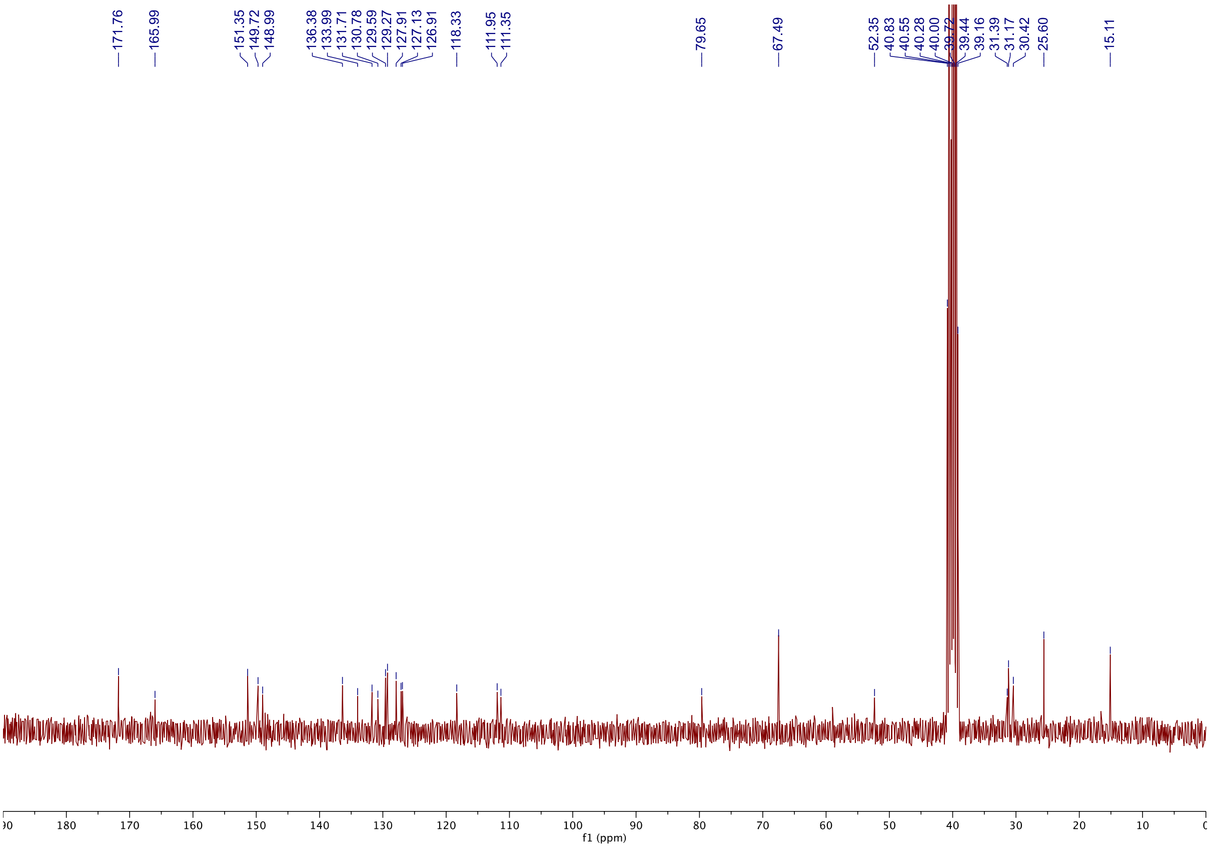

171.76
165.99
151.35
149.72
148.99
136.38
133.99
131.71
130.78
129.59
129.27
127.91
127.13
126.91
118.33
111.95
111.35
79.65
67.49
52.35
40.83
40.55
40.28
40.00
39.72
39.44
39.16
31.39
31.17
30.42
25.60
15.11
f1 (ppm)

Compound 30

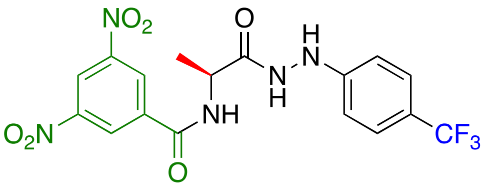

NO2
O2N
O
NH
H
N
N
H
O
CF3

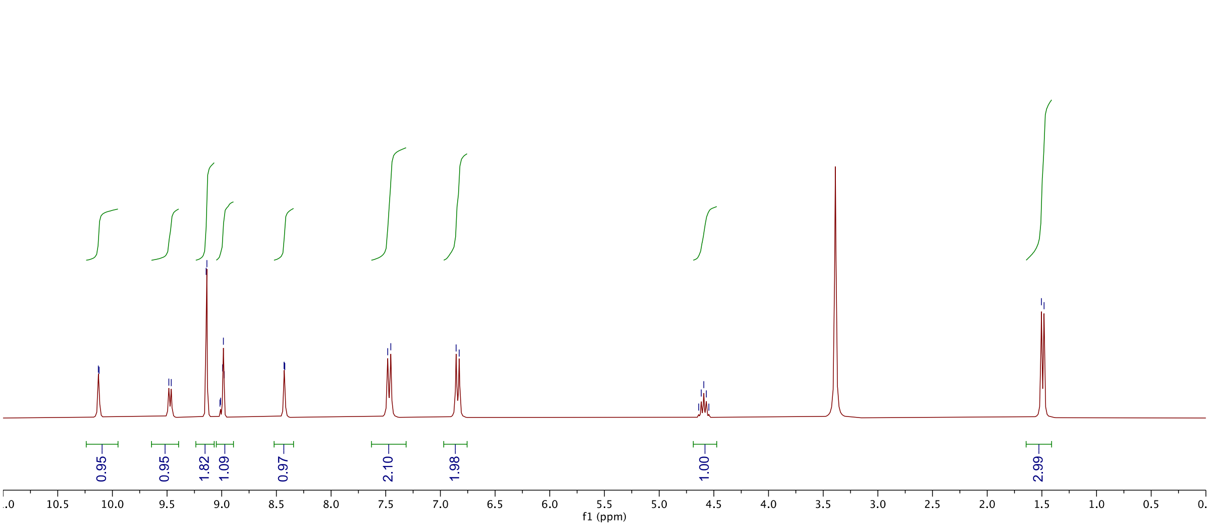

10.13
10.12
9.48
9.46
9.14
9.13
9.02
9.01
8.99
8.99
8.98
8.43
8.43
8.42
7.48
7.45
6.86
6.83
4.64
4.62
4.59
4.57
4.54
3.39
1.50
1.48
0.95
0.95
1.82
1.09
0.97
2.10
1.98
1.00
2.99
f1 (ppm)

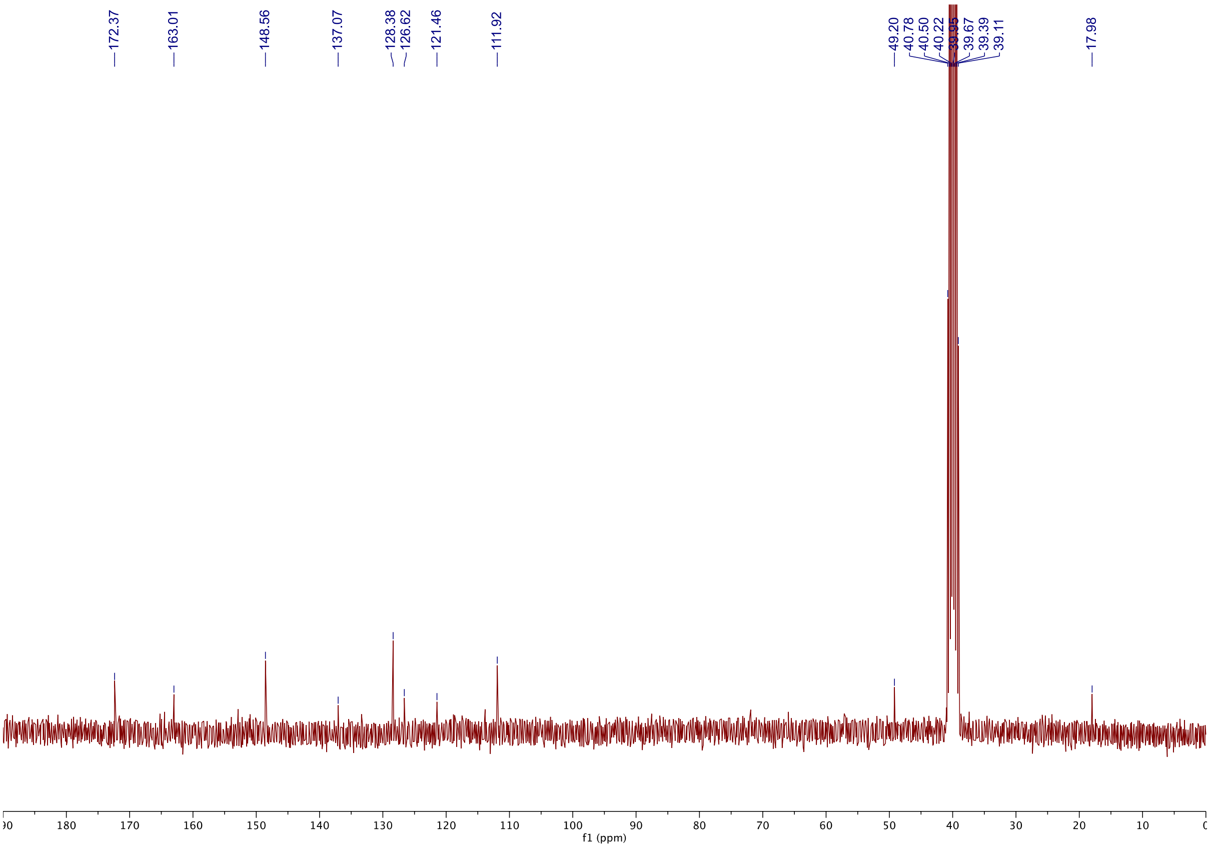

172.37
163.01
148.56
137.07
128.38
126.62
121.46
111.92
49.20
40.78
40.50
40.22
39.67
39.39
39.11
17.98
f1 (ppm)

Compound 31

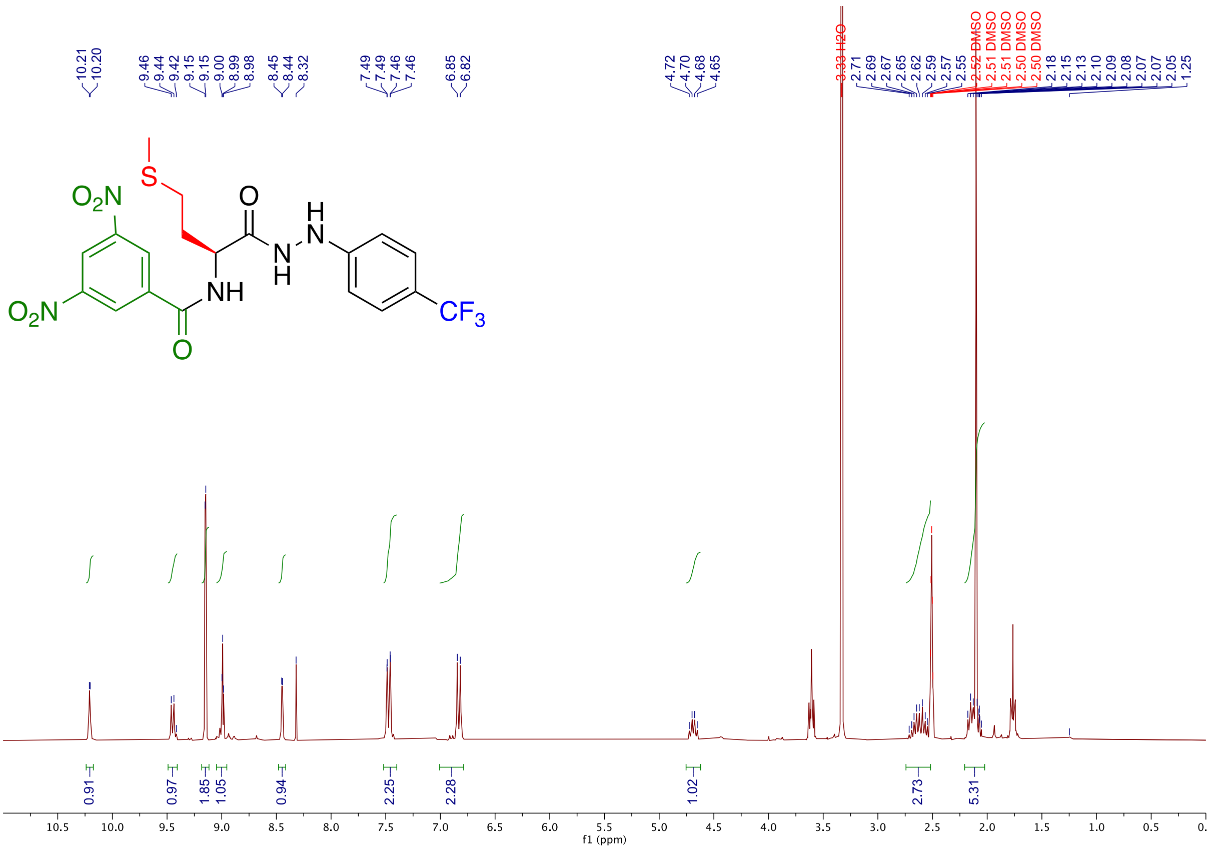
10.21
10.20
9.46
9.44
9.42
9.15
9.00
8.99
8.98
8.45
8.44
8.32
7.49
7.49
7.46
7.46
6.85
6.82
4.72
4.70
4.68
4.65
3.33 H2O
2.71
2.69
2.67
2.65
2.62
2.59
2.57
2.55
2.52 DMSO
2.51 DMSO
2.51 DMSO
2.50 DMSO
2.50 DMSO
2.18
2.15
2.13
2.10
2.09
2.08
2.07
2.07
2.05
1.25
0.91
0.97
1.85
1.05
0.94
2.25
2.28
1.02
2.73
5.31
f1 (ppm)

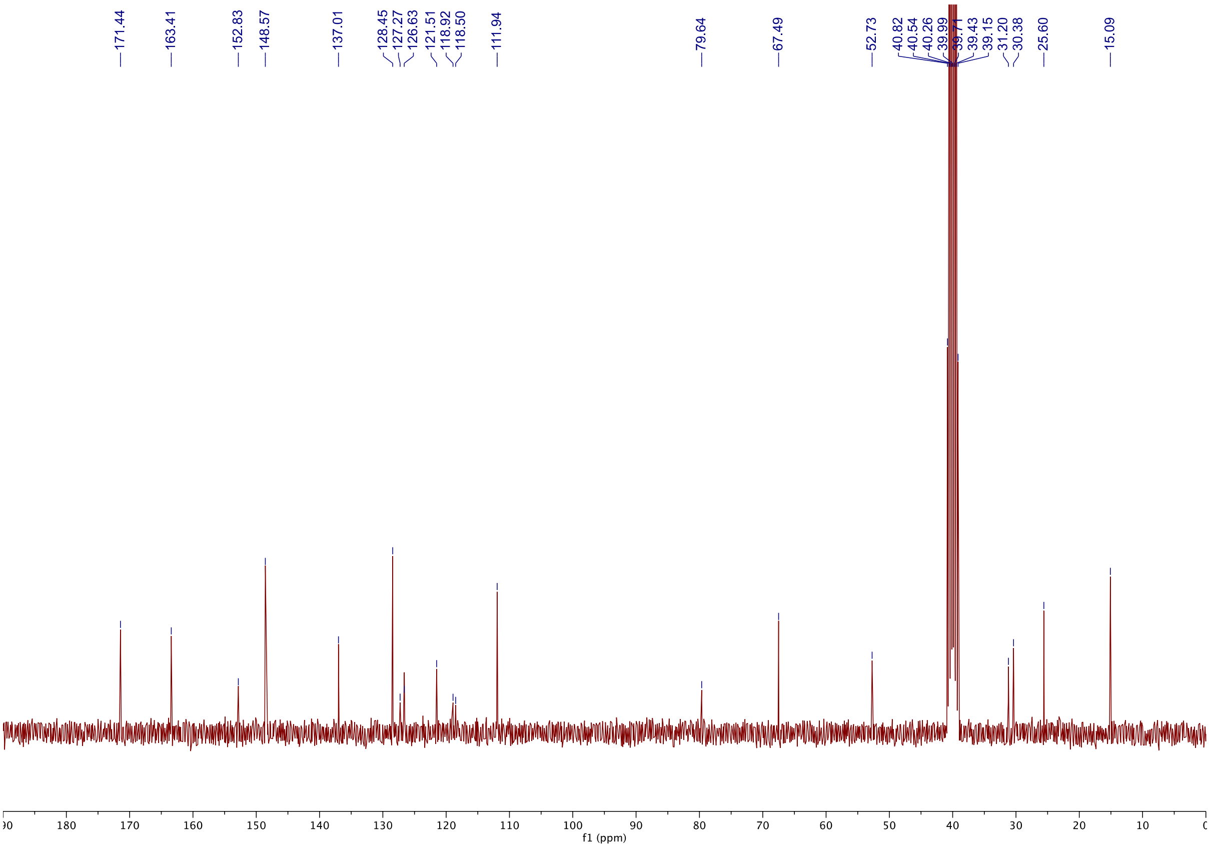
171.44
163.41
152.83
148.57
137.01
128.45
127.27
126.63
121.51
118.92
118.50
111.94
79.64
67.49
52.73
40.82
40.54
40.26
39.99
39.71
39.43
39.15
31.20
30.38
25.60
15.09
f1 (ppm)

Compound 32

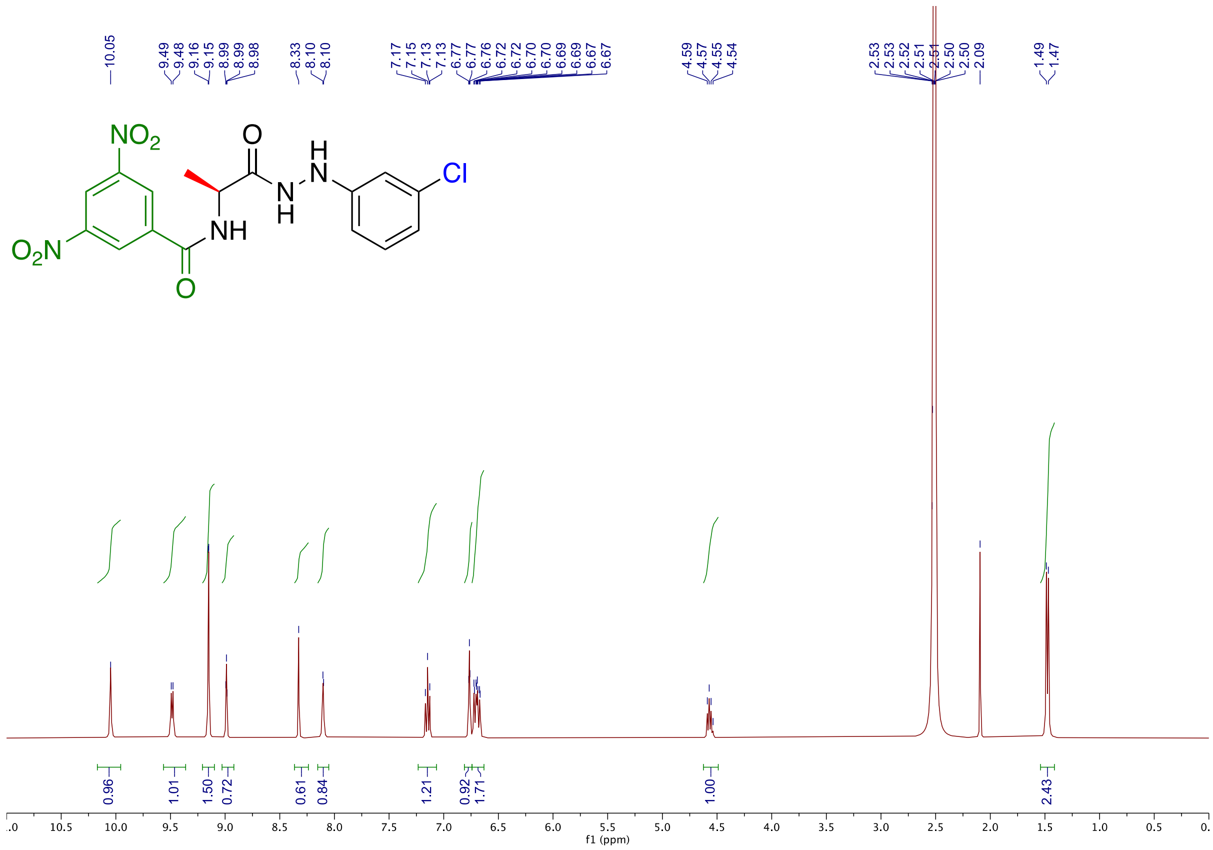

NO_2
O_2N
O
O
NH
H
N
N
H
Cl
10.05
9.49
9.48
9.16
9.15
8.99
8.99
8.98
8.33
8.10
8.10
7.17
7.15
7.13
7.13
6.77
6.76
6.72
6.72
6.70
6.70
6.69
6.67
6.67
4.59
4.57
4.55
4.54
2.53
2.53
2.52
2.51
2.51
2.50
2.50
2.09
1.49
1.47
0.96
1.01
1.50
0.72
0.61
0.84
1.21
0.92
1.71
1.00
2.43
f1 (ppm)

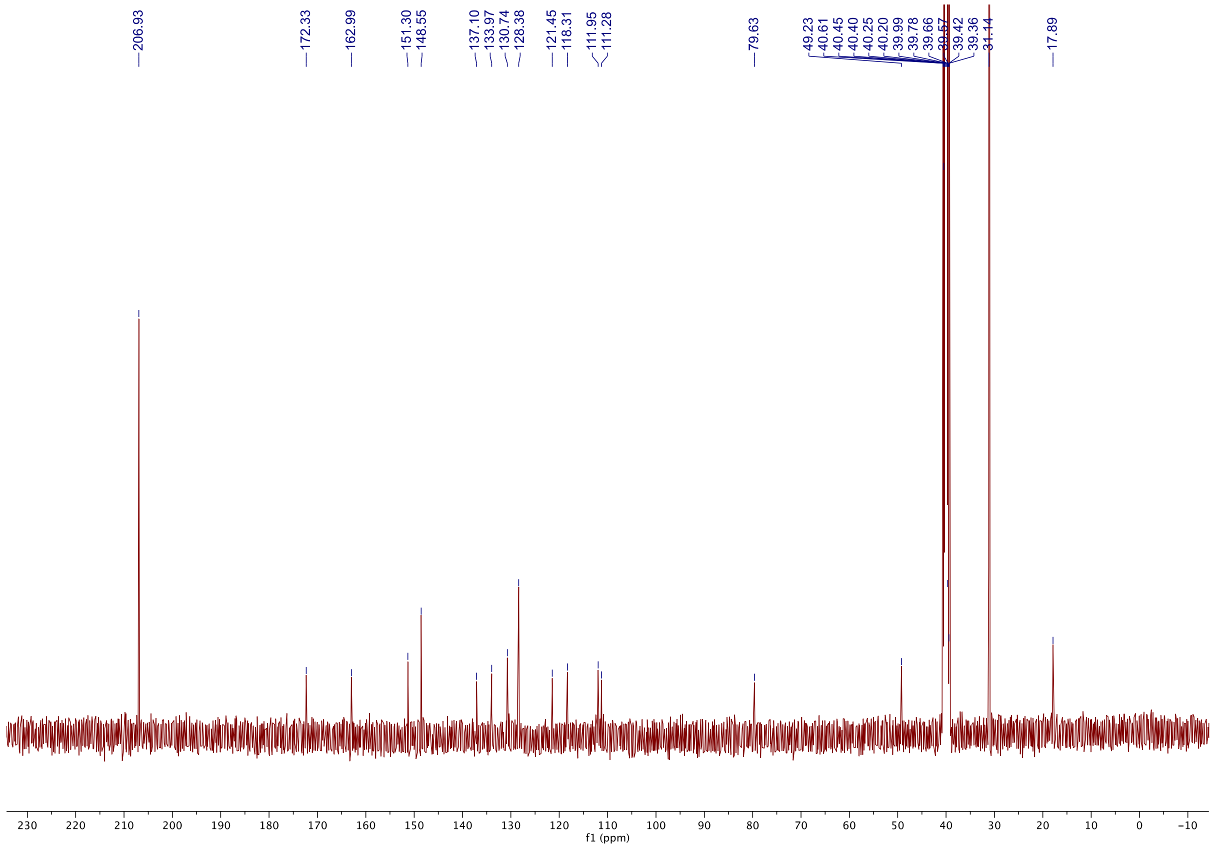

206.93
172.33
162.99
151.30
148.55
137.10
133.97
130.74
128.38
121.45
118.31
111.95
111.28
79.63
49.23
40.61
40.45
40.40
40.25
40.20
39.99
39.78
39.66
39.57
39.42
39.36
31.14
17.89
f1 (ppm)

Compound 33

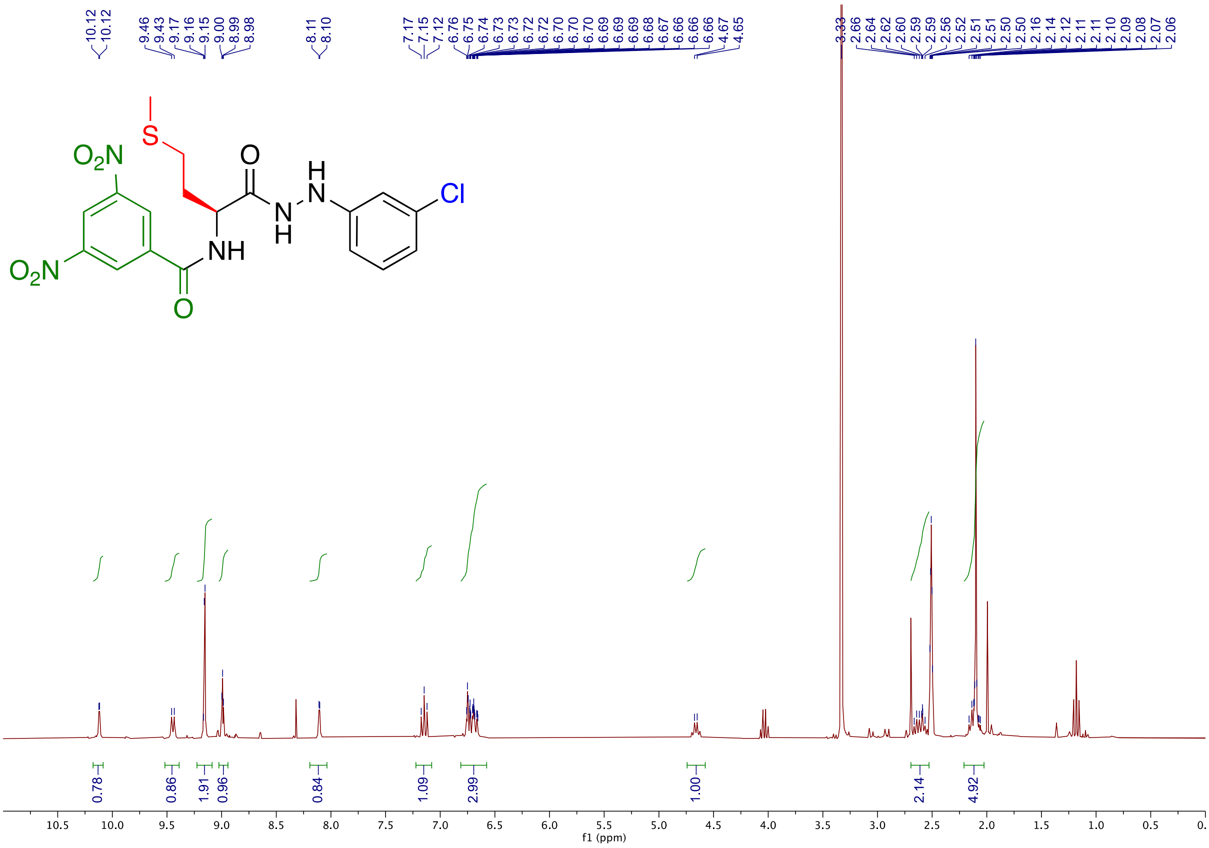
10.12
10.12
9.46
9.43
9.17
9.16
9.15
9.00
8.99
8.98
8.11
8.10
7.17
7.15
7.12
6.76
6.75
6.74
6.73
6.73
6.72
6.72
6.70
6.70
6.69
6.69
6.69
6.68
6.67
6.66
6.66
6.66
4.67
4.65
3.33
2.66
2.64
2.62
2.60
2.59
2.59
2.56
2.52
2.51
2.51
2.50
2.50
2.16
2.14
2.12
2.11
2.10
2.09
2.08
2.07
2.06
O2N
O2N
S
O
O
NH
H
N
N
H
Cl
0.78
0.86
1.91
0.96
0.84
1.09
2.99
1.00
2.14
4.92
f1 (ppm)

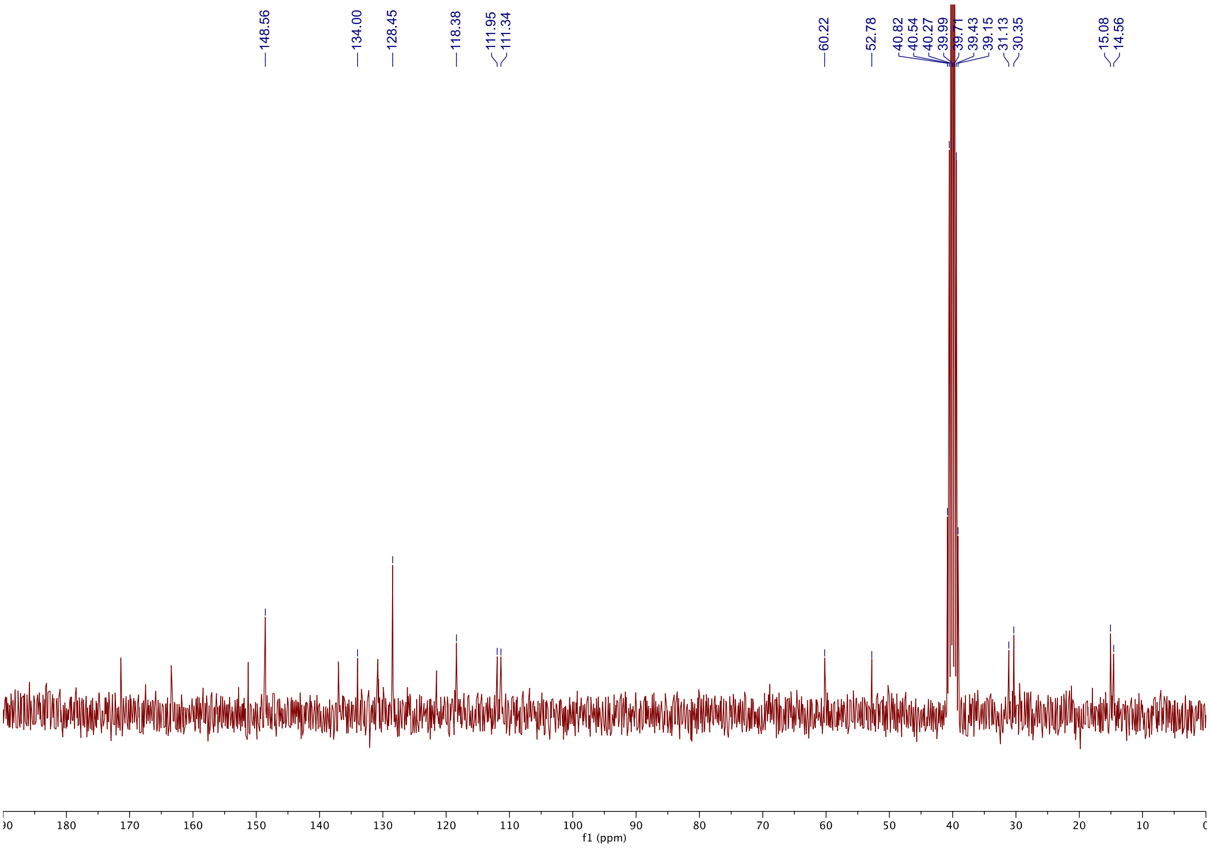
148.56
134.00
128.45
118.38
111.95
111.34
60.22
52.78
40.82
40.54
40.27
39.99
39.71
39.43
39.15
31.13
30.35
15.08
14.56
f1 (ppm)

Compound 34

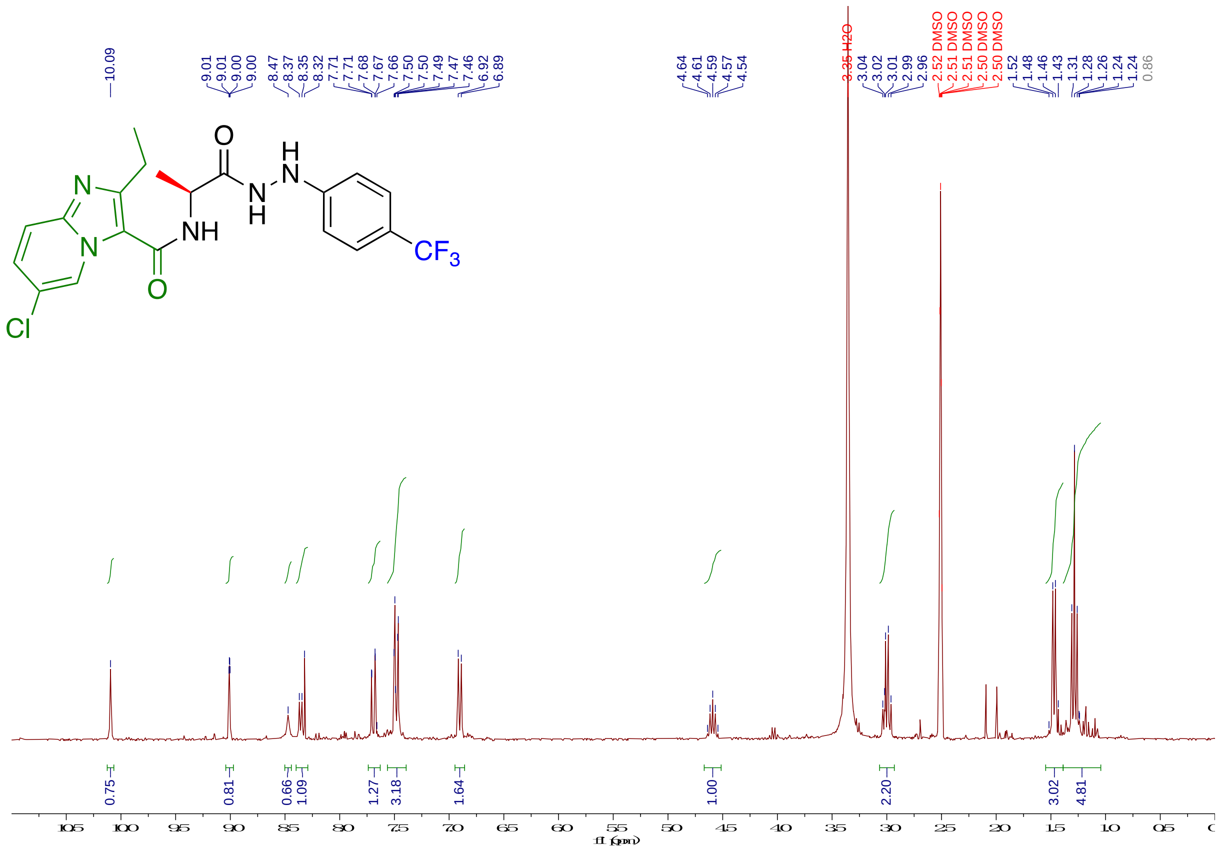

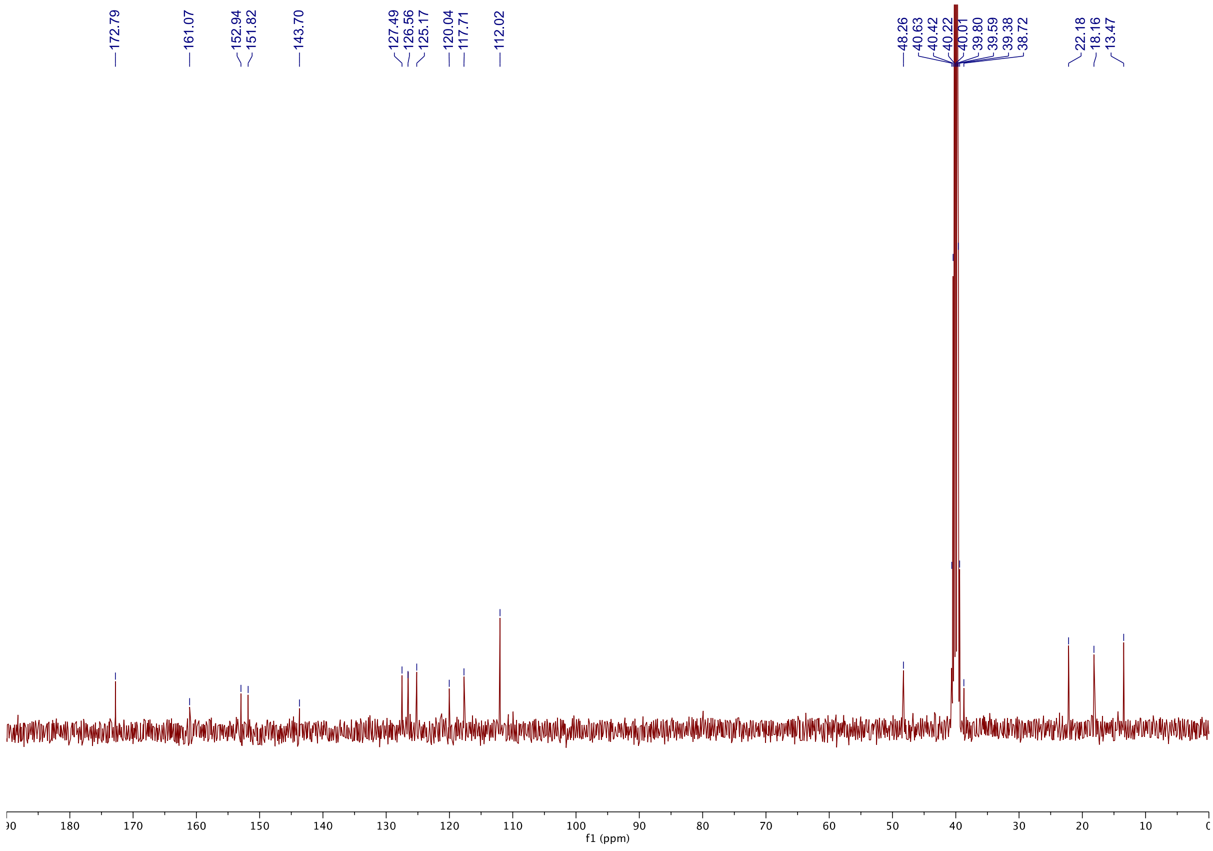

Compound 35

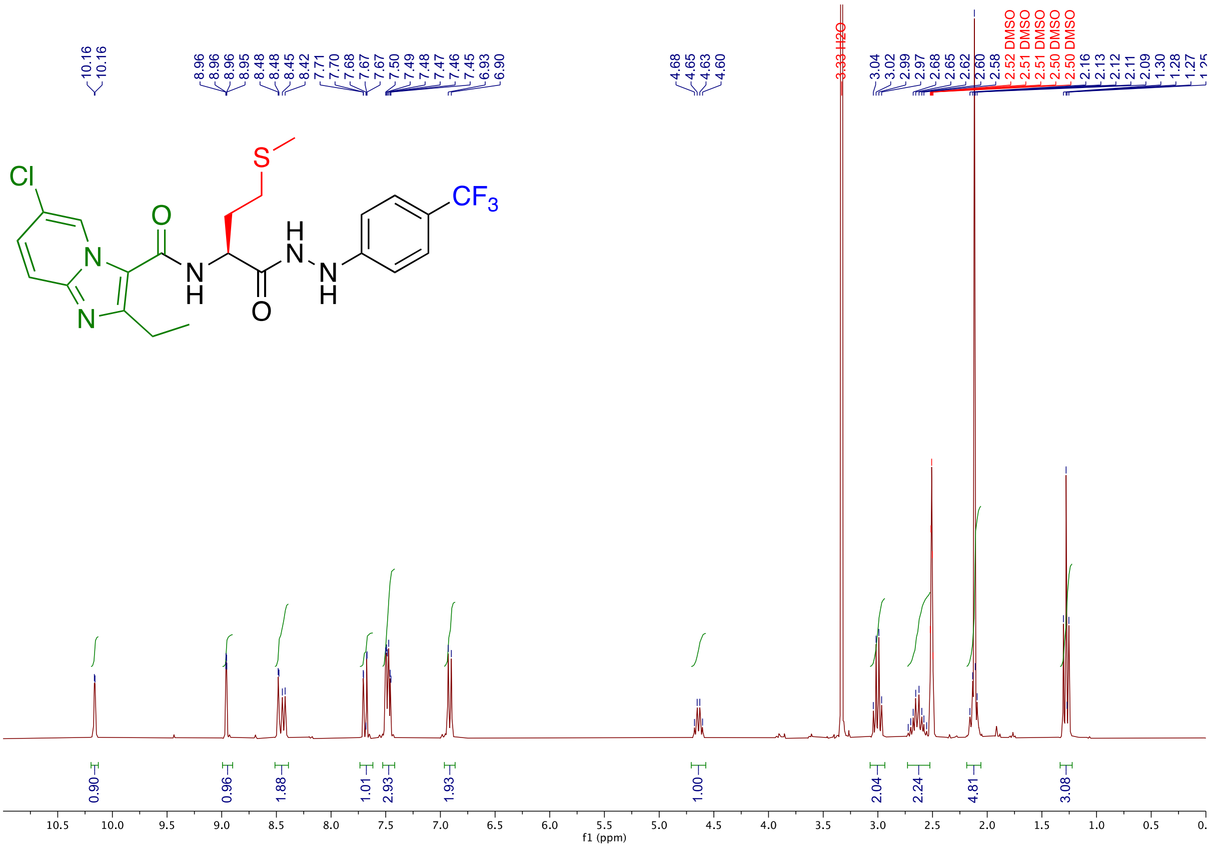

Cl
O
N
N
H
S
H
N
N
H
O
CF3
10.16
10.16
8.96
8.96
8.96
8.95
8.48
8.48
8.45
8.42
7.71
7.70
7.68
7.67
7.67
7.50
7.49
7.48
7.47
7.46
7.45
6.93
6.90
4.68
4.65
4.63
4.60
3.33 H2O
3.04
3.02
2.99
2.97
2.68
2.65
2.62
2.60
2.58
2.52 DMSO
2.51 DMSO
2.51 DMSO
2.50 DMSO
2.50 DMSO
2.16
2.13
2.12
2.11
2.09
1.30
1.28
1.27
0.90
0.96
1.88
1.01
2.93
1.93
1.00
2.04
2.24
4.81
3.08
10.5
10.0
9.5
9.0
8.5
8.0
7.5
7.0
6.5
6.0
5.5
5.0
4.5
4.0
3.5
3.0
2.5
2.0
1.5
1.0
0.5
f1 (ppm)

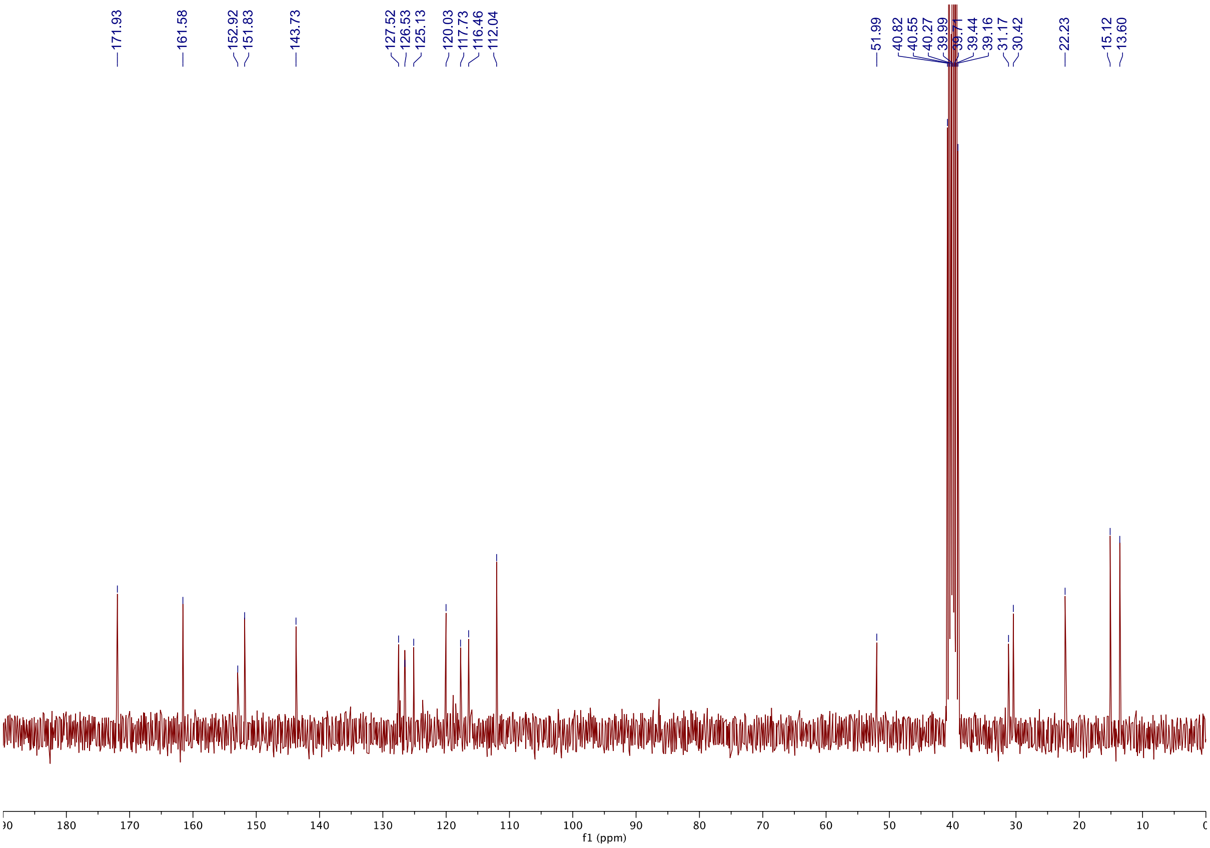

171.93
161.58
152.92
151.83
143.73
127.52
126.53
125.13
120.03
117.73
116.46
112.04
51.99
40.82
40.55
40.27
39.99
39.71
39.44
39.16
31.17
30.42
22.23
15.12
13.60
180
170
160
150
140
130
120
110
100
90
80
70
60
50
40
30
20
10
f1 (ppm)

Compound 36

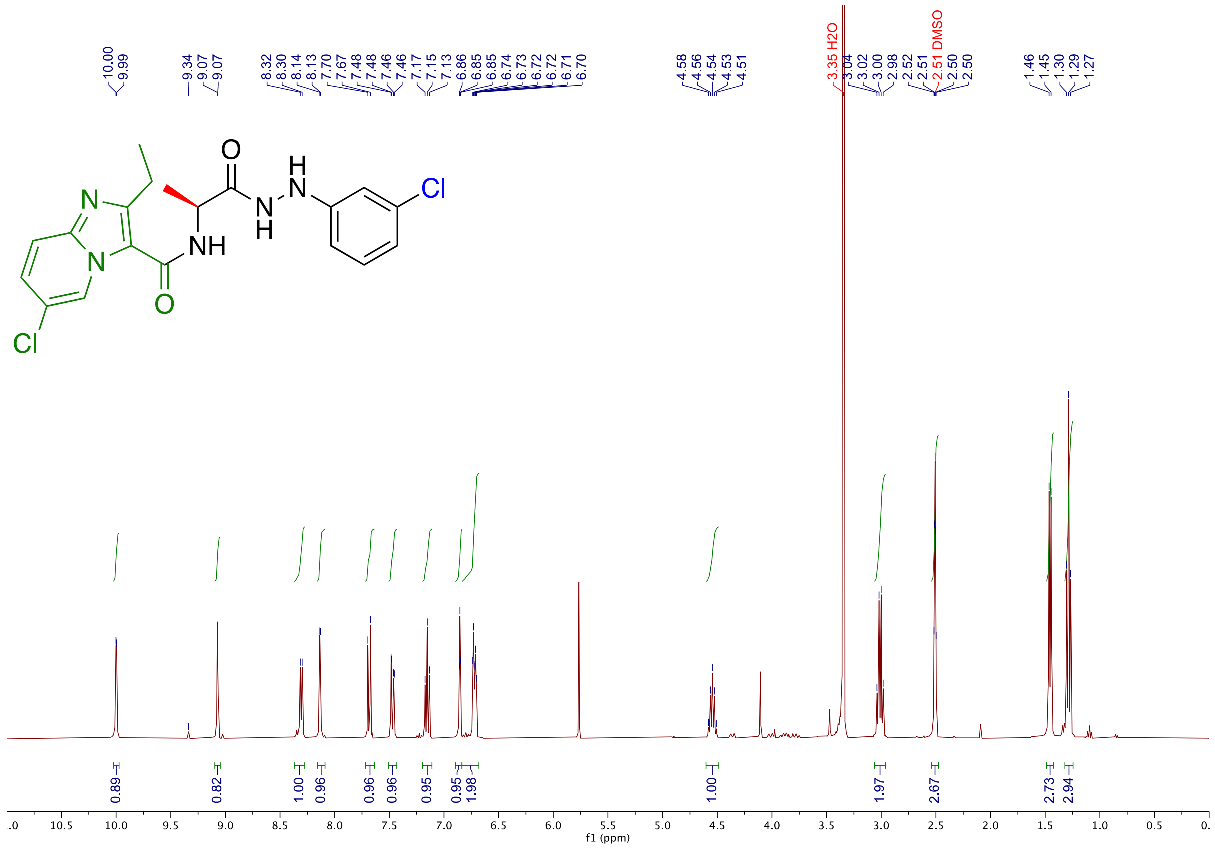
10.00
9.99
9.34
9.07
9.07
8.32
8.30
8.14
8.13
7.70
7.67
7.48
7.46
7.46
7.17
7.15
7.13
6.86
6.85
6.74
6.73
6.72
6.72
6.71
6.70
4.58
4.56
4.54
4.53
4.51
3.35 H2O
3.04
3.02
3.00
2.98
2.52
2.51
2.51 DMSO
2.50
2.50
1.46
1.45
1.30
1.29
1.27
O
H
N
N
H
Cl
N
NH
N
O
Cl
0.89
0.82
1.00
0.96
0.96
0.96
0.95
0.95
1.98
1.00
1.97
2.67
2.73
2.94
f1 (ppm)

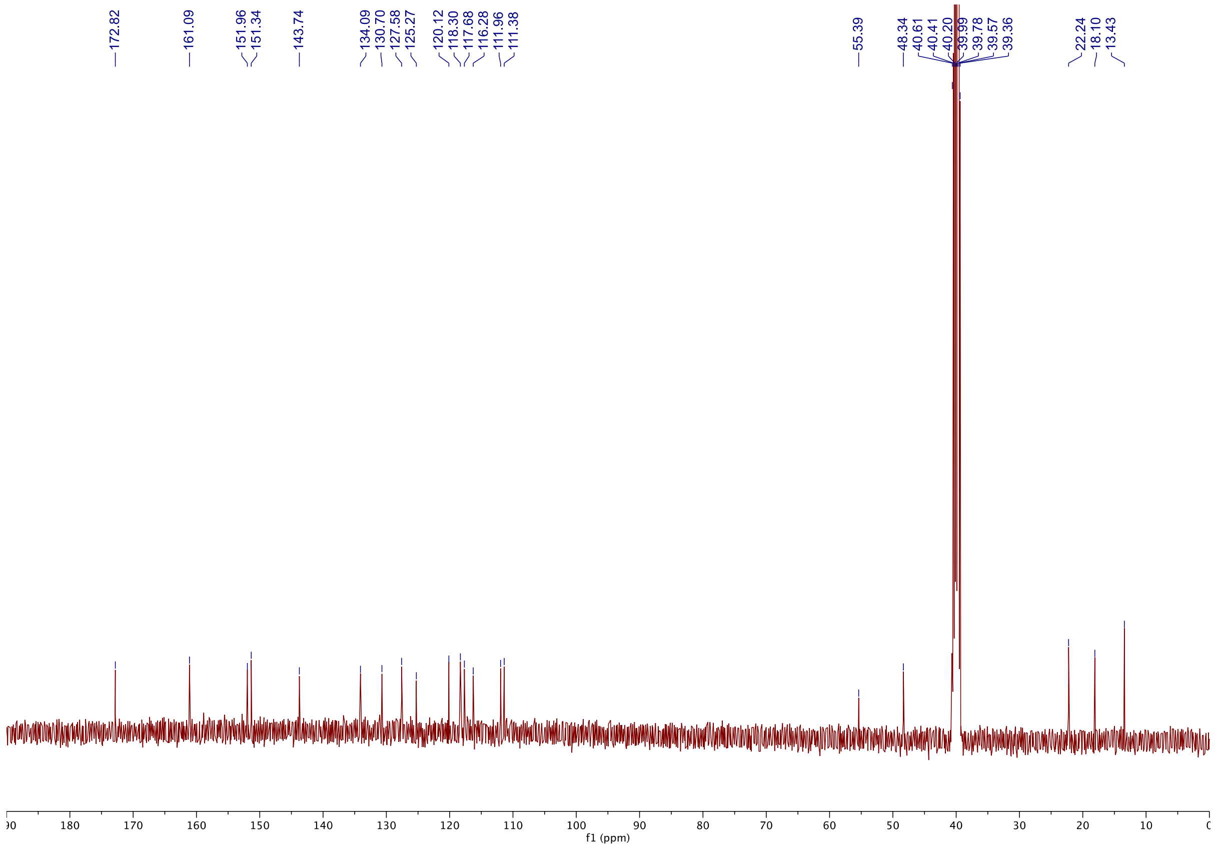
172.82
161.09
151.96
151.34
143.74
134.09
130.70
127.58
125.27
120.12
118.30
117.68
116.28
111.96
111.38
55.39
48.34
40.61
40.41
40.20
39.99
39.78
39.57
39.36
22.24
18.10
13.43
f1 (ppm)

Compound 37

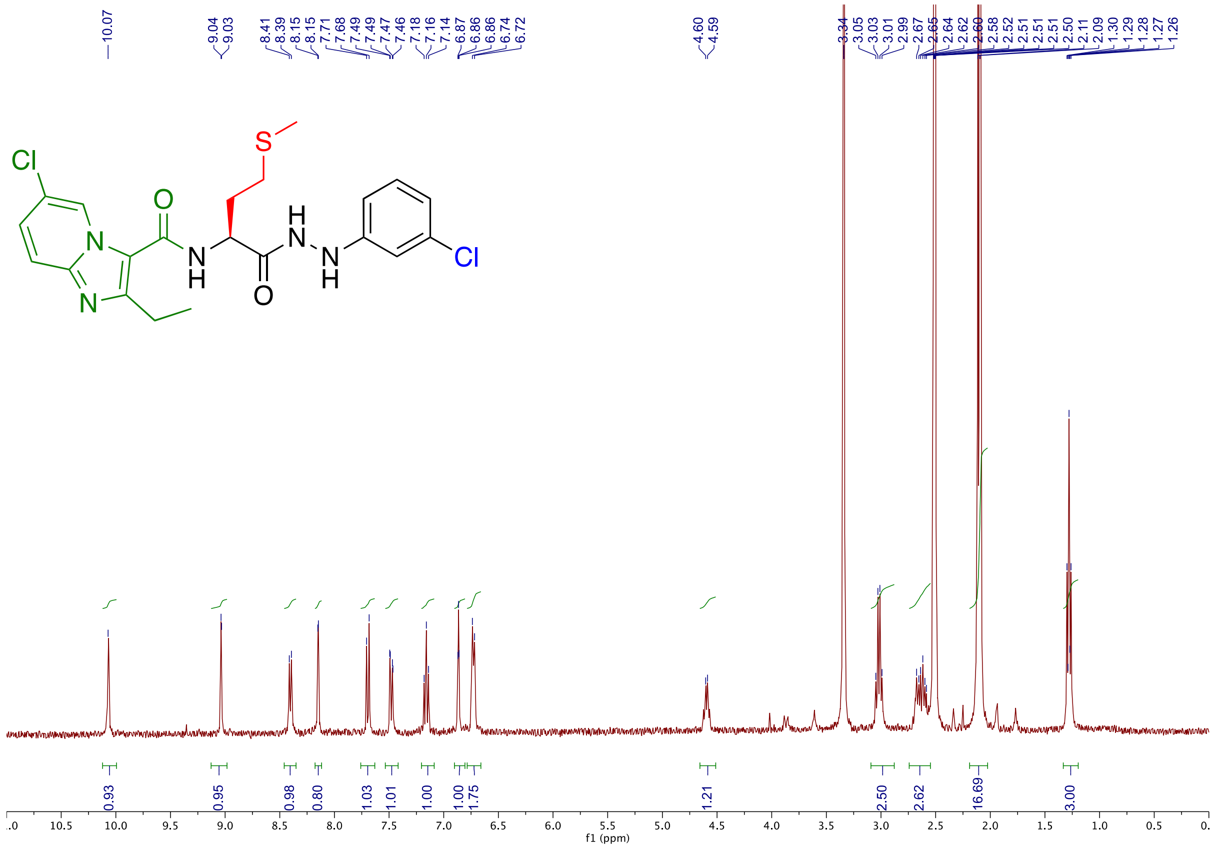

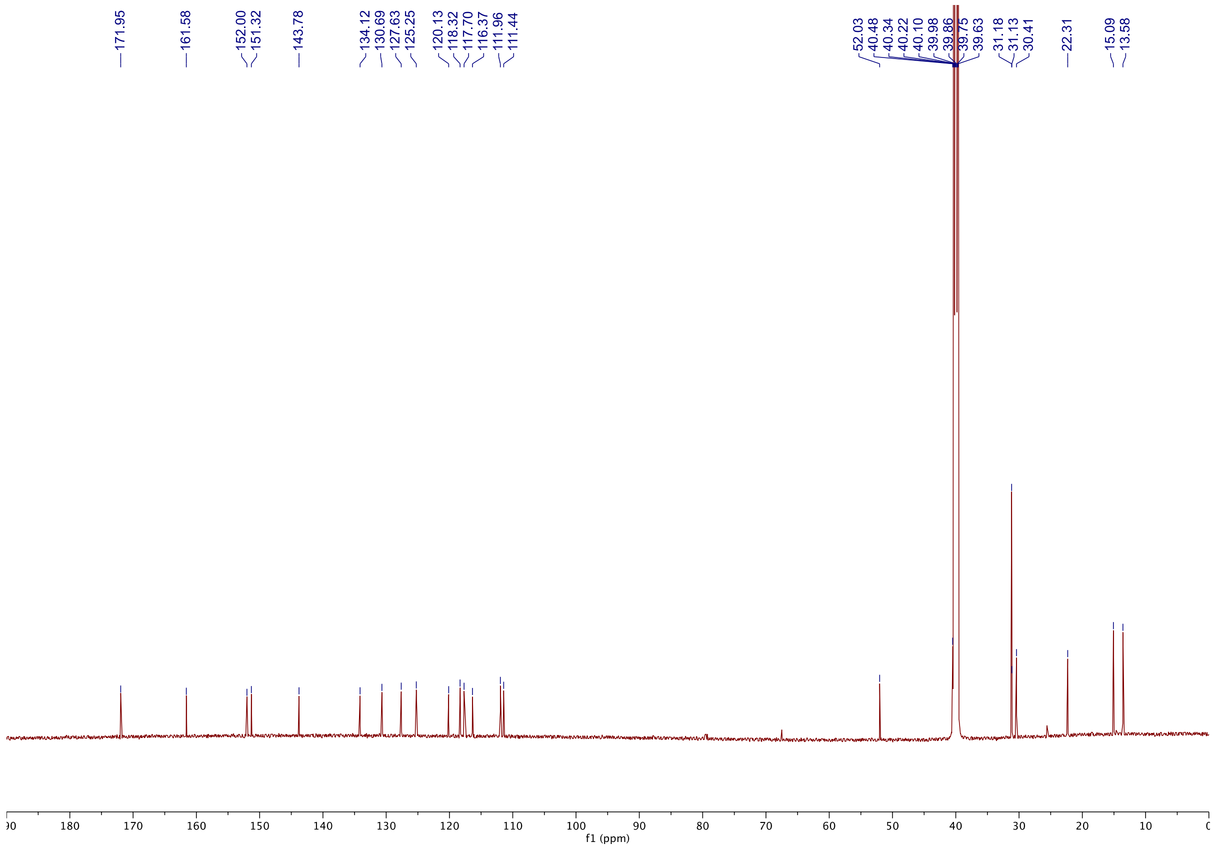

Supplement: Supplementary file 1 [file molecules-25-02387-s001.pdf]
